# Supplementary material for: Biomimetic Mn(III) porphyrin-catalyzed aromaticity-breaking epoxidation of electron-deficient naphthalene
Source: Nat Commun. 2025 Dec 22;16:11552. doi: 10.1038/s41467-025-67300-8 (PMC12749080; doi:10.1038/s41467-025-67300-8)
Supplement: Supplementary file 1 — Supplementary Information [file 41467_2025_67300_MOESM1_ESM.pdf]

Supporting Information

Biomimetic Mn(III) Porphyrins-Catalyzed Aromaticity-Breaking Epoxidation of Electron-Deficient Naphthalene

Hongli Wu[1#], Jialun Xu[1#], Junjie Tai[1#], Jingkun Gao[1], Yunfei Ge[1], Guijie Li[2*], Yuan-Bin She[2*], and Yun-Fang Yang[1*]

[1]State Key Laboratory of Advanced Separation Membrane Materials, College of Chemical Engineering, Zhejiang University of Technology, Hangzhou, Zhejiang 310014, China

[2]State Key Laboratory of Green Chemical Synthesis and Conversion, College of Chemical Engineering, Zhejiang University of Technology, Hangzhou, Zhejiang 310014, China

[#] *These authors contribute equally to this work*

*E-mail: guijieli@zjut.edu.cn

*E-mail: sheyb@zjut.edu.cn

*E-mail: yangyf@zjut.edu.cn

Table of Contents

1. Experimental Details S3
1.1. Synthesis and characterization S3
1.2. X-ray crystallography S3
1.3. Synthesis of Mn(TPFPP)Cl S3
1.4. Condition optimization S4
1.5. General procedure for oxidation reaction S6
1.6. Characterization data for epoxide 3a S6
1.7. Substrate expansion and date characterization S10
1.8. ^{1}H NMR, ^{13}C NMR and HRMS spectra S20
1.9. The reaction of 3a in the absence of catalyst S50
1.10. The reaction of naphthalene S52
1.11. The effect of substitution pattern S53
1.12. Mechanistic experiments S55
1.12.1. Monitoring the reaction of 1s S55
1.12.2. The kinetic isotope effect (KIE) experiments S57
2. Mechanistic Details S61
2.1. Possible spin states S61
2.2. NPA spin population of ^{5}INT2 .... S62
2.3. The other three possibilities for radical and electrophilic pathways S62
2.4. Possible hydrogen-atom transfer process S64
2.5. Geometries and NPA charges of ^{3}TS2 and ^{3}TS3 .... S64
2.6. The proton shuttle process from ^{3}INT4 .... S65
2.7. DFT-calculated free energy profile of oxidative functionalization of arene 1s . S65
2.8. DFT-calculated free energy profile of oxidative functionalization of arene 1t .. S66
2.9. The proton shuttle process from ^{3}INT9 .... S67
2.10. The origin of substituent-controlled selectivity switch S68
3. References S69

1. Experimental Details

1.1. Synthesis and characterization

Unless noted, all commercial reagents were purchased and used as received without further purification. 2,7-dimethoxynaphthalene-1,3,4,5,6,8-d_6 was purchased from Shanghai Leyan Scientific Co. Ltd. Other starting materials were purchased from commercial suppliers (J&K Scientific, Energy Chemical, Aladdin, Bide Pharmatech, Shanghai Leyan Scientific Co., Ltd., etc.) and used as received unless otherwise specified. ^{1}H NMR spectra were recorded at 500 or 600 MHz, and ^{13}C NMR spectra were recorded at 125 or 150 MHz NMR instruments in $CDCl_3$ or DMSO-d_6 solutions and chemical shifts were referenced to residual protiated solvent. If $CDCl_3$ was used as solvent, ^{1}H and ^{13}C NMR spectra were recorded with $CDCl_3$ (δ = 7.26 ppm) and (δ = 77.16 ppm) as internal references, respectively. If DMSO-d_6 was used as solvent, ^{1}H and ^{13}C NMR spectra were recorded with DMSO-d_6 (δ = 2.50 ppm) and (δ = 39.52 ppm) as internal references, respectively. The following abbreviations were used to explain ^{1}H NMR ultiplicities: s = singlet, d = doublet, t = triplet, q = quartet, p = quintet, m = multiplet, br = broad. All of the new compounds were analyzed for HRMS on a Waters mass spectrometer using electrospray ionization in of ESI-Q-TOF.

1.2. X-ray crystallography

Diffraction data was collected at 170.0 K on a Bruker SMART APEX diffractometer with Mo KR radiation (λ = 0.71073 Å).

1.3. Synthesis of Mn(TPFPP)Cl

Synthesis of TPFPP: Under a nitrogen atmosphere and protected from light, the following reagents were added sequentially to a 2 L round-bottom flask equipped with a magnetic stir bar: dichloromethane (1.5 L), freshly distilled pyrrole (1.0 g, 15 mmol, 1.0 equiv.), and pentafluorobenzaldehyde (5.88 g, 30 mmol, 2.0 equiv.). After 15 minutes of reaction, boron trifluoride diethyl etherate (0.71 g, 5.0 mmol, 0.33 equiv.) was added. The mixture was stirred for 3 hours, followed by the addition of 2,3-dichloro-5,6-dicyano-1,4-benzoquinone (6.81 g, 30 mmol, 2.0 equiv.) at room temperature. After an additional 3 hours of stirring, triethylamine (5.0 mL) was added, and the mixture was stirred for another 30 minutes. The crude product was purified by silica gel column chromatography (eluent: petroleum ether/dichloromethane = 2/1, *v*/*v*) to afford TPTPP as a purple solid (443 mg, 12% yield). ^{1}H NMR (500 MHz, $CDCl_3$) δ (ppm): –2.91 (s, 2H), 8.94 (s, 8H). HRMS (ESI) *m*/*z*: calcd for $C_{44}H_{11}F_{20}N_4^+$ $[M+H]^+$ 975.0659, found 975.0671. The ^{1}H NMR and HRMS spectra were consistent with previous research.[1]

Synthesis of Mn(TPFPP)Cl: The synthesis of Mn(TPFPP)Cl was based on previous studies.[1-3] In a 100 mL round-bottom flask equipped with a stir bar and under a nitrogen atmosphere, TPFPP (974 mg, 1 mmol, 10 equiv.) and manganese(II) acetate (1.76 g, 10 mmol, 10 equiv.) were charged. Dimethylformamide (50 mL) was added to the flask, and the reaction mixture was heated at 140 °C for 24 hours. After completion, the mixture was cooled to room temperature and treated with 10 mL of concentrated hydrochloric acid (36%). The resulting precipitate was collected by filtration and washed with deionized water until the filtrate became nearly colorless. The product was dried overnight under reduced pressure prior to use, affording 560 mg of a dark red solid (53% yield). HRMS (ESI) *m*/*z*: calcd for $C_{44}H_8F_{20}MnN_4^+$ [M–Cl]$^+$ 1026.9805, found 1026.9809. UV-visible (CH_3CN) λ_{max}: 363 nm, 475 nm, 574 nm. The UV-vis spectrum and HRMS data match the previous report.[3,4]

1.4. Condition optimization

A 38 mL oven-dried sealed tube (with a Teflon cap) equipped with a magnetic stir bar was charged with diethyl naphthaene-2,6-dicarboxylate (50 mg, 0.184 mmol, 1.0 equiv.), Mn(TPFPP)Cl, PhIO, and CH_3CN (2.0 mL) were added to the mixture. The tube was then capped and submerged into a preheated oil bath with variable temperature settings. The reaction was stirred for varying times before cooling down to room temperature. The reaction mixture was diluted with EtOAc (5.0 mL), and filtered through a short pad of Celite. The sealed tube and Celite pad were washed with an additional 10 mL of EtOAc. The filtrate was concentrated in vacuo, and the residue was analyzed by ^{1}H NMR spectrum using CH_2Br_2 as internal standard.

Table S1. Optimization of the amounts of oxidant PhIO[a].

Entry	PhIO(equiv.)	Conv./%	Selectivity/%[b, c]	
			**3a**	**5a'**
1	1.0	54	16 (30)	0.0 (0.0)
2	1.2	33	33 (100)	0.0 (0.0)
3	1.4	46	34 (74)	1.0 (2.2)
4	1.6	49	48 (98)	0.0 (0.0)
5	1.8	36	30 (83)	0.0 (0.0)
6	2.0	45	31 (69)	0.0 (0.0)
7	3.0	69	32 (46)	0.0 (0.0)
8	4.0	68	31 (46)	3.0 (4.4)

[a]Reaction condition: **1a** (0.184 mmol), Mn(TPFPP)Cl (1.0 mol%), at 80 °C for 2 h. [b]The yields were determined by ^{1}H NMR analyses of the crude products using CH_2Br_2 as the internal standard. [c]The yields indicated in parentheses were based on the conversion yields.

Table S2. Optimization of the amounts of catalyst Mn(TPFPP)Cl[a].

Entry	Mn(TPFPP)Cl (mol%)	Conv./%	Selectivity/%[b, c]	
			**3a**	**5a'**
1	0.2	47	43 (92)	0.0 (0.0)
2	0.4	59	29 (50)	0.0 (0.0)
3	0.6	51	45 (89)	0.0 (0.0)
4	0.8	33	23 (71)	0.0 (0.0)
5	1.0	45	44 (99)	0.0 (0.0)
6	1.5	47	40 (90)	0.0 (0.0)
7	2.0	42	40 (95)	0.0 (0.0)
8	3.0	50	38 (76)	0.0 (0.0)
9	4.0	30	19 (63)	0.0 (0.0)
10	5.0	27	16 (60)	0.0 (0.0)
11	10	18	15 (86)	0.0 (0.0)

[a]Reaction condition: **1a** (0.184 mmol), PhIO (0.269 mmol), at 80 °C for 1 h. [b]The yields were determined by ^1H NMR analyses of the crude products using CH_2Br_2 as the internal standard. [c]The yields indicated in parentheses were based on the conversion yields.

Table S3. Optimization of the reaction time[a].

Entry	reaction time	Conv./%	Selectivity/%[b, c]	
			**3a**	**5a'**
1	0.5	46	32 (70)	1.0 (2.0)
2	1	50	44 (99)	0.0 (0.0)
3	2	59	33 (56)	1.0 (1.7)
4	4	49	33 (68)	0.0 (0.0)
5	6	46	30 (66)	1.0 (2.2)
6	8	52	7.0 (14)	18 (34)
7	12	58	1.0 (1.7)	19 (33)
8	24	47	5.0 (11)	18 (38)
9	36	47	1.0 (2.1)	18 (38)
10	48	56	5.0 (9.0)	24 (43)
11	72	48	1.0 (2.0)	23 (48)

[a]Reaction condition: **1a** (0.184 mmol), Mn(TPFPP)Cl (1.0 mol%), PhIO (0.269 mmol), at 80 °C. [b]The yields were determined by ^1H NMR analyses of the crude products using CH_2Br_2 as the internal standard. [c]The yields indicated in parentheses were based on the conversion yields.

Table S4. Optimization of the reaction temperature[a].

Entry	T (°C)	Conv./%	Selectivity/%[b, c]	
			**3a**	**5a'**
1	0	6	6 (100)	0.0 (0.0)
2	20	30	25 (85)	0.0 (0.0)
3	30	55	43 (78)	0.0 (0.0)
4	40	65	50 (78)	0.0 (0.0)
5	50	52	47 (91)	0.0 (0.0)
6	60	57	48 (84)	0.0 (0.0)
7	70	47	40 (86)	0.0 (0.0)
8	80	45	44 (99)	0.0 (0.0)

[a]Reaction condition: **1a** (0.184 mmol), Mn(TPFPP)Cl (1.0 mol%), PhIO (0.269 mmol), 1 h. [b]The yields were determined by ^1H NMR analyses of the crude products using CH_2Br_2 as the internal standard. [c]The yields indicated in parentheses were based on the conversion yields.

1.5. General procedure for oxidation reaction

A 38 mL sealed tube (with a Teflon-lined cap) equipped with a magnetic stir bar was charged with substrate (1.0 mmol, 1.0 equiv), Mn(TPFPP)Cl (0.01 mmol, 1.0 mol%), PhIO (1.6 mmol, 1.6 equiv), and CH_3CN (5.0 mL). The tube was then sealed and immersed in a preheated oil bath at 50 °C. The reaction mixture was stirred for 1 hour at this temperature before being cooled to room temperature. The solvent was removed under reduced pressure to afford the crude product, which was subsequently purified by flash chromatography on silica gel using petroleum ether/ethyl acetate as the eluent to yield the target product.

1.6. Characterization data for epoxide 3a

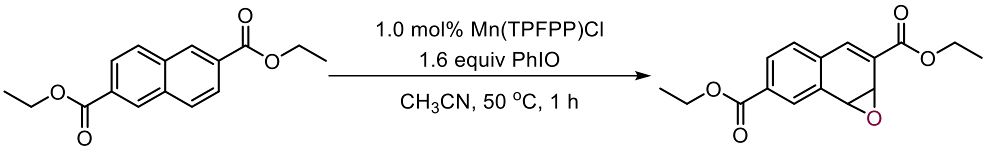

1a **3a**

A 38 mL oven-dried sealed tube (with a Teflon cap), equipped with a magnetic stir bar, was charged with diethyl-2,6-naphthalenedicarboxylate **1a** (545 mg, 2.0 mmol, 1.0 equiv.), Mn(TPFPP)Cl (21 mg, 0.02 mmol, 1.0 mol%) and PhIO (704 mg, 3.2 mmol, 1.6 equiv.) in CH_3CN (8.0 mL) at 50 °C for 1 h. The solution was cooled to room temperature, then concentrated under reduced pressure. The crude mixture was purified by silica gel column chromatography to recycle **1a** (180 mg) in 67.0% conversion, and provide the compound **3a** (petroleum ether/ethylacetate = 40/1, *v*/*v*) as a white solid 309 mg in 53.6% yield, which is

80.0% on the basis of 67.0% conversion. **3a**: ^{1}H NMR (500 MHz, DMSO-d_6) δ (ppm): 1.33 (t, J = 7.0 Hz, 3H), 1.35 (t, J = 7.0 Hz, 3H), 4.31 (q, J = 7.0 Hz, 2H), 4.36 (q, J = 7.0 Hz, 2H), 4.57 (dd, J = 4.0, 2.0 Hz 1H), 4.83 (d, J = 4.0 Hz, 1H), 7.84 (d, J = 2.5 Hz, 1H) 7.87 (d, J = 8.0 Hz, 1H), 8.05 (dd, J = 8.0, 2.0 Hz, 1H), 8.42 (d, J = 1.5 Hz, 1H). ^{13}C NMR (125 MHz, DMSO-d_6) δ (ppm): 14.13, 52.01, 55.92, 61.05, 61.14, 129.34, 129.72, 130.73, 131.12, 131.67, 134.26, 134.43, 136.30, 164.96, 165.18. HRMS (ESI) m/z: calcd for $C_{16}H_{16}NaO_5^+$ [M+Na]$^+$ 311.0890, found 311.0874.

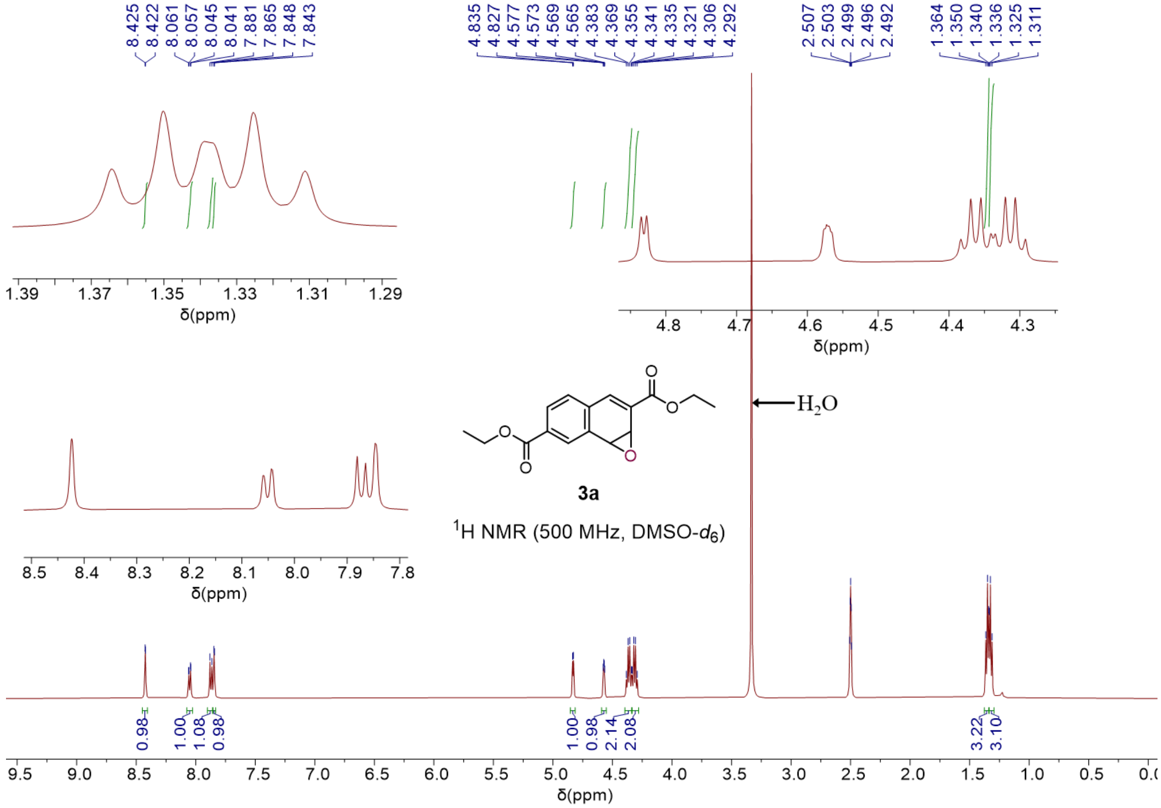

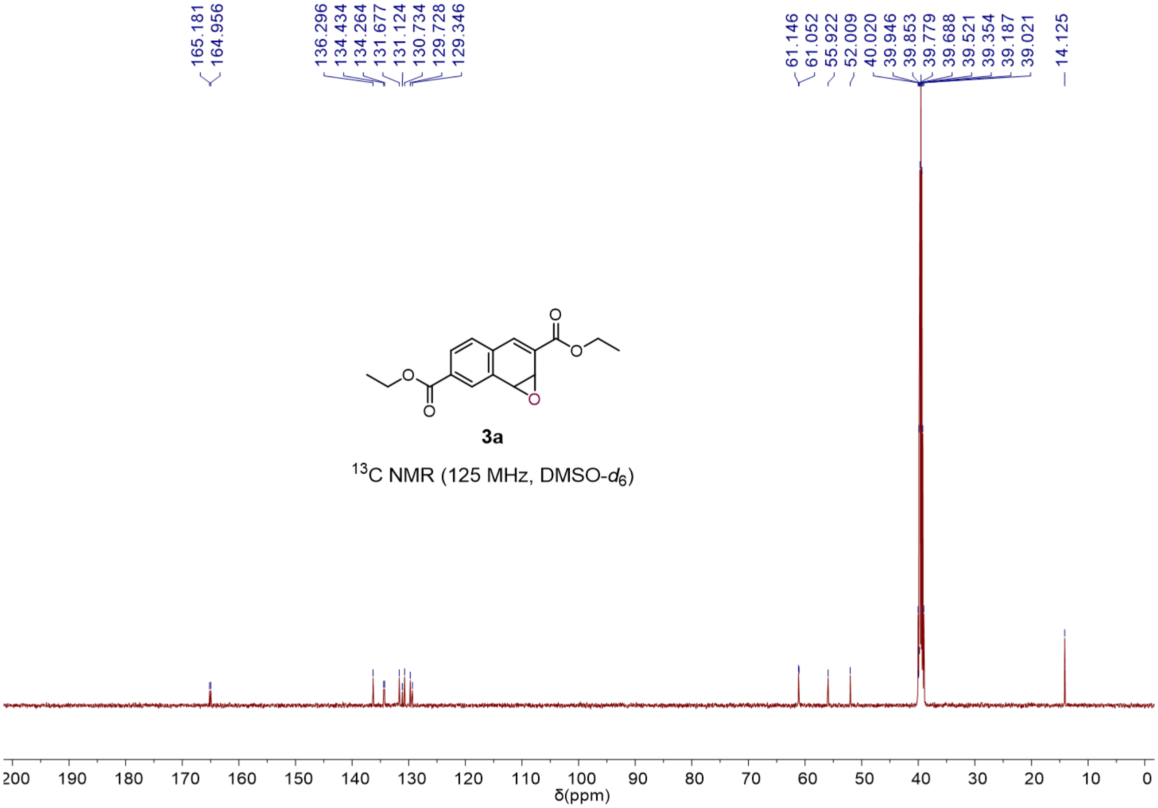

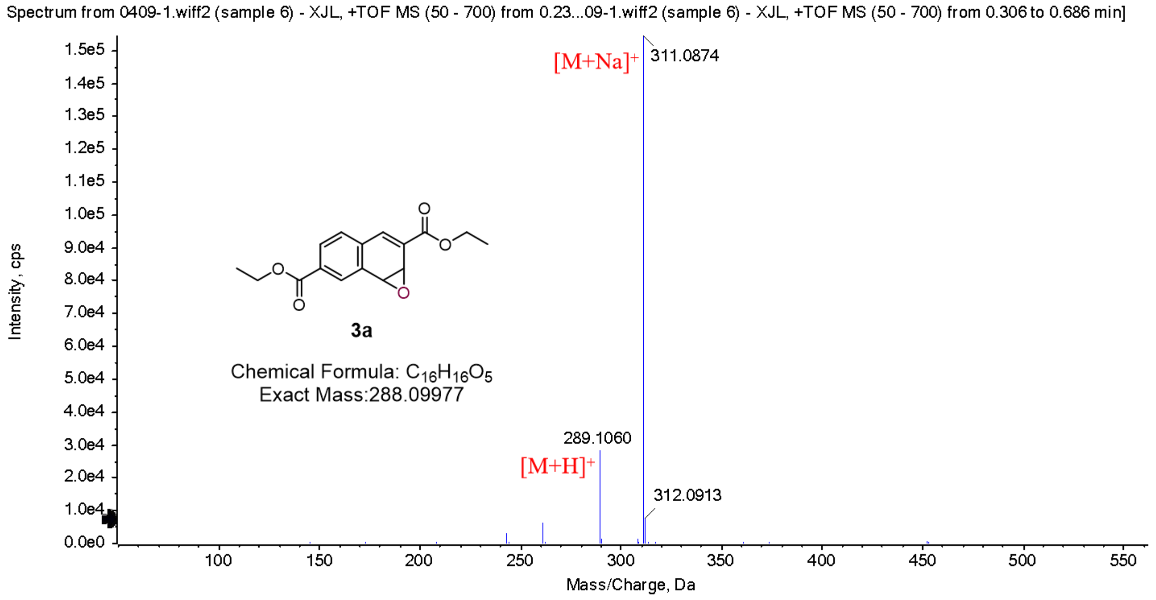

Ion Formula	Calculated *m/z*	Error (ppm)	Error (mmu)	RDB
$C_{16}H_{16}NaO_5^+$	311.08899	−5.3	−1.63	9.0

Table S5. Crystal data and structure refinement for 3a.

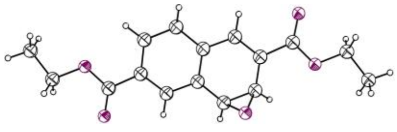

CCDC number	2404381
Empirical formula	$C_{16}H_{16}O_5$
Formula weight	288.29
Temperature/K	170.0
Crystal system	triclinic
Space group	P-1
a/Å	4.1823(2)
b/Å	8.1421(4)
c/Å	11.0336(6)
α/°	105.455(2)
β/°	95.567(2)
γ/°	101.862(2)
Volume/Å^3	349.79(3)
Z	1
ρ_{calc}g/cm^3	1.369
μ/mm^{-1}	0.102
F(000)	152.0
Crystal size/mm^3	0.48 × 0.13 × 0.08
Radiation	MoKα (λ = 0.71073)
2Θ range for data collection/°	5.348 to 54.33
Index ranges	-5 ≤ h ≤ 5, -10 ≤ k ≤ 10, -14 ≤ l ≤ 14
Reflections collected	7309
Independent reflections	1561 [R_{int} = 0.0244, R_{sigma} = 0.0233]
Data/restraints/parameters	1561/2/107
Goodness-of-fit on F^2	1.155
Final R indexes [I>=2σ (I)]	R_1 = 0.0456, wR_2 = 0.1282
Final R indexes [all data]	R_1 = 0.0480, wR_2 = 0.1297
Largest diff. peak/hole / e Å^{-3}	0.24/-0.21

1.7. Substrate expansion and date characterization

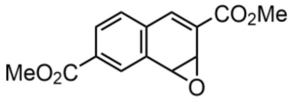

3b

The title compound was prepared according to general procedure from dimethyl naphthalene-2,6-dicarboxylate (244 mg, 1.0 mmol, 1.0 equiv.). The crude mixture was purified by silica gel column chromatography to recycle naphthalene-2,6-dicarboxylate (142 mg) in 41.8% conversion, and provide the compound **3b** (petroleum ether/ethylacetate = 40/1, *v*/*v*) as a white solid 73 mg in 28.1% yield, which is 67.1% on the basis of 41.8% conversion. ^{1}H NMR (500 MHz, $CDCl_3$) δ (ppm): 3.92 (s, 3H), 3.96 (s, 3H), 4.58 (d, J = 4.0 Hz, 1H), 4.71 (dd, J = 4.0, 2.0 Hz, 1H), 7.56 (d, J = 8.0 Hz, 1H), 7.77 (d, J = 2.0 Hz, 1H), 8.11 (dd, J = 8.0, 1.5 Hz, 1H), 8.36 (t, J = 2.0, 1.0 Hz, 1H). ^{13}C NMR (125 MHz, $CDCl_3$) δ (ppm): 52.54, 52.63, 52.77, 57.05, 129.51, 130.41, 131.34, 131.40, 131.67, 134.27, 134.84, 137.20, 166.20, 166.34. HRMS (ESI) *m*/*z*: calcd for $C_{14}H_{12}NaO_5^+$ $[M+Na]^+$ 283.0577, found 283.0574.

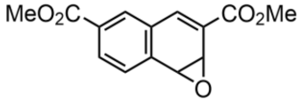

3c

The title compound was prepared according to general procedure from dimethyl naphthalene-2,7-dicarboxylate (244 mg, 1.0 mmol, 1.0 equiv.). The crude mixture was purified by silica gel column chromatography to recycle dimethyl naphthalene-2,7-dicarboxylate (100 mg) in 59.0% conversion, and provide the compound **3c** (petroleum ether/ethylacetate = 35/1, *v*/*v*) as a white solid 98 mg in 37.7% yield, which is 63.9% on the basis of 59.0% conversion. ^{1}H NMR (500 MHz, $CDCl_3$) δ (ppm): 3.91 (s, 3H), 3.95 (s, 3H), 4.55 (d, J = 3.5, 1H), 4.71 (dd, J = 4.0, 2.0 Hz, 1H), 7.77 (d, J = 8.0 Hz, 1H), 7.80 (d, J = 2.0 Hz, 1H), 8.12 (dd, J = 8.0, 2.0 Hz, 1H), 8.17 (d, J = 2.0 Hz, 1H). ^{13}C NMR (125 MHz, $CDCl_3$) δ (ppm): 52.49, 53.00, 53.16, 56.75, 127.92, 130.43, 131.19, 131.28, 132.52, 137.68, 138.53, 166.22, 166.39. HRMS (ESI) *m*/*z*: calcd for $C_{14}H_{12}NaO_5^+$ $[M+Na]^+$ 283.0577, found 283.0583.

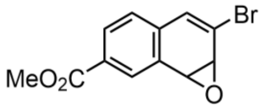

3d

The title compound was prepared according to general procedure from methyl 6-bromo-2-naphthoate (265 mg, 1.0 mmol, 1.0 equiv.). The crude mixture was purified by silica gel column chromatography to recycle methyl 6-bromo-2-naphthoate (103 mg) in 61.1% conversion, and provide the compound **3d** (petroleum ether/ethylacetate = 50/1, *v*/*v*) as a

white solid 31 mg in 11.1% yield, which is 18.0% on the basis of 61.1% conversion. ^{1}H NMR (500 MHz, $CDCl_3$) δ (ppm): 3.95 (s, 3H), 4.71 (dd, J = 4.0, 2.0 Hz, 1H), 4.55 (d, J = 3.5 Hz, 1H), 7.11 (d, J = 2.0 Hz, 1H), 7.32 (d, J = 8.0 Hz, 1H), 8.06 (dd, J = 8.0, 2.0 Hz, 1H), 8.30 (d, J = 1.5 Hz, 1H). ^{13}C NMR (125 MHz, DMSO-d_6) δ (ppm): 52.38, 58.20, 58.28, 123.48, 128.60, 129.07, 130.10, 130.66, 130.87, 131.15, 136.11, 165.65. HRMS (ESI) m/z: calcd for $C_{12}H_9BrNaO_3^+$ $[M+Na]^+$ 302.9627, found 302.9631.

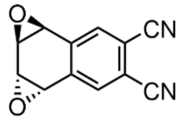

4e

The title compound was prepared according to general procedure from naphthalene-2,3-dicarbonitrile (178 mg, 1.0 mmol, 1.0 equiv.). The crude mixture was purified by silica gel column chromatography to recycle naphthalene-2,3-dicarbonitrile (110 mg) in 38.2% conversion, and provide the compound **4e** (petroleum ether/ethylacetate = 5/1, *v*/*v*) as a white solid 58 mg in 27.6% yield, which is 72.3% on the basis of 38.2% conversion. ^{1}H NMR (500 MHz, DMSO-d_6) δ (ppm): 3.40 (m, 2H), 4.18 (m, 2H), 8.37 (s, 2H). ^{13}C NMR (125 MHz, DMSO-d_6) δ (ppm): 47.32, 54.80, 115.35, 115.42, 136.01, 138.53. HRMS (ESI) m/z: calcd for $C_{12}H_5N_2O_2^-$ $[M–H]^-$ 209.0357, found 209.0351.

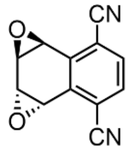

4f

The title compound was prepared according to general procedure from naphthalene-1,4-dicarbonitrile (178 mg, 1.0 mmol, 1.0 equiv). The crude mixture was purified by silica gel column chromatography to recycle naphthalene-1,4-dicarbonitrile (139 mg) in 21.9% conversion, and provide the compound **4f** (petroleum ether/ethylacetate = 5/1, *v*/*v*) as a white solid 26 mg in 12.4% yield, which is 56.5% on the basis of 21.9% conversion. ^{1}H NMR (500 MHz, DMSO-d_6) δ (ppm): 4.11 (m, 2H), 4.26 (m, 2H), 8.14 (s, 2H). ^{13}C NMR (125 MHz, DMSO-d_6) δ (ppm): 47.98, 54.33, 115.38, 118.49, 134.24, 136.48. HRMS (ESI) m/z: calcd for $C_{12}H_5N_2O_2^-$ $[M–H]^-$ 209.0357, found 209.0364.

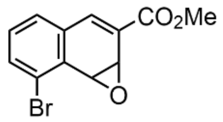

3g

The title compound was prepared according to general procedure from methyl 5-bromo-2-naphthoate (265 mg, 1.0 mmol, 1.0 equiv.). The crude mixture was purified by

silica gel column chromatography to recycle methyl 5-bromo-2-naphthoate (123 mg) in 53.6% conversion, and provide the compound **3g** (petroleum ether/ethylacetate = 60/1, *v*/*v*) as a white solid 12 mg in 4.3% yield, which is 8.0% on the basis of 53.6% conversion. ^{1}H NMR (500 MHz, DMSO-d_6) δ (ppm): 3.84 (s, 3H), 4.50 (dd, J = 4.0, 2.0 Hz, 1H), 4.85 (d, J = 4.0 Hz, 1H), 7.45 (t, J = 8.0 Hz, 1H), 7.75 (d, J = 7.5 Hz, 1H), 7.79 (d, J = 2.0 Hz, 1H), 7.81 (dd, J = 8.0, 1.0 Hz, 1H). ^{13}C NMR (125 MHz, DMSO-d_6) δ (ppm): 52.37, 52.53, 56.31, 125.25, 127.23, 131.05, 131.43, 132.26, 132.81, 134.66, 137.31, 165.67. HRMS (ESI) *m*/*z*: calcd for $C_{12}H_9BrNaO_3^+$ [M+Na]$^+$ 302.9627, found 302.9638.

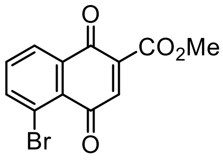

5g

The title compound was prepared according to general procedure from methyl 5-bromo-2-naphthoate (265 mg, 1.0 mmol, 1.0 equiv.). The crude mixture was purified by silica gel column chromatography to recycle methyl 5-bromo-2-naphthoate (123 mg) in 53.6% conversion, and provide the compound **5g** (petroleum ether/ethylacetate = 60/1, *v*/*v*) as a yellow solid 10 mg in 3.4% yield, which is 6.3% on the basis of 53.6% conversion. ^{1}H NMR (500 MHz, DMSO-d_6) δ (ppm): 3.94 (s, 3H), 7.83 (s, 1H), 8.20 (d, J = 8.0 Hz, 1H), 8.37 (dd, J = 8.0, 2.0 Hz, 1H), 8.43 (d, J = 2.0 Hz, 1H). ^{13}C NMR (125 MHz, DMSO-d_6) δ (ppm): 52.96, 126.56, 127.84, 131.84, 133.78, 134.11, 134.35, 139.61, 140.45, 164.85, 177.34, 181.73. HRMS (ESI) *m*/*z*: calcd for $C_{12}H_7BrNaO_4^+$ [M+Na]$^+$ 316.9420, found 316.9429.

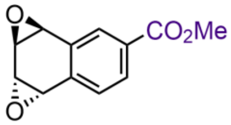

4h

The title compound was prepared according to general procedure from methyl 2-naphthoate (186 mg, 1.0 mmol, 1.0 equiv.). The crude mixture was purified by silica gel column chromatography to recycle methyl 2-naphthoate (105 mg) in 43.5% conversion, and provide the compound **4h** (petroleum ether/ethylacetate = 35/1, *v*/*v*) as a white solid 16 mg in 7.4% yield, which is 16.9% on the basis of 43.5% conversion. ^{1}H NMR (500 MHz, DMSO-d_6) δ (ppm): 3.87 (s, 3H), 3.94 (d, J = 4.0 Hz, 1H), 4.00 (d, J = 4.0 Hz, 1H), 4.09 (ddd, J = 4.0, 2.0, 0.5 Hz, 1H), 4.10 (ddd, J = 4.0, 2.0, 0.5 Hz, 1H), 7.70 (d, J = 8.0 Hz, 1H), 7.96 (dd, J = 8.0, 2.0 Hz, 1H), 8.13 (d, J = 2.0 Hz, 1H). ^{13}C NMR (125 MHz, DMSO-d_6) δ (ppm): 51.53, 51.69, 52.53, 54.80, 55.17, 130.70, 131.29, 131.72, 132.23, 132.54, 136.68, 166.20. HRMS (ESI) *m*/*z*: calcd for $C_{12}H_{10}NaO_4^+$ [M+Na]$^+$ 241.0471, found 241.0467.

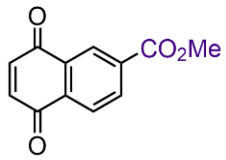

5h

The title compound was prepared according to general procedure from methyl 2-naphthoate (186 mg, 1.0 mmol, 1.0 equiv.). The crude mixture was purified by silica gel column chromatography to recycle methyl 2-naphthoate (105 mg) in 43.5% conversion, and provide the compound **5h** (petroleum ether/ethylacetate = 30/1, *v*/*v*) as a yellow solid 10 mg in 4.6% yield, which is 10.6% on the basis of 43.5% conversion. ^{1}H NMR (500 MHz, $CDCl_3$) δ (ppm): 4.00 (s, 1H), 7.05 (m, 2H), 8.18 (d, J = 8.0 Hz, 1H), 8.41 (dd, J = 8.0, 2.0 Hz, 1H), 8.74 (d, J = 1.5 Hz, 1H). The ^{1}H NMR data was in agreement with that reported in the literature[5].

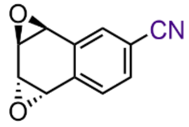

4i

The title compound was prepared according to general procedure from 2-naphthonitrile (153 mg, 1.0 mmol, 1.0 equiv.). The crude mixture was purified by silica gel column chromatography to recycle 2-naphthonitrile (55 mg) in 64.1% conversion, and provide the compound **4i** (petroleum ether/ethylacetate = 25/1, *v*/*v*) as a white solid 89 mg in 48.1% yield, which is 75.1% on the basis of 64.1% conversion. ^{1}H NMR (500 MHz, DMSO-d_6) δ (ppm): 3.92 (d, J = 4.0 Hz, 1H), 3.95 (d, J = 4.0 Hz, 1H), 4.12 (m, 2H), 7.77 (d, J = 8.0 Hz, 1H), 7.88 (dd, J = 8.0, 2.0 Hz, 1H), 8.06 (d, J = 2.0 Hz, 1H). ^{13}C NMR (125 MHz, DMSO-d_6) δ (ppm): 49.86, 50.06, 54.30, 54.68, 111.94, 118.10, 132.38, 133.19, 133.64, 134.61, 137.53. HRMS (ESI) *m*/*z*: calcd for $C_{11}H_8NO_2^+$ $[M+H]^+$ 186.0550, found 186.0550.

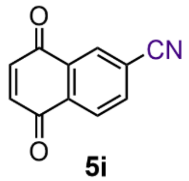

5i

The title compound was prepared according to general procedure from 2-naphthonitrile (153 mg, 1.0 mmol, 1.0 equiv.). The crude mixture was purified by silica gel column chromatography to recycle 2-naphthonitrile (55 mg) in 64.0% conversion, and provide the compound **5i** (petroleum ether/ethylacetate = 10/1, *v*/*v*) as a yellow solid 7 mg in 3.8% yield, which is 6.0% on the basis of 64.0% conversion. ^{1}H NMR (500 MHz, $CDCl_3$) δ (ppm): 7.09 (s, 1H), 8.03 (dd, J = 8.0, 1.5 Hz, 1H), 8.21 (d, J = 8.0 Hz, 1H), 8.39 (d, J = 2.0 Hz, 1H). The ^{1}H NMR data was in agreement with that reported in the literature[6].

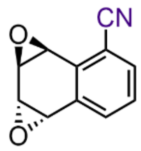

4j

The title compound was prepared according to general procedure from 1-naphthonitrile (153 mg, 1.0 mmol, 1.0 equiv.). The crude mixture was purified by silica gel column chromatography to recycle 1-naphthonitrile (63 mg) in 58.8% conversion, and provide the compound **4j** (petroleum ether/ethylacetate = 20/1, *v*/*v*) as a white solid 66 mg in 35.7% yield, which is 60.7% on the basis of 58.8% conversion. ^1H NMR (500 MHz, DMSO-d_6) δ (ppm): 3.97 (d, J = 4.0 Hz, 1H), 4.02 (d, J = 4.5 Hz, 1H), 4.14 (dd, J = 4.0, 2.0 Hz, 1H), 4.18 (dd, J = 4.0, 2.0, 1H), 7.61 (t, J = 8.0 Hz, 1H), 7.91 (m, 2H). ^{13}C NMR (125 MHz, DMSO-d_6) δ (ppm): 48.36, 49.96, 53.95, 54.67, 114.17, 116.45, 130.27, 133.46, 133.66, 135.16, 136.21. HRMS (ESI) m/z: calcd for $C_{11}H_8NO_2^+$ [M+H]$^+$ 186.0550, found 186.0551.

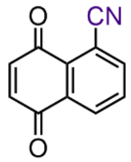

5j

The title compound was prepared according to general procedure from 1-naphthonitrile (153 mg, 1.0 mmol, 1.0 equiv.). The crude mixture was purified by silica gel column chromatography to recycle 1-naphthonitrile (63 mg) in 58.8% conversion, and provide the compound **5j** (petroleum ether/ethylacetate = 10/1, *v*/*v*) as a yellow solid 10 mg in 5.5% yield, which is 9.3% on the basis of 58.8% conversion. ^1H NMR (500 MHz, $CDCl_3$) δ (ppm): 7.09 (dd, J = 19.0, 10.5 Hz, 1H), 7.90 (t, J = 2.0 Hz, 1H), 8.10 (dd, J = 2.5, 1.0 Hz, 1H), 8.39 (dd, J = 8.0, 4.0 Hz, 1H). ^{13}C NMR (125 MHz, $CDCl_3$) δ (ppm): 110.97, 130.79, 133.94, 138.34, 139.14, 140.11. HRMS (ESI) m/z: calcd for $C_{11}H_5NNaO_2^+$ [M+Na]$^+$ 186.0550, found 186.0551.

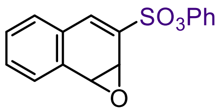

3k

The title compound was prepared according to general procedure from phenyl naphthalene-2-sulfonate (284 mg, 1.0 mmol, 1.0 equiv.). The crude mixture was purified by silica gel column chromatography to recycle phenyl naphthalene-2-sulfonate (136 mg) in 52.1% conversion, and provide the compound **3k** (petroleum ether/ethylacetate = 20/1, *v*/*v*) as a white solid 51 mg in 17.0% yield, which is 32.6% on the basis of 52.1% conversion. ^1H NMR (500 MHz, DMSO-d_6) δ (ppm): 4.70 (dd, J = 4.0, 2.5 Hz, 1H), 4.87 (d, J = 4.0 Hz, 1H),

7.28 (m, 2H), 7.35 (m, 1H), 7.44 (m, 2H), 7.56 (td, *J* = 7.5, 1.5 Hz, 1H), 7.65 (td, *J* = 7.5, 1.5 Hz, 1H), 7.74 (d, *J* = 2.5 Hz, 1H), 7.78 (d, *J* = 7.0 Hz, 1H), 7.95 (d, *J*= 7.5 Hz, 1H). ^{13}C NMR (125 MHz, DMSO-d_6) δ (ppm): 52.21, 57.67, 123.00, 127.56, 129.25, 129.97, 130.68, 131.16, 132.01, 132.25, 133.79, 139.32, 149.63. HRMS (ESI) *m*/*z*: calcd for $C_{16}H_{13}O_4S^+$ [M+H]$^+$ 301.0529, found 301.0528.

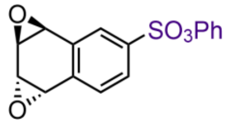

4k

The title compound was prepared according to general procedure from phenyl naphthalene-2-sulfonate (284 mg, 1.0 mmol, 1.0 equiv.). The crude mixture was purified by silica gel column chromatography to recycle phenyl naphthalene-2-sulfonate (136 mg) in 52.1% conversion, and provide the compound **4k** (petroleum ether/ethylacetate = 5/1, *v*/*v*) as a white solid 81 mg in 25.6% yield, which is 49.2% on the basis of 52.1% conversion. ^{1}H NMR (500 MHz, DMSO-d_6) δ (ppm): 3.99 (d, *J*= 4.0 Hz, 1H), 4.06 (d, *J* =4.0 Hz, 1H), 4.12 (ddd, *J* =4.0, 2.0, 0.5 Hz, 1H), 4.14(ddd, *J* =4.0, 2.0, 1.0 Hz, 1H), 7.07 (m, 2H), 7.34 (m, 1H), 7.41 (m, 2H), 7.85 (m, 2H), 8.15 (d, *J* =2.0 Hz, 1H). ^{13}C NMR (125 MHz, DMSO-d_6) δ (ppm): 49.79, 49.88, 54.40, 54.79, 121.97, 127.66, 129.14, 130.17, 130.55, 132.71, 134.26, 135.03, 139.21, 148.91. HRMS (ESI) *m*/*z*: calcd for $C_{16}H_{11}O_5S^-$ [M–H]$^-$ 315.0333, found 315.0332.

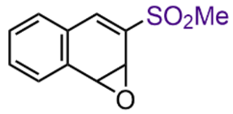

3l

The title compound was prepared according to general procedure from 2-(methylsulfonyl)naphthalene (206 mg, 1.0 mmol, 1.0 equiv.). The crude mixture was purified by silica gel column chromatography to recycle phenyl naphthalene-2-sulfonate (102 mg) in 50.5% conversion, and provide the compound **3l** (petroleum ether/ethylacetate = 45/10, *v*/*v*) as a white solid 25 mg in 11.3% yield, which is 22.3% on the basis of 50.5% conversion. ^{1}H NMR (500 MHz, DMSO-d_6) δ (ppm): 3.27(s, 3H), 4.67 (dd, *J* = 4.0, 2.0 Hz, 1H), 4.79 (d, *J* = 4.0 Hz, 1H), 7.57 (td, *J* = 7.5, 1.5 Hz, 1H), 7.61 (td, *J* = 7.0, 1.5 Hz, 1H), 7.73 (d, *J* = 2.5 Hz, 1H), 7.80 (d, *J* = 7.0 Hz, 1H), 7.90 (d, *J* = 7.0 Hz, 1H). ^{13}C NMR (125 MHz, DMSO-d_6) δ (ppm): 41.88, 51.30, 56.42, 129.41, 129.64, 130.52, 131.38, 131.96, 133.44, 135.81, 136.79. HRMS (ESI) *m*/*z*: calcd for $C_{11}H_9O_3S^-$ [M–H]$^-$ 221.0278, found 221.0275.

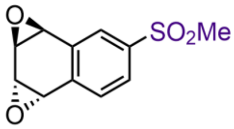

4l

The title compound was prepared according to general procedure from 2-(methylsulfonyl)naphthalene (206 mg, 1.0 mmol, 1.0 equiv.). The crude mixture was purified by silica gel column chromatography to recycle phenyl naphthalene-2-sulfonate (102 mg) in 50.5% conversion, and provide the compound **4l** (petroleum ether/ethylacetate = 3/1, *v*/*v*) as a white solid 72 mg in 30.2% yield, which is 59.9% on the basis of 50.5% conversion. ^{1}H NMR (500 MHz, DMSO-d_6) δ (ppm): 3.25 (s, 3H), 3.80 (d, J = 4.0, Hz, 1H), 4.03 (d, J =4.0 Hz, 1H), 4.14 (m, 2H), 7.83 (d, J =8.0 Hz, 1H), 7.93 (dd, J =8.0, 2.0 Hz, 1H), 8.15 (d, J =2.0 Hz, 1H). ^{13}C NMR (125 MHz, DMSO-d_6) δ (ppm): 43.36, 50.05, 50.12, 54.41, 54.72, 127.83, 129.63, 132.40, 133.67, 137.75, 141.47. HRMS (ESI) *m*/*z*: calcd for $C_{11}H_9O_4S^-$ [M–H]$^-$ 237.0227, found 237.0223.

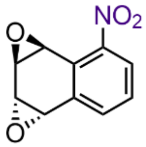

4m

The title compound was prepared according to general procedure from 1-nitronaphthalene (173 mg, 1.0 mmol, 1.0 equiv.). The crude mixture was purified by silica gel column chromatography to recycle 1-nitronaphthalene (96 mg) in 44.5% conversion, and provide the compound **4k** (petroleum ether/ethylacetate = 10/1, *v*/*v*) as a white solid 68 mg in 33.2% yield, which is 74.6% on the basis of 44.5% conversion. ^{1}H NMR (500 MHz, DMSO-d_6) δ (ppm): 4.04 (dd, J = 4.0, 1.0 Hz, 1H), 4.06 (m, 1H), 4.09 (ddd, J = 4.0, 2.0, 1.0 Hz, 1H), 4.09 (ddd, J = 4.0, 2.0, 0.5 Hz, 1H), 7.66 (dd, J = 8.0, 7.5 Hz, 1H), 7.95 (dd, J =7.5, 1.0 Hz, 1H), 8.02 (dd, J = 7.5, 1.0 Hz, 1H). ^{13}C NMR (125 MHz, DMSO-d_6) δ (ppm): 46.35, 50.09, 52.97, 54.18, 125.16, 126.21, 130.43, 134.28, 136.41, 151.47. HRMS (ESI) *m*/*z*: calcd for $C_{10}H_6NO_4^-$ [MN–H]$^-$ 204.0302, found 204.0299.

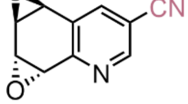

4n

The title compound was prepared according to general procedure from quinoline-3-carbonitrile (154 mg, 1.0 mmol, 1.0 equiv.). The crude mixture was purified by silica gel column chromatography to recycle quinoline-3-carbonitrile (90 mg) in 41.6% conversion, and provide the compound **4n** (petroleum ether/ethylacetate = 15/1, *v*/*v*) as a

white solid 51 mg in 27.5% yield, which is 66.0% on the basis of 41.5% conversion. ^{1}H NMR (500 MHz, DMSO-d_6) δ (ppm): 3.94 (d, J = 4.0 Hz, 1H), 4.00 (dd, J = 4.0, 0.5 Hz, 1H), 4.18 (ddd, J = 4.0, 2.0, 0.5 Hz, 1H), 4.23 (ddd, J = 4.0, 2.0, 0.5 Hz, 1H), 8.54 (d, J = 2.0 Hz, 1H), 8.98 (d, J = 2.0 Hz, 1H). ^{13}C NMR (125 MHz, DMSO-d_6) δ (ppm): 49.14, 51.88, 54.35, 55.82, 109.45, 116.44, 128.69, 142.47, 152.41, 156.21. HRMS (ESI) m/z: calcd for $C_{10}H_7N_2O_2^+$ $[M+H]^+$ 187.0502, found 187.0503.

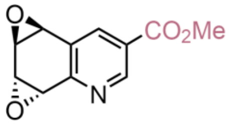

4o

The title compound was prepared according to general procedure from methyl quinoline-3-carboxylate (187 mg, 1.0 mmol, 1.0 equiv.). The crude mixture was purified by silica gel column chromatography to recycle methyl quinoline-3-carboxylate (120 mg) in 35.8% conversion, and provide the compound **4o** (petroleum ether/ethylacetate = 35/1, *v*/*v*) as a yellow solid 31 mg in 14.1% yield, which is 39.5% on the basis of 35.8% conversion. ^{1}H NMR (500 MHz, DMSO-d_6) δ (ppm): 3.90 (s, 3H), 3.93 (d, J = 4.0 Hz, 1H), 4.11 (dd, J = 4.0, 0.5 Hz, 1H), 4.17 (ddd, J = 4.0, 2.0, 0.5 Hz, 1H), 4.12 (ddd, J = 4.0, 2.0, 0.5 Hz, 1H), 8.52 (d, J = 2.0 Hz, 1H), 9.00 (d, J = 2.5 Hz, 1H). ^{13}C NMR (125 MHz, DMSO-d_6) δ (ppm): 49.43, 51.97, 52.71, 54.28, 55.74, 125.95, 128.46, 139.60, 149.80, 156.42, 164.65. HRMS (ESI) m/z: calcd for $C_{11}H_9NO_4^+$ $[M+H]^+$ 220.0604, found 220.0611.

Table S6. Crystal data and structure refinement for 4e.

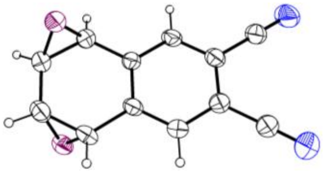

CCDC number	2482279
Empirical formula	$C_{12}H_6N_2O_2$
Formula weight	210.19
Temperature/K	170.00
Crystal system	orthorhombic
Space group	Pccn
a/Å	34.182(3)
b/Å	4.2906(4)
c/Å	19.6873(15)
α/°	90
β/°	90
γ/°	90
Volume/Å^3	2887.4(4)
Z	12
ρ_{calc}g/cm^3	1.451
μ/mm^{-1}	0.560
F(000)	1296.0
Crystal size/mm^3	0.35 × 0.06 × 0.04
Radiation	GaKα (λ = 1.34139)
2Θ range for data collection/°	4.498 to 108.39
Index ranges	-40 ≤ h ≤ 41, -5 ≤ k ≤ 4, -20 ≤ l ≤ 23
Reflections collected	21632
Independent reflections	2662 [R_{int} = 0.0899, R_{sigma} = 0.0739]
Data/restraints/parameters	2662/0/217
Goodness-of-fit on F^2	1.074
Final R indexes [I>=2σ (I)]	R_1 = 0.0686, wR_2 = 0.1692
Final R indexes [all data]	R_1 = 0.0908, wR_2 = 0.1866
Largest diff. peak/hole / e Å^{-3}	0.54/-0.32

Table S7. Crystal data and structure refinement for 4j.

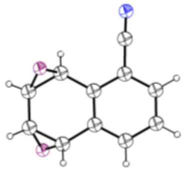

CCDC number	2485413
Empirical formula	$C_{11}H_7NO_2$
Formula weight	185.18
Temperature/K	170.00
Crystal system	monoclinic
Space group	$P2_1/n$
a/Å	4.2865(2)
b/Å	6.8290(3)
c/Å	29.7662(12)
α/°	90
β/°	90.724(2)
γ/°	90
Volume/Å^3	871.26(7)
Z	4
ρ_{calc}g/cm^3	1.412
μ/mm^{-1}	0.539
F(000)	384.0
Crystal size/mm^3	0.45 × 0.11 × 0.08
Radiation	GaKα (λ = 1.34139)
2Θ range for data collection/°	10.342 to 113.862
Index ranges	-5 ≤ h ≤ 5, -8 ≤ k ≤ 8, -37 ≤ l ≤ 36
Reflections collected	10105
Independent reflections	1725 [R_{int} = 0.0380, R_{sigma} = 0.0431]
Data/restraints/parameters	1725/0/127
Goodness-of-fit on F^2	1.137
Final R indexes [I>=2σ (I)]	R_1 = 0.0502, wR_2 = 0.1312
Final R indexes [all data]	R_1 = 0.0526, wR_2 = 0.1326
Largest diff. peak/hole / e Å^{-3}	0.27/-0.22

1.8. ^{1}H NMR, ^{13}C NMR and HRMS spectra

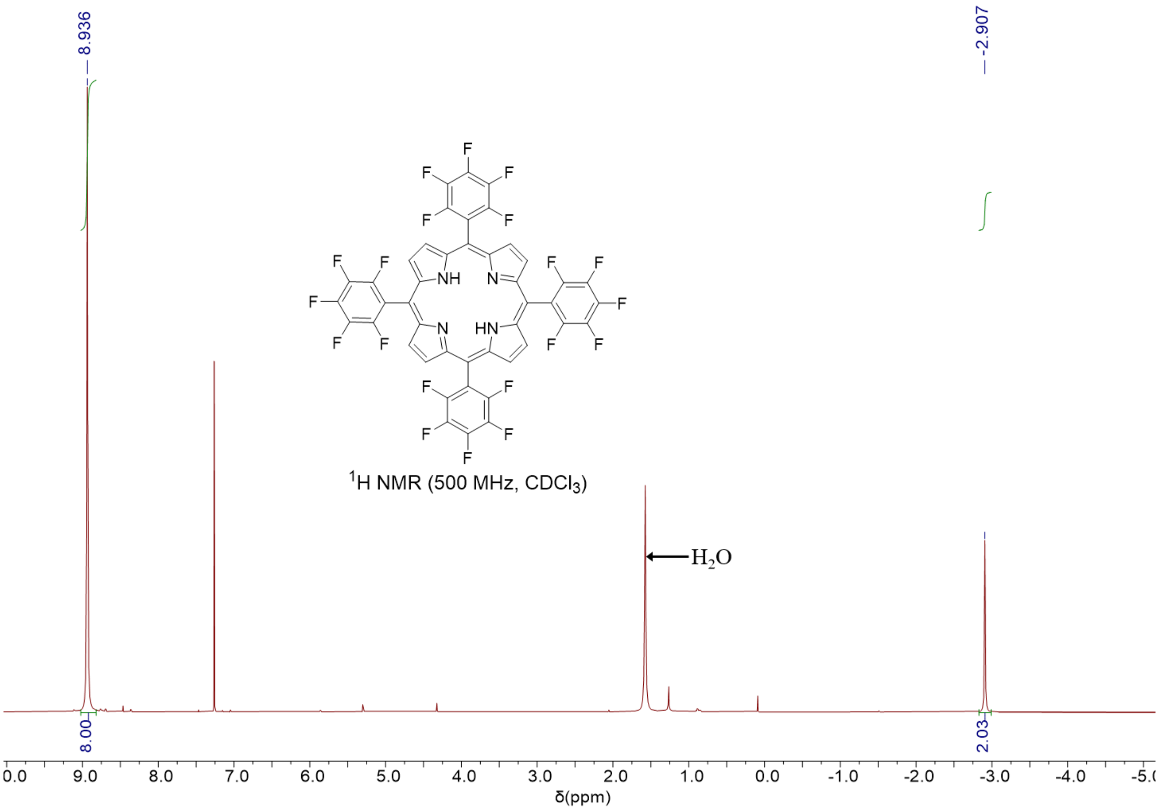

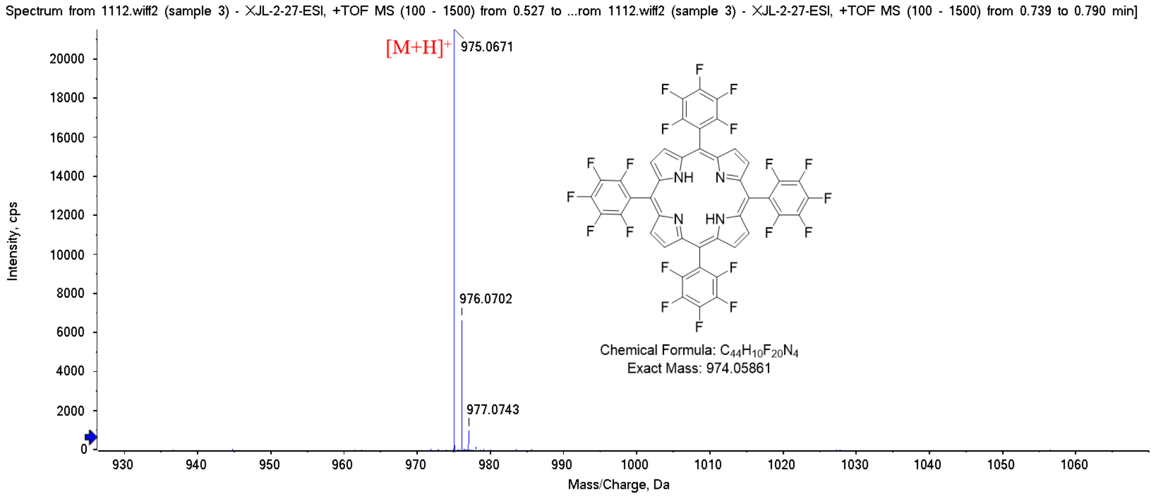

Ion Formula	Calculated *m/z*	Error (ppm)	Error (mmu)	RDB
$C_{44}H_{11}F_{20}MnN_4$	975.06589	1.3	1.24	32.0

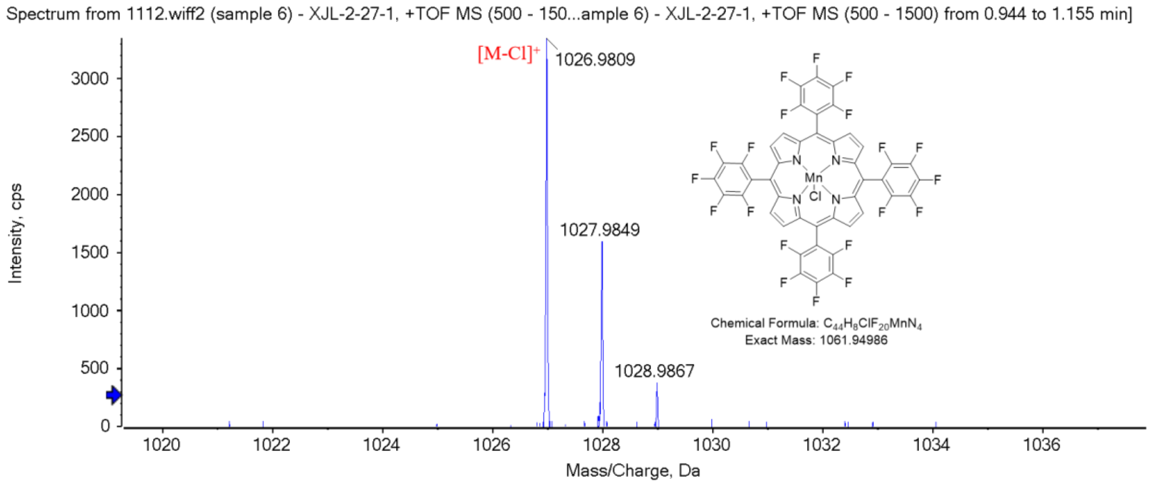

Ion Formula	Calculated *m/z*	Error (ppm)	Error (mmu)	RDB
$C_{44}H_8F_{20}MnN_4$	1026.98045	0.5	0.47	33.5

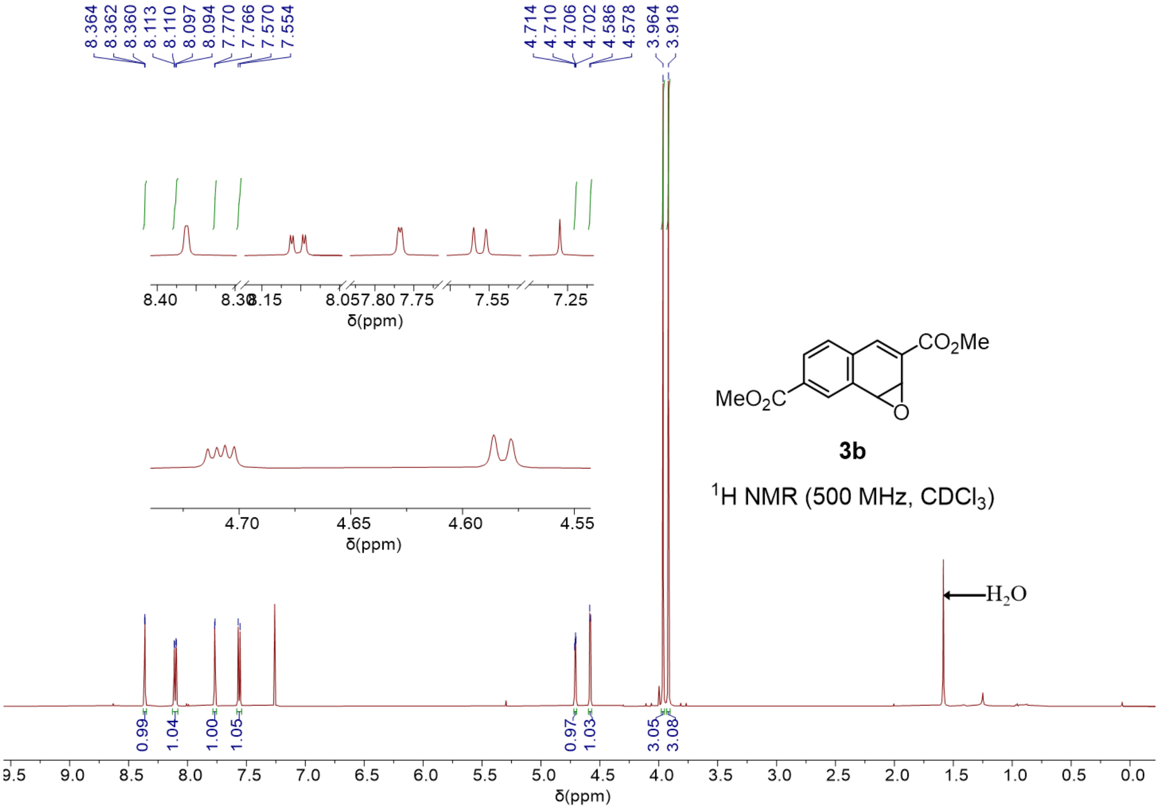

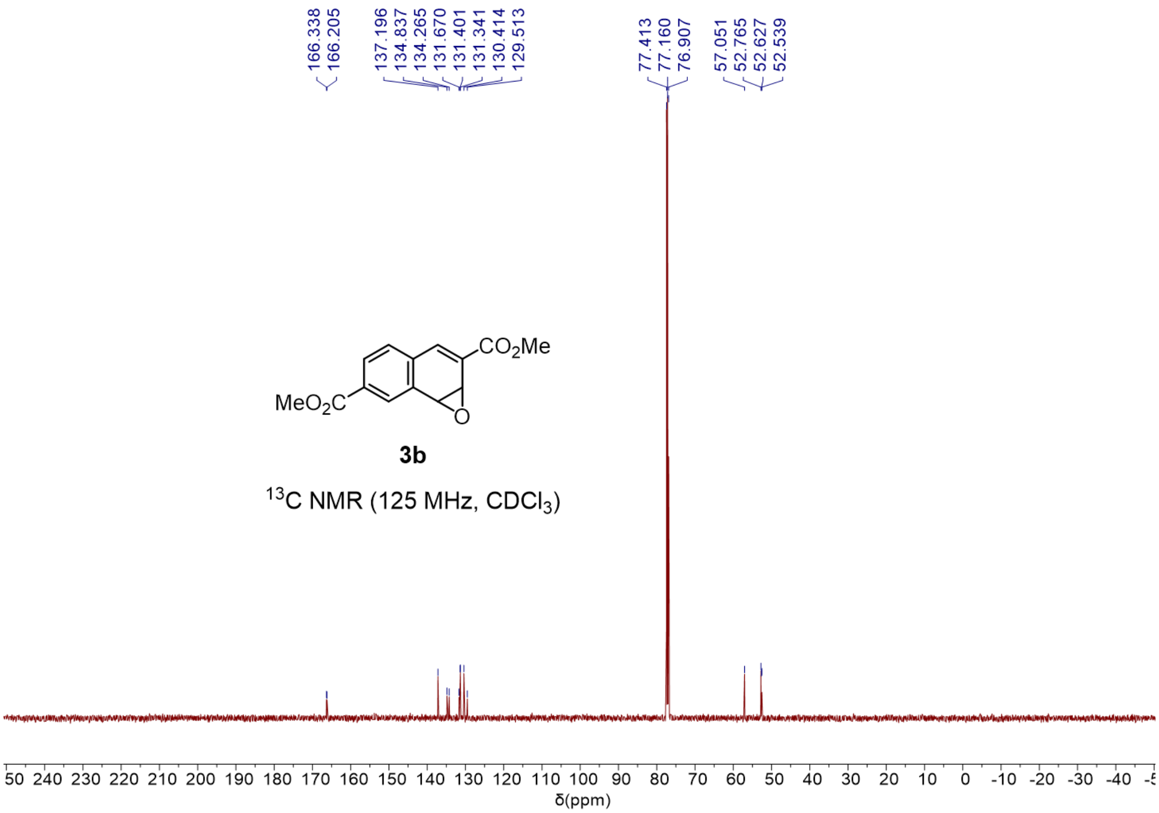

3b

^{13}C NMR (125 MHz, $CDCl_3$)

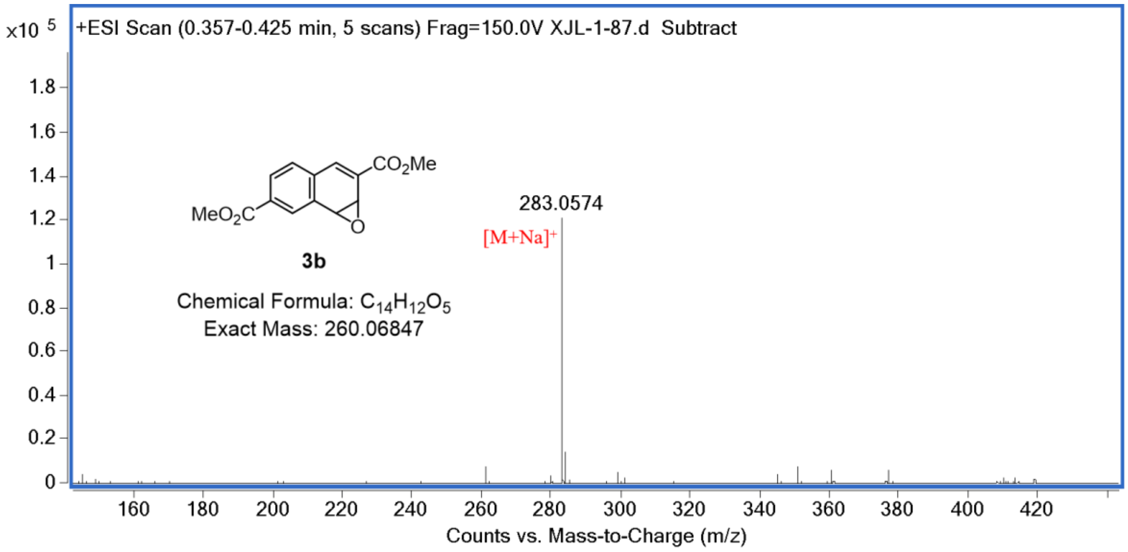

Formula (M)	Ion Formula	*m*/*z*	Calc *m*/*z*	Diff (ppm)	DBE
$C_{14}H_{12}O_5$	$C_{14}H_{12}NaO_5$	283.0574	283.0577	1.13	9

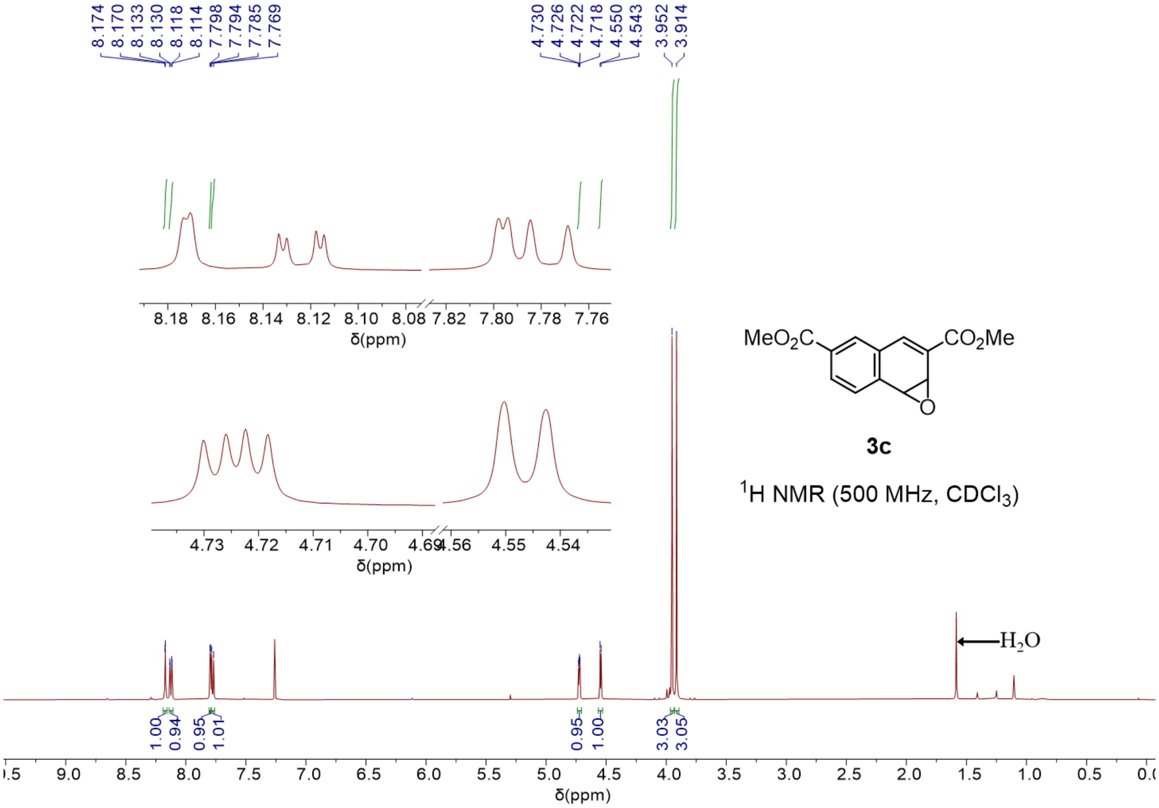
8.174
8.170
8.133
8.130
8.118
8.114
7.798
7.794
7.785
7.769
4.730
4.726
4.722
4.718
4.550
4.543
3.952
3.914
MeO2C
CO2Me
O
3c
1H NMR (500 MHz, CDCl3)
H2O
1.00
0.94
0.95
1.01
0.95
1.00
3.03
3.05
δ(ppm)

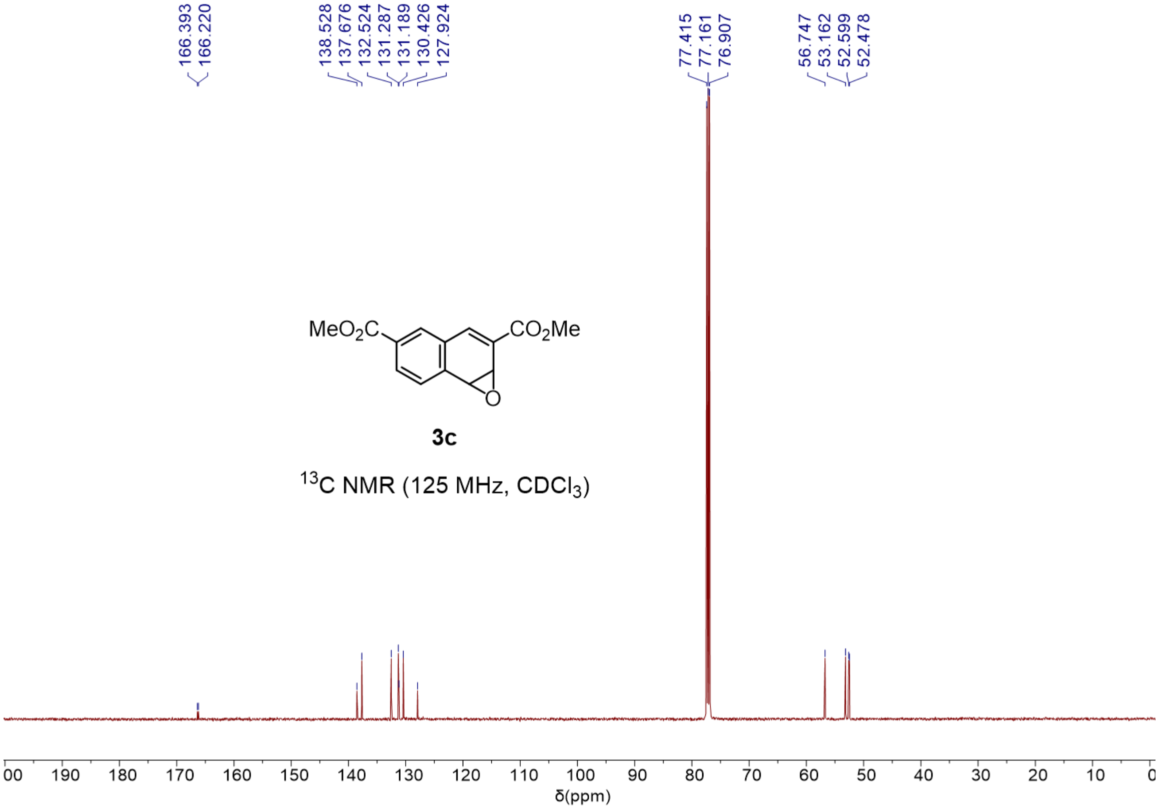
166.393
166.220
138.528
137.676
132.524
131.287
131.189
130.426
127.924
77.415
77.161
76.907
56.747
53.162
52.599
52.478
MeO2C
CO2Me
O
3c
13C NMR (125 MHz, CDCl3)
δ(ppm)

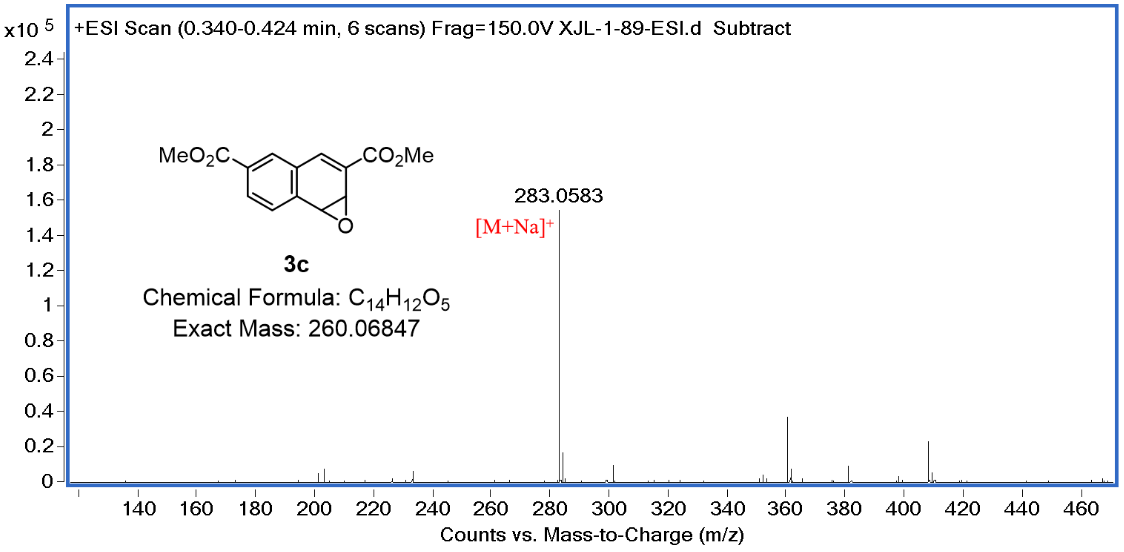

Formula (M)	Ion Formula	*m*/*z*	Calc *m*/*z*	Diff (ppm)	DBE
$C_{14}H_{12}O_5$	$C_{14}H_{12}NaO_5$	283.0583	283.0577	-2.33	9

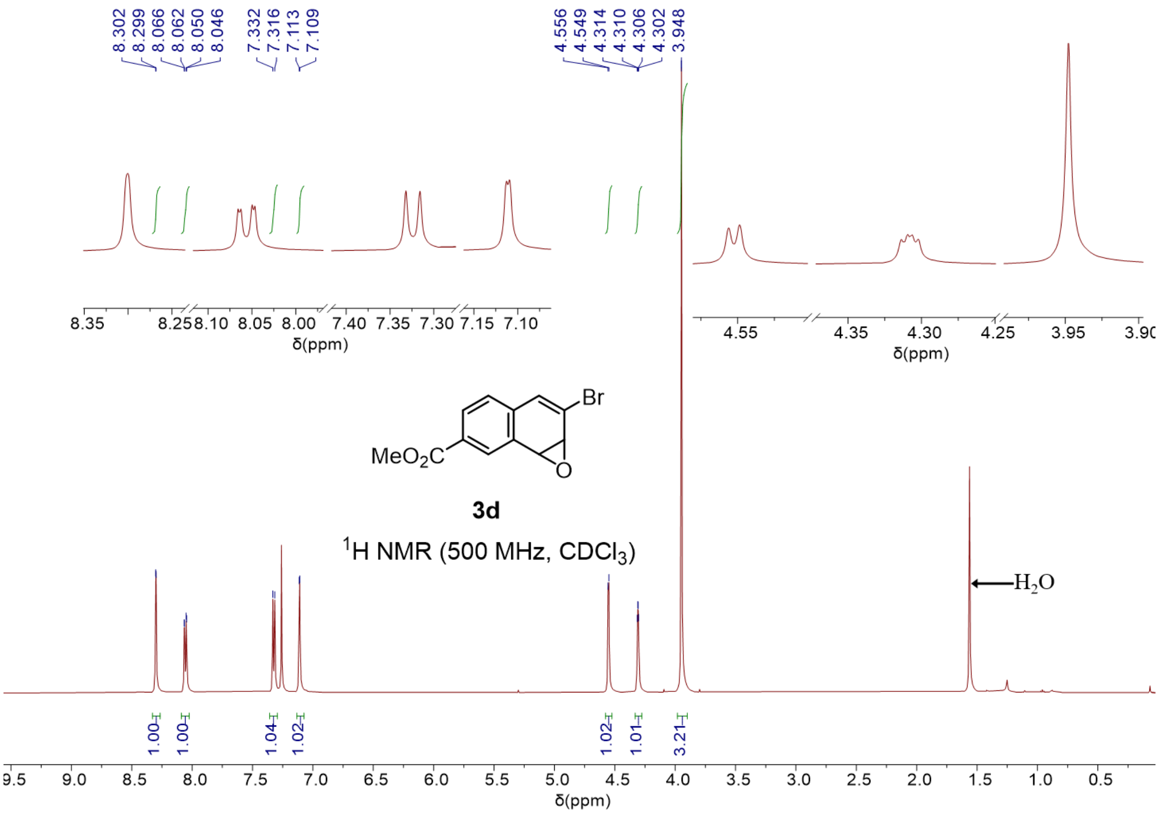

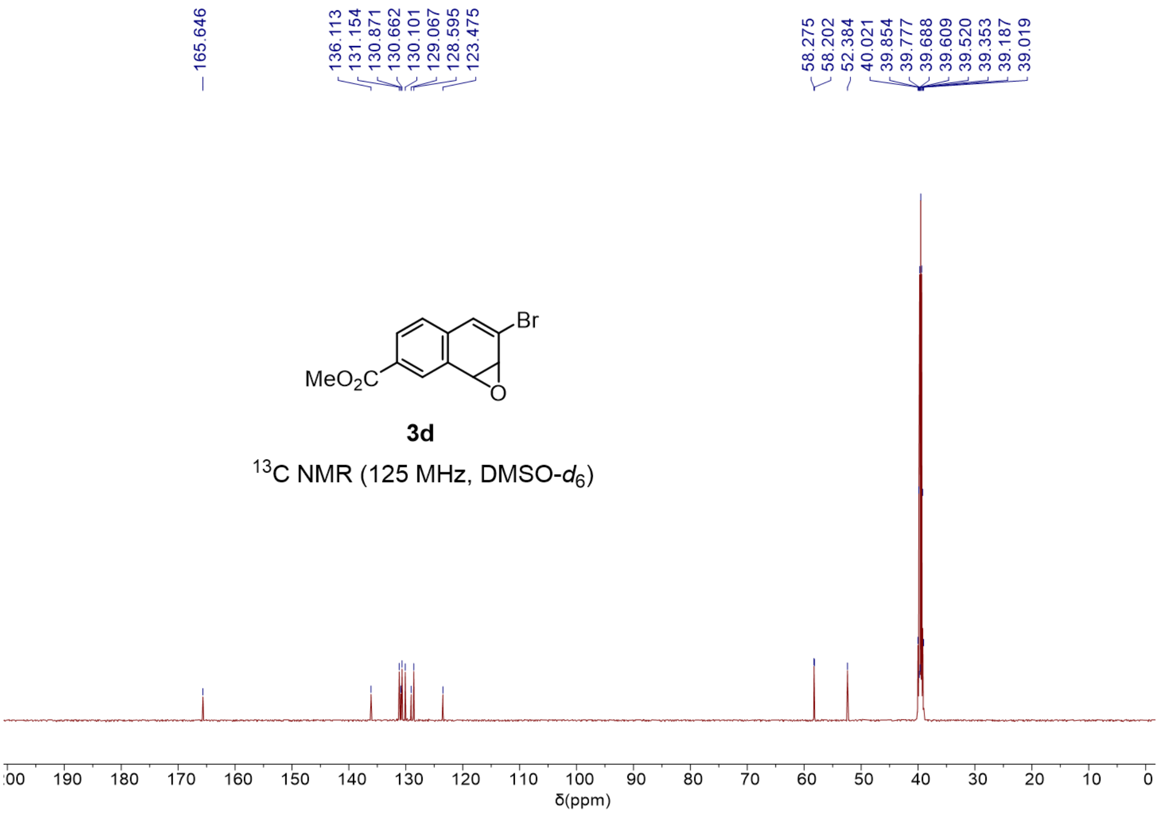

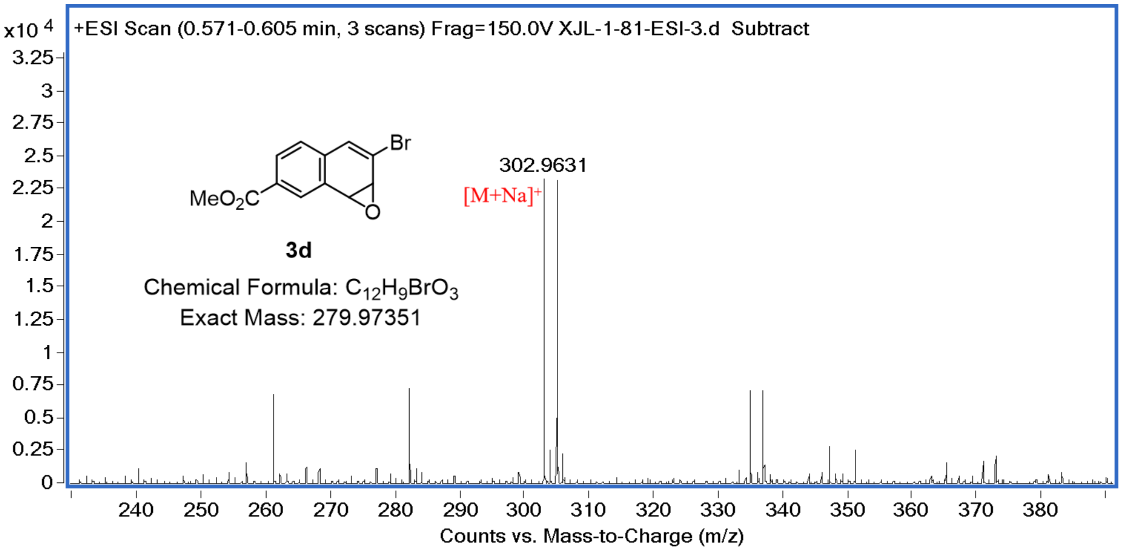

Formula (M)	Ion Formula	*m*/*z*	Calc *m*/*z*	Diff (ppm)	DBE
$C_{12}H_9BrO_3$	$C_{12}H_9BrNaO_3$	302.9631	302.9627	-1.33	8

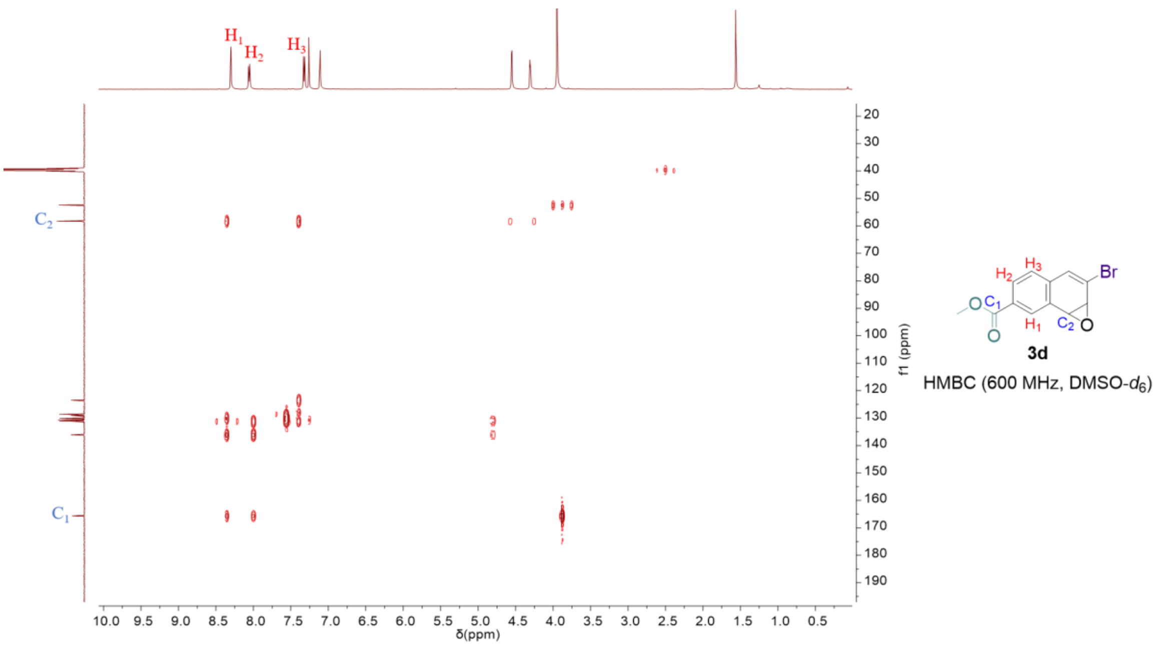

3d
HMBC (600 MHz, DMSO-d_6)

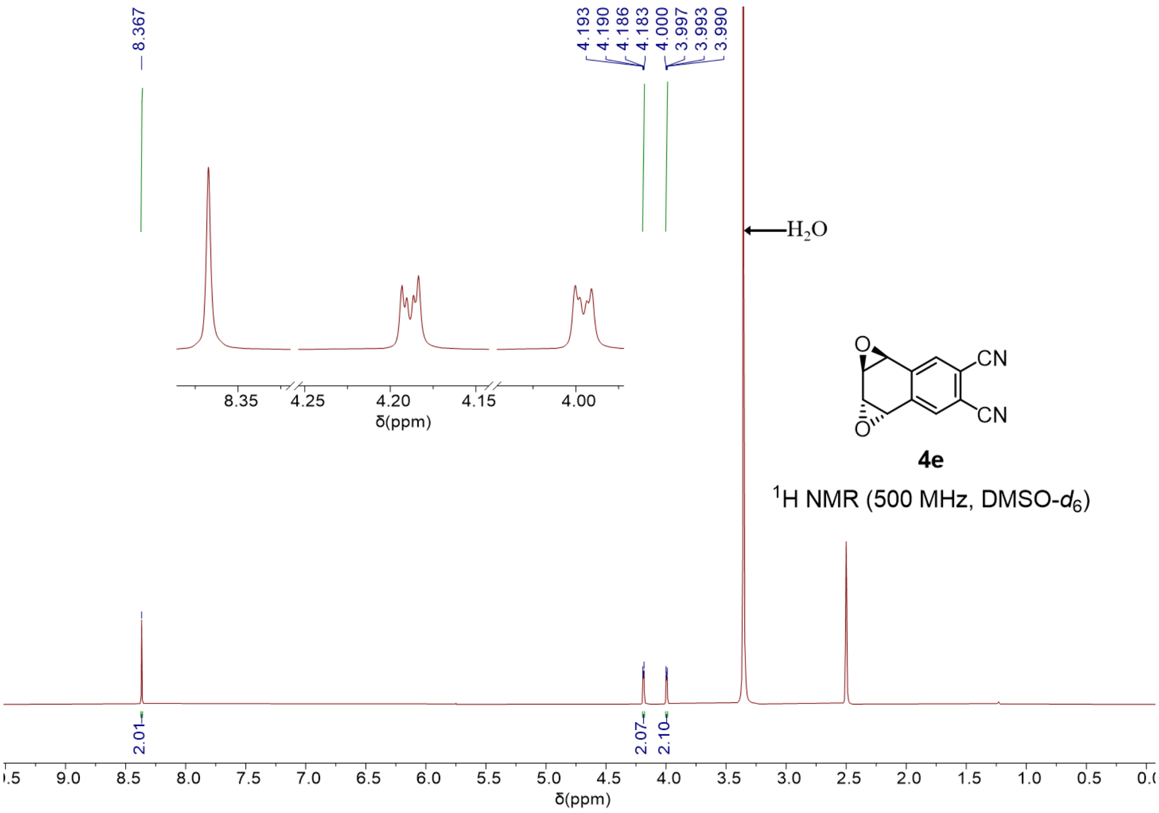

4e
^{1}H NMR (500 MHz, DMSO-d_6)

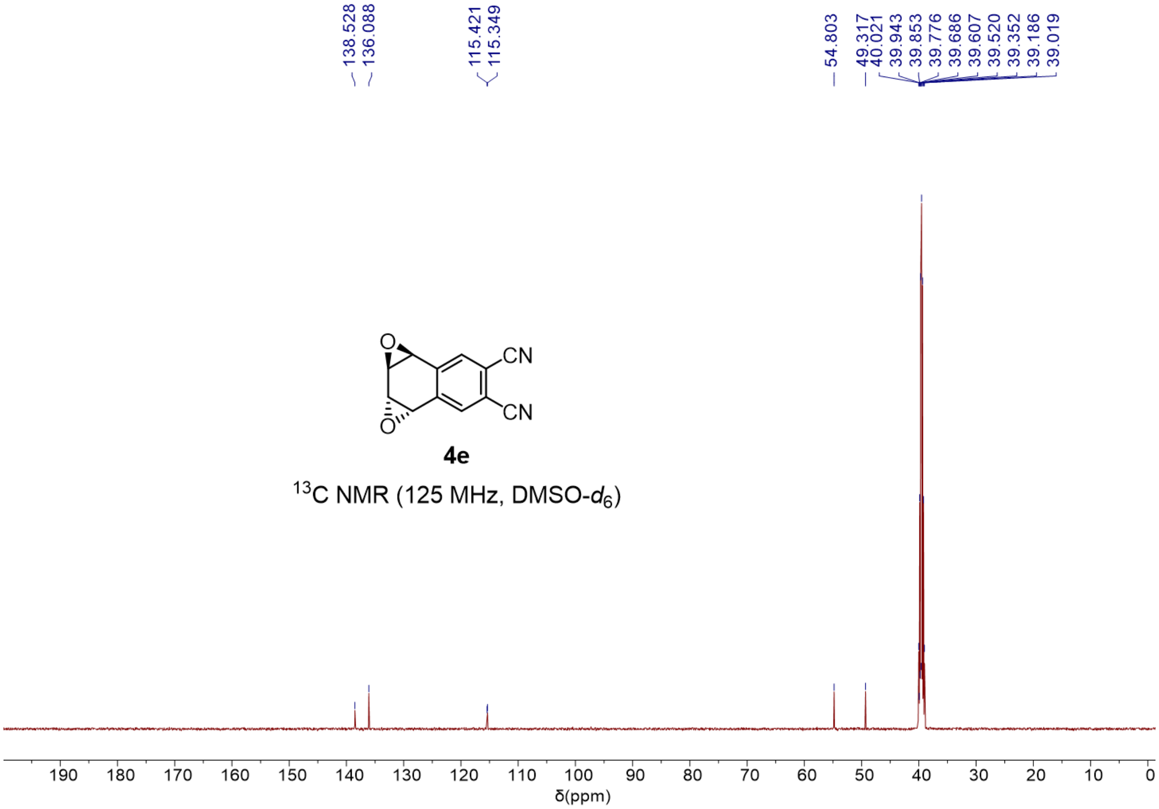

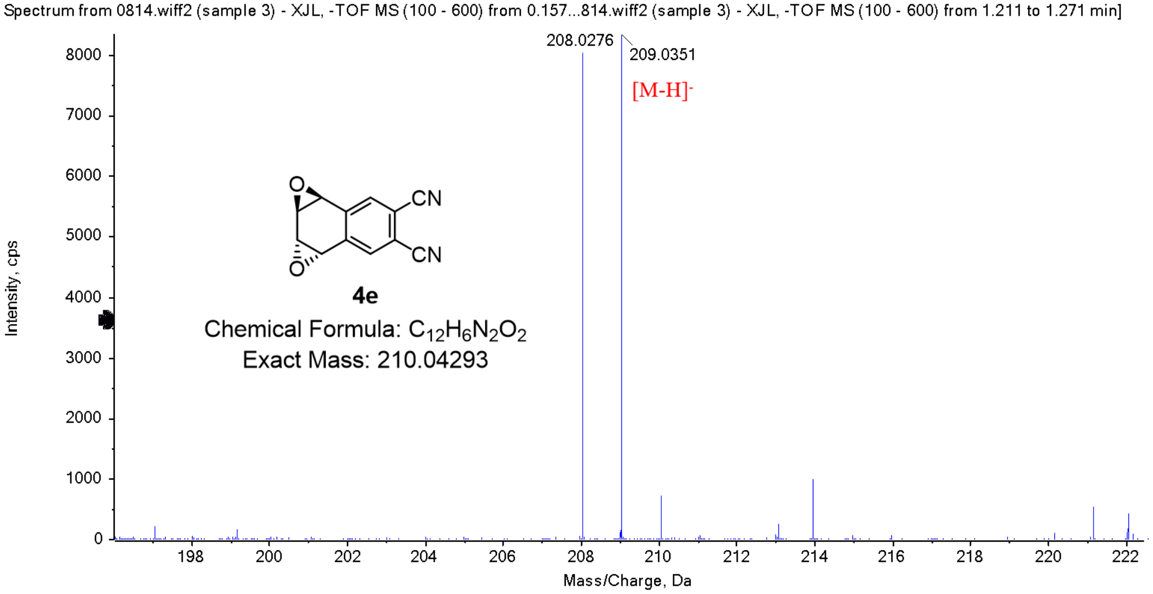

IonFormula	Calculated*m/z*	Error (ppm)	Error (mmu)	RDB
$C_{12}H_5N_2O_2$	209.03565	-2.9	-0.60	11.0

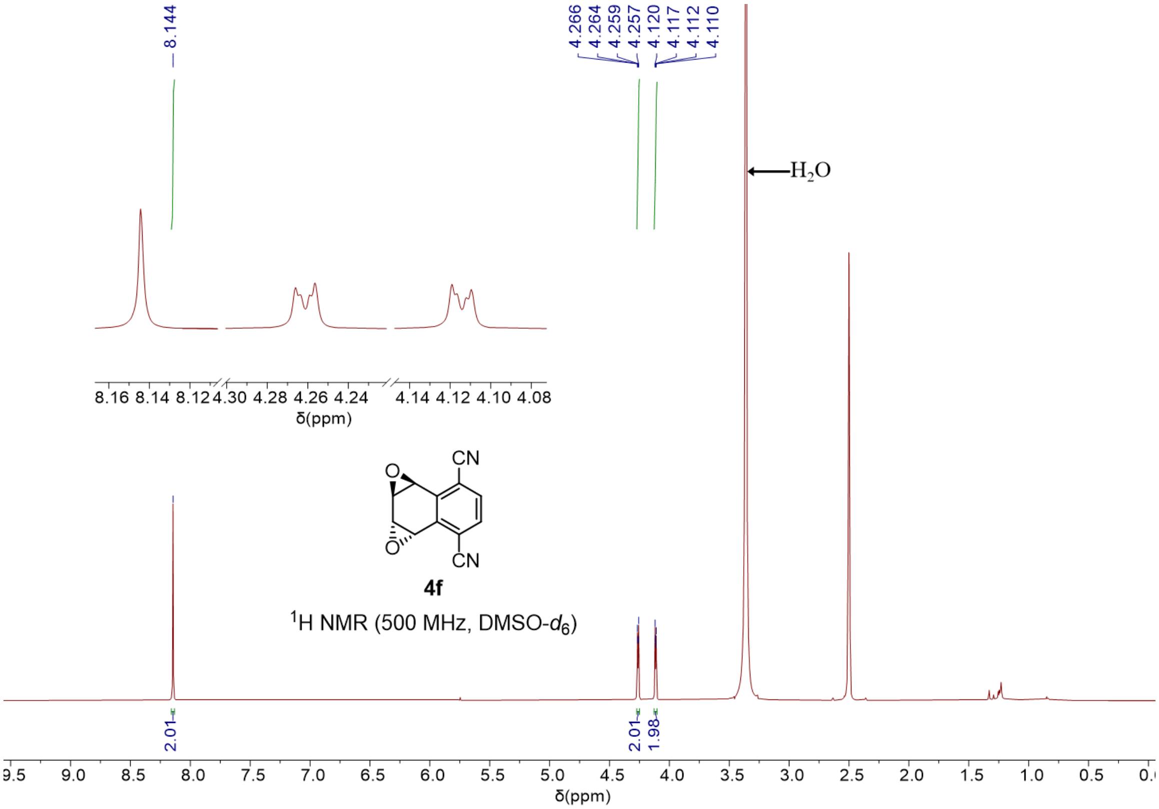

8.144
4.266
4.264
4.259
4.257
4.120
4.117
4.112
4.110
H2O
8.16 8.14 8.12 4.30 4.28 4.26 4.24 4.14 4.12 4.10 4.08
δ(ppm)
CN
O
O
CN
4f
1H NMR (500 MHz, DMSO-d6)
2.01
2.01
1.98
9.5 9.0 8.5 8.0 7.5 7.0 6.5 6.0 5.5 5.0 4.5 4.0 3.5 3.0 2.5 2.0 1.5 1.0 0.5
δ(ppm)

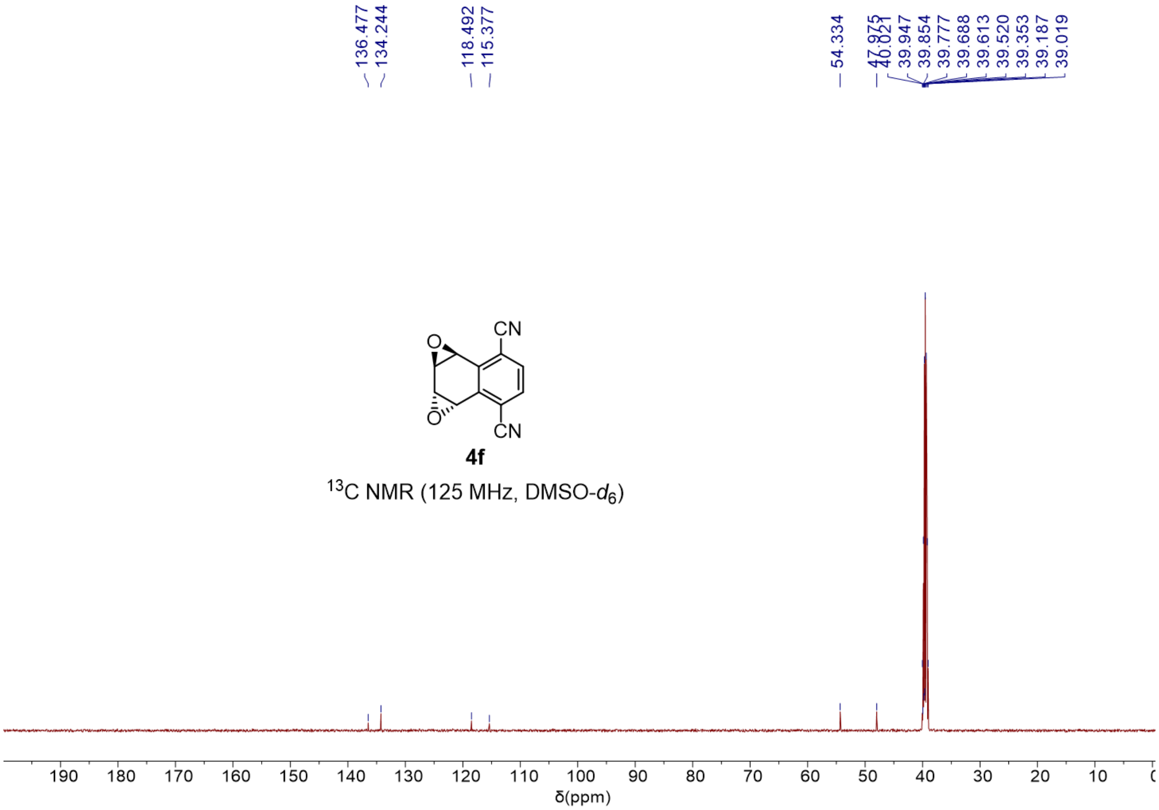

136.477
134.244
118.492
115.377
54.334
47.975
40.021
39.947
39.854
39.777
39.688
39.613
39.520
39.353
39.187
39.019
CN
O
O
CN
4f
13C NMR (125 MHz, DMSO-d6)
190 180 170 160 150 140 130 120 110 100 90 80 70 60 50 40 30 20 10
δ(ppm)

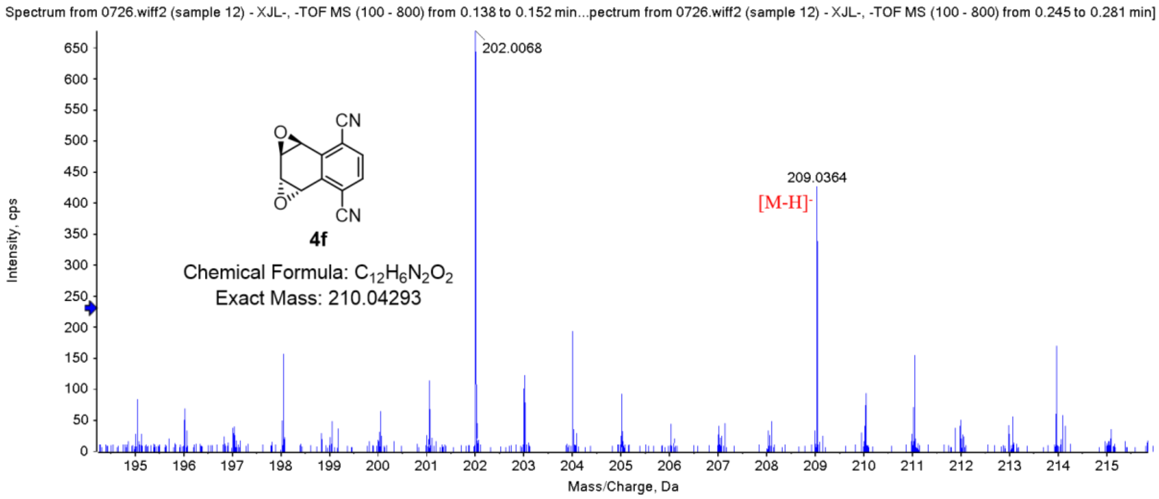

Ion Formula	Calculated *m/z*	Error ppm)	Error (mmu)	RDB
$C_{12}H_5N_2O_2$	209.03565	3.4	0.72	11.0

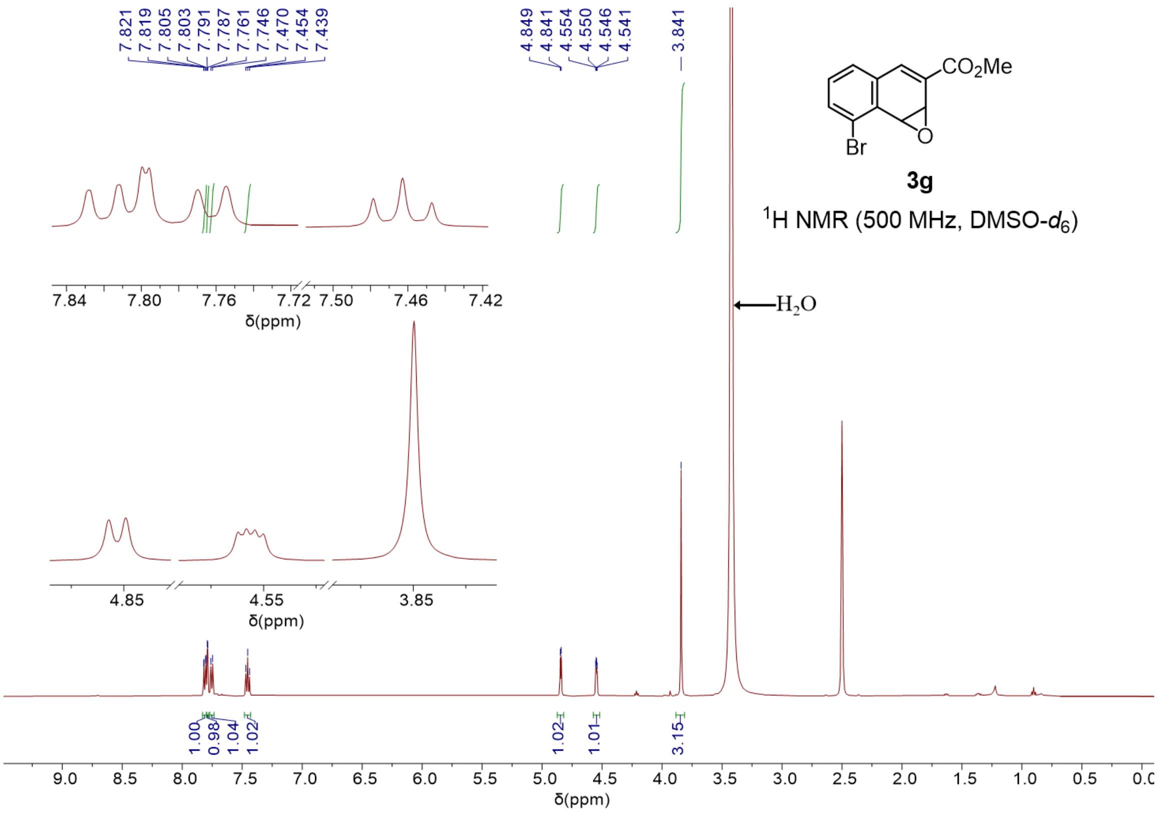

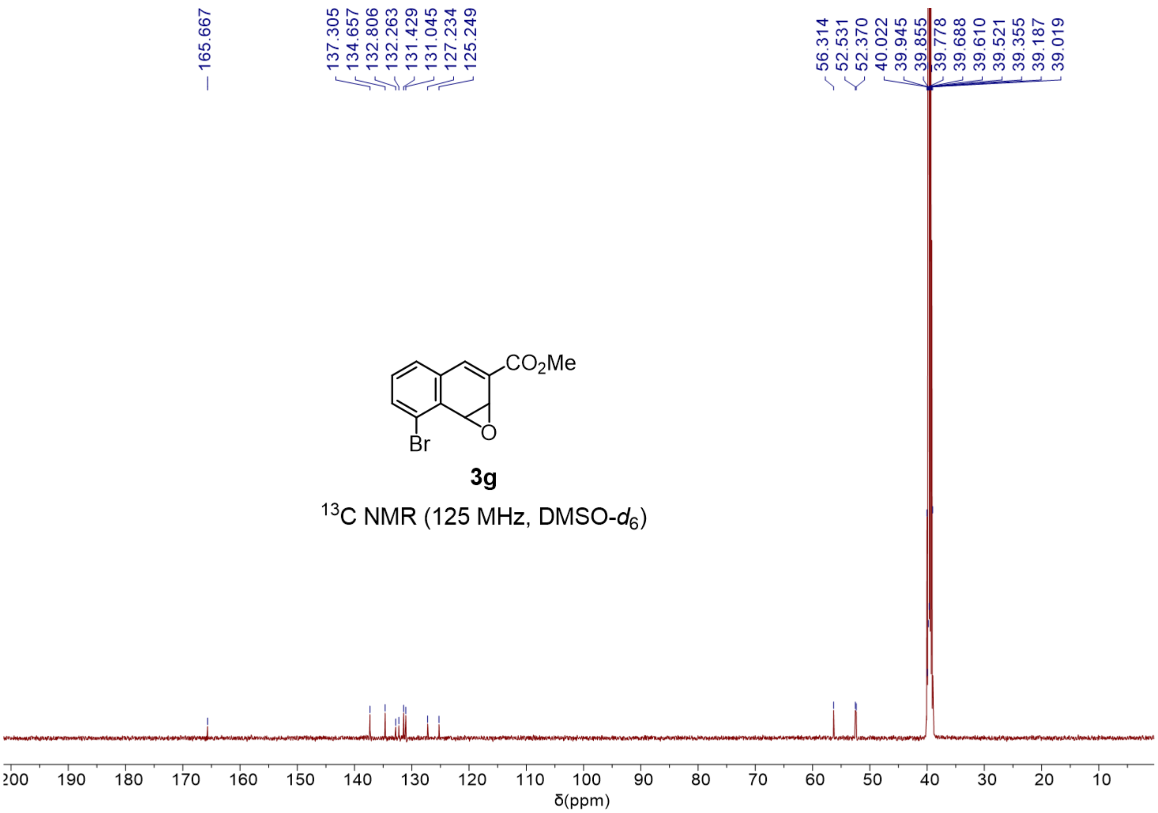

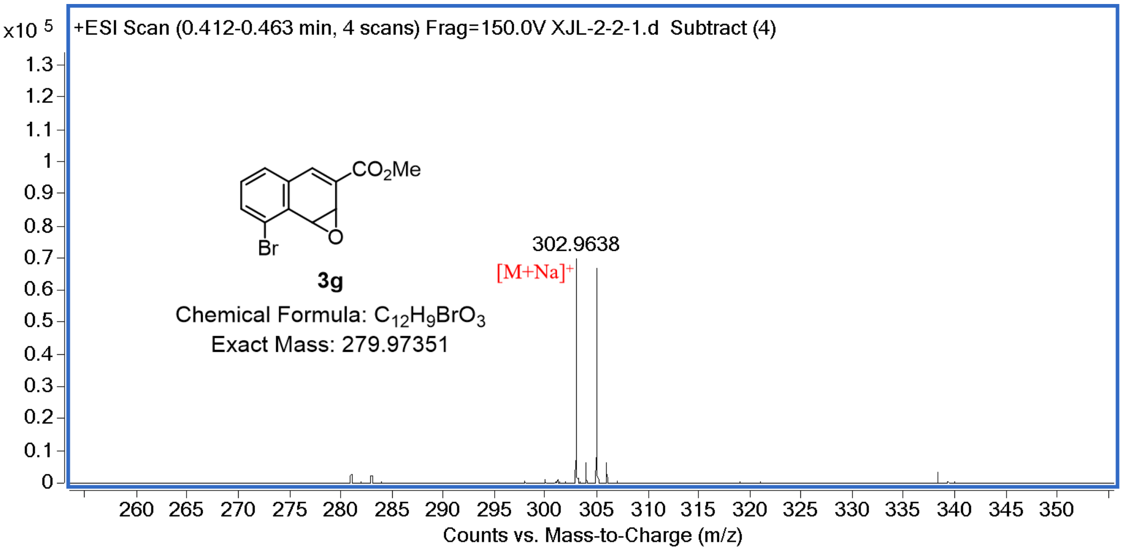

Formula (M)	Ion Formula	Calc *m/z*	Diff (ppm)	DBE
$C_{12}H_9BrO_3$	$C_{12}H_9BrNaO_3$	302.9627	-3.83	8

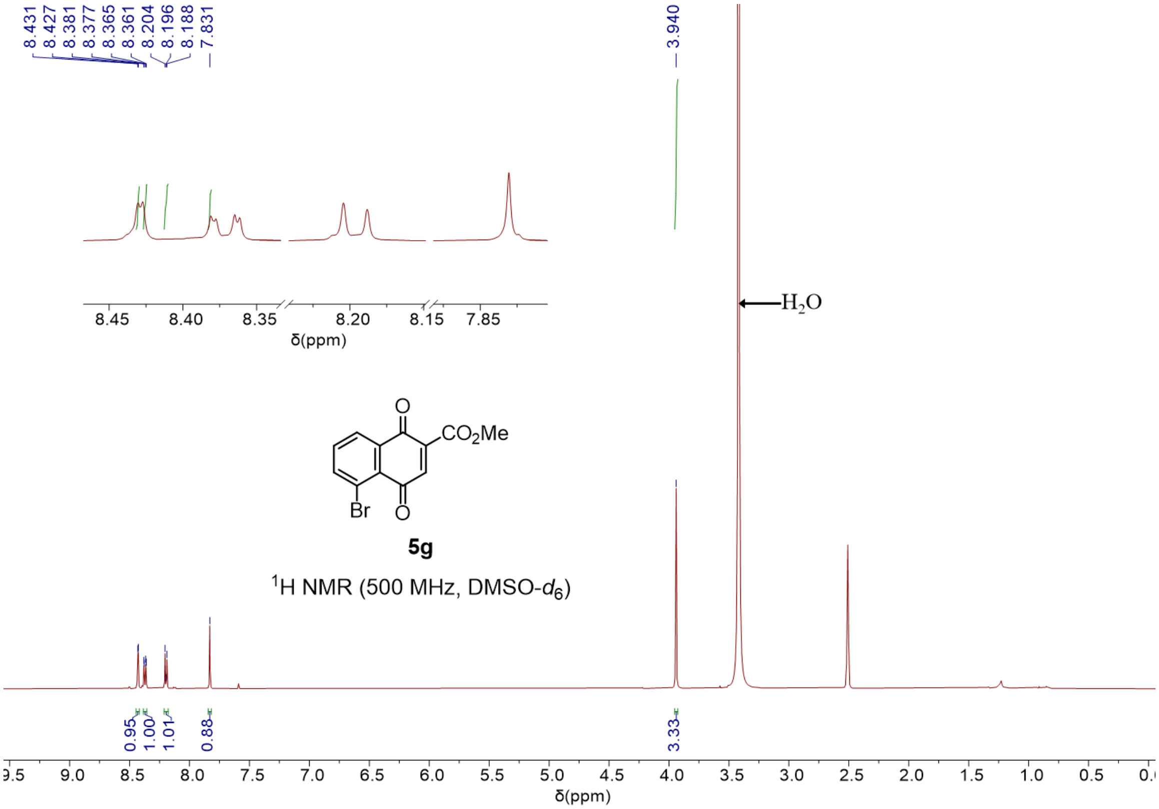
8.431
8.427
8.381
8.377
8.365
8.361
8.204
8.196
8.188
7.831
3.940
8.45
8.40
8.35
8.20
8.15
7.85
δ(ppm)
H2O
CO2Me
Br
O
O
5g
1H NMR (500 MHz, DMSO-d6)
0.95
1.00
1.01
0.88
3.33
9.0
8.5
8.0
7.5
7.0
6.5
6.0
5.5
5.0
4.5
4.0
3.5
3.0
2.5
2.0
1.5
1.0
0.5
δ(ppm)

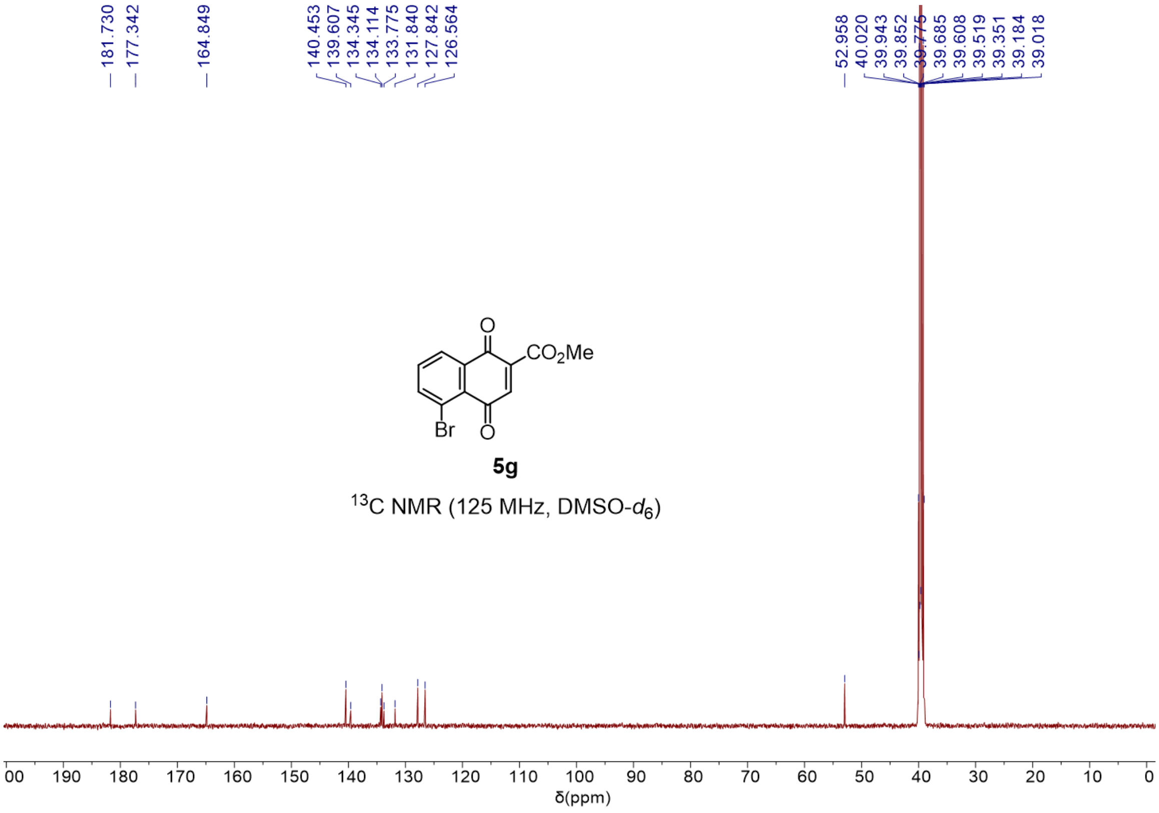
181.730
177.342
164.849
140.453
139.607
134.345
134.114
133.775
131.840
127.842
126.564
52.958
40.020
39.943
39.852
39.775
39.685
39.608
39.519
39.351
39.184
39.018
CO2Me
Br
O
O
5g
13C NMR (125 MHz, DMSO-d6)
190
180
170
160
150
140
130
120
110
100
90
80
70
60
50
40
30
20
10
0
δ(ppm)

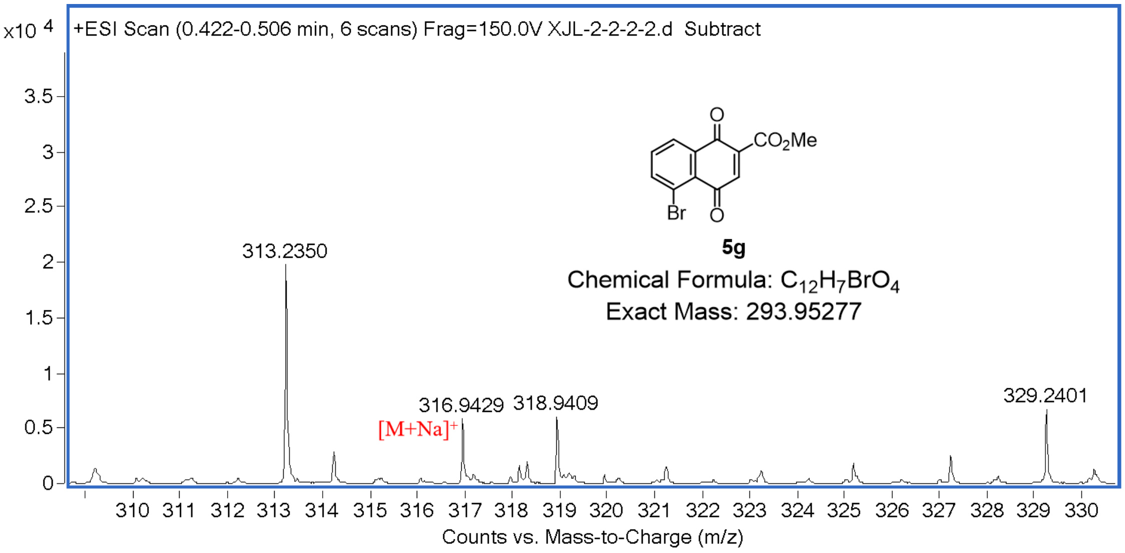

Formula (M)	Ion Formula	Calc *m/z*	Diff (ppm)	DBE
$C_{12}H_7BrO_4$	$C_{12}H_7BrNaO_4$	316.942	-3.09	9

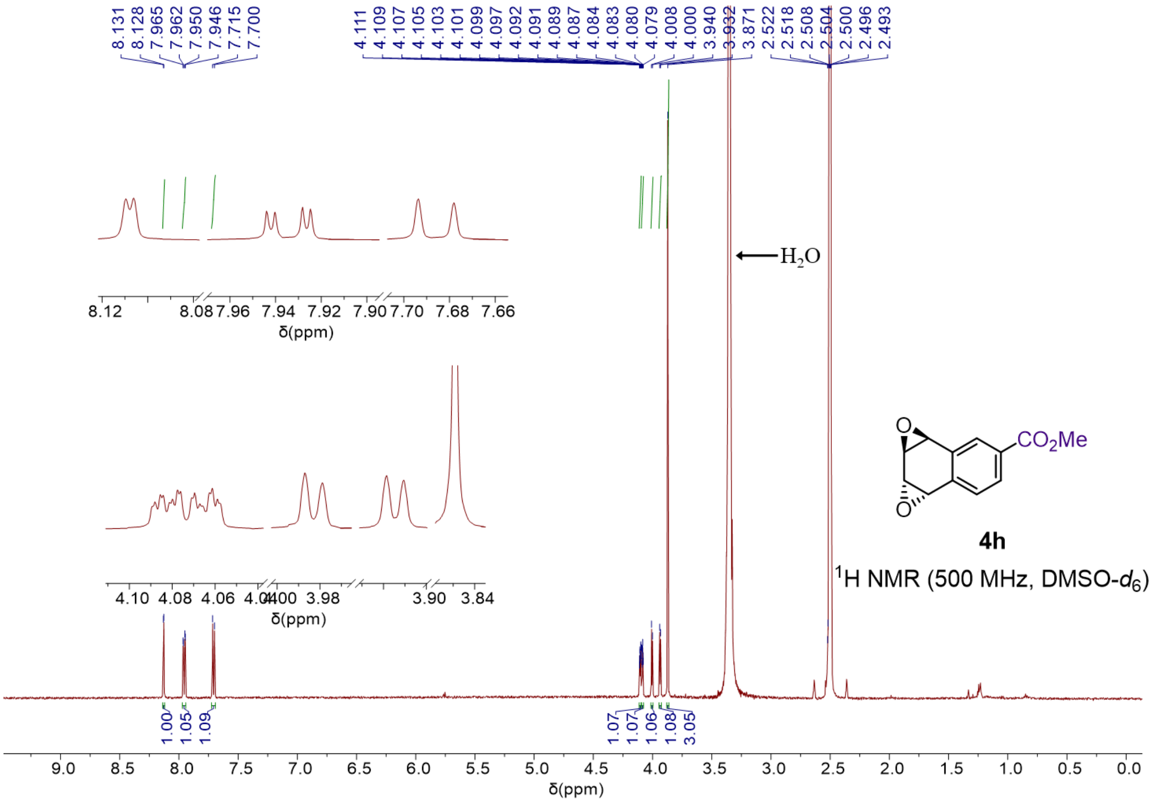

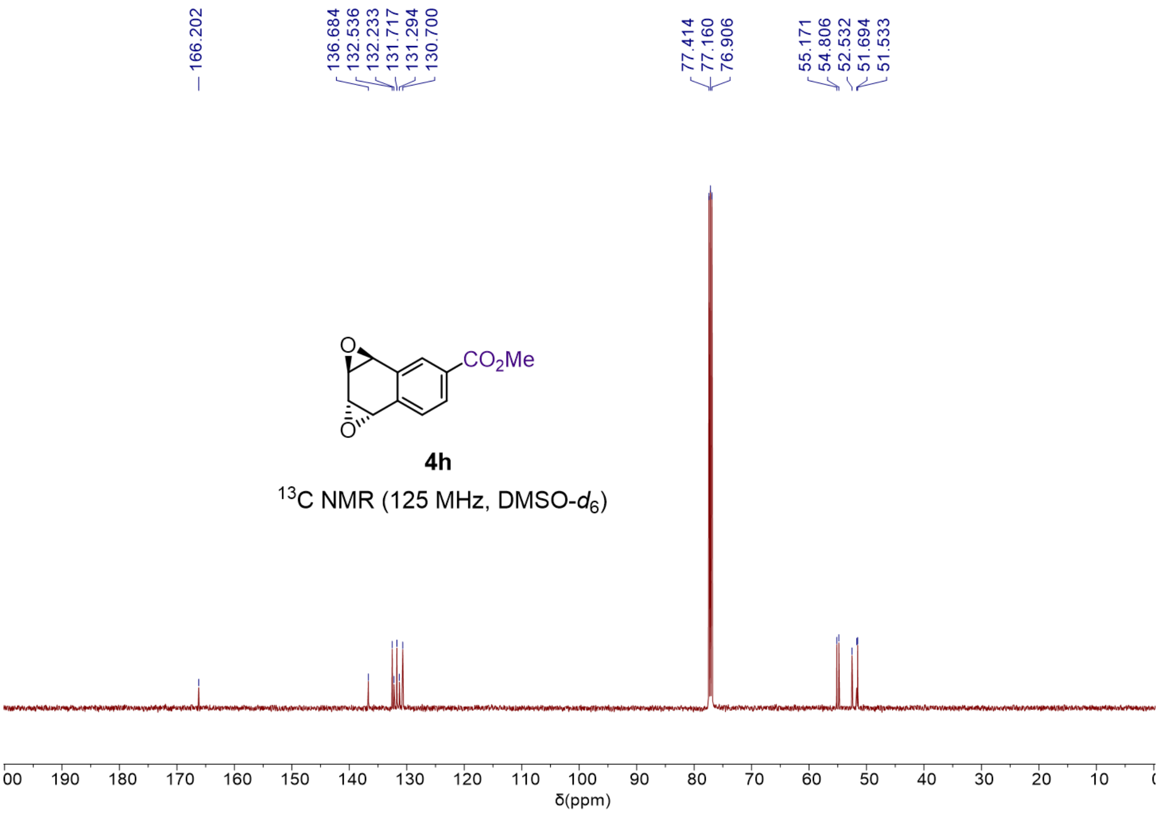

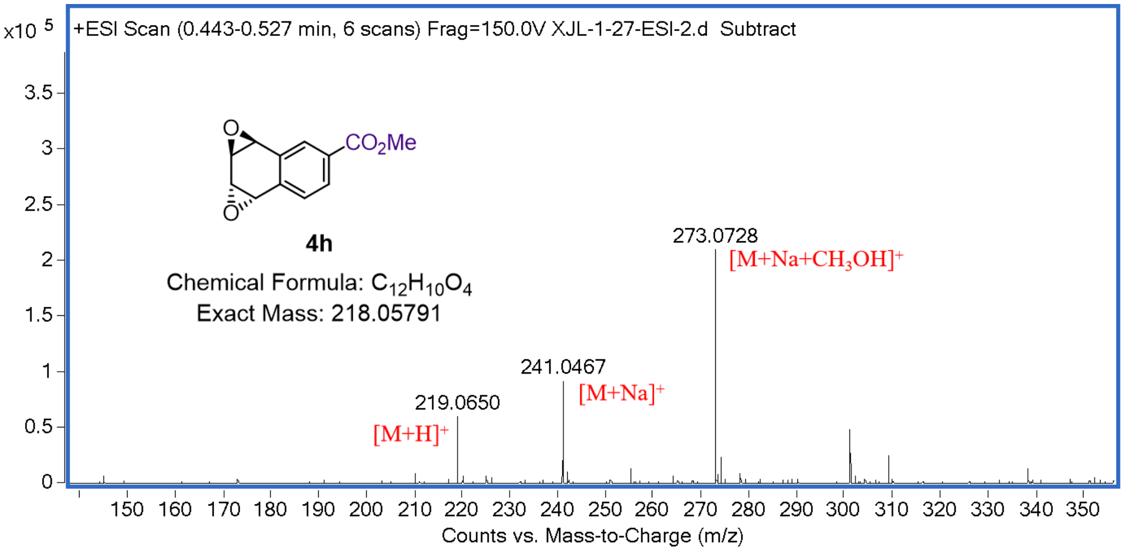

Formula (M)	Ion Formula	Calc *m/z*	Diff (ppm)	DBE
$C_{12}H_{10}O_4$	$C_{12}H_{11}O_4$	219.0652	0.85	8

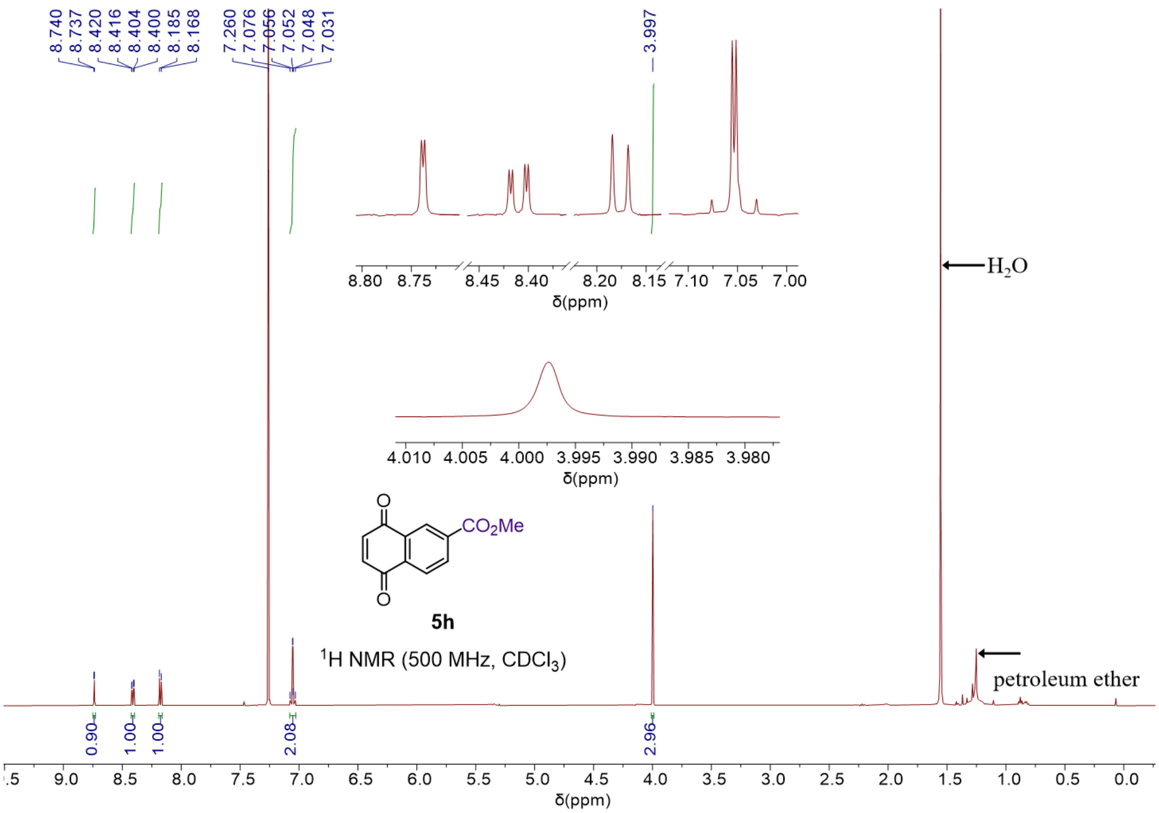

H2O
petroleum ether
CO2Me
5h
1H NMR (500 MHz, CDCl3)
δ(ppm)

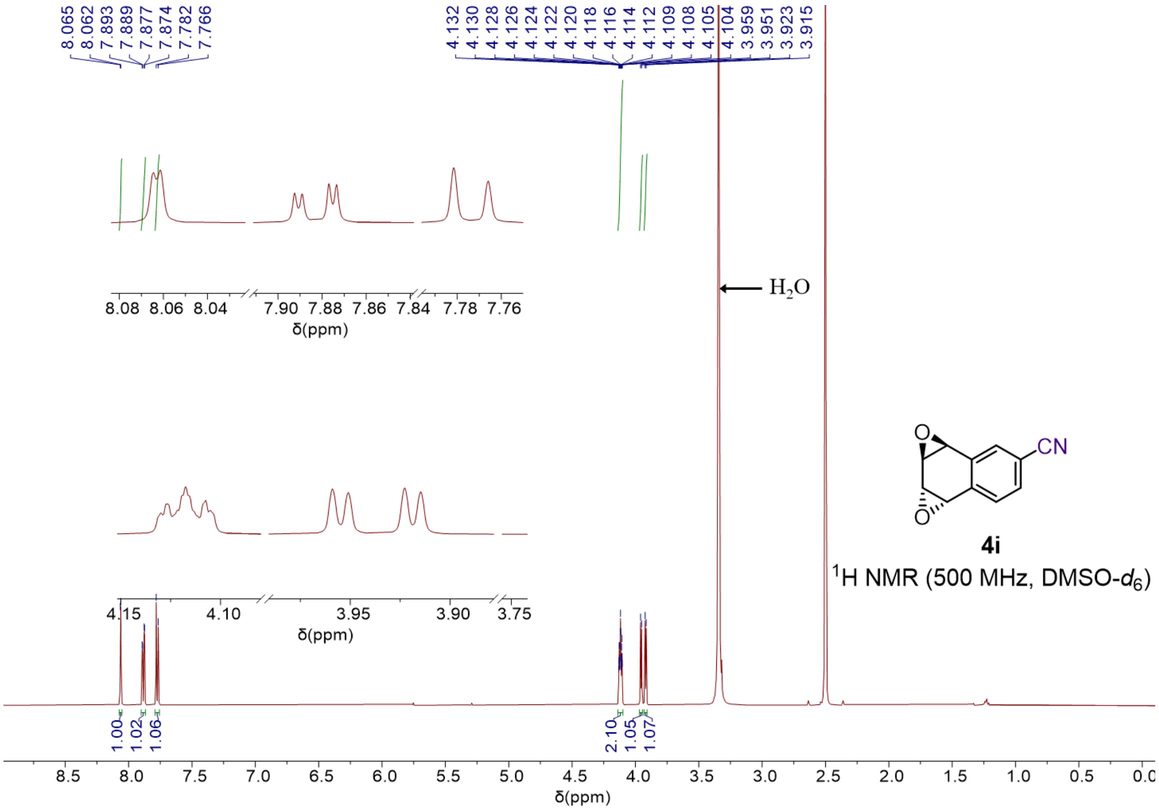

H2O
CN
4i
1H NMR (500 MHz, DMSO-d6)
δ(ppm)

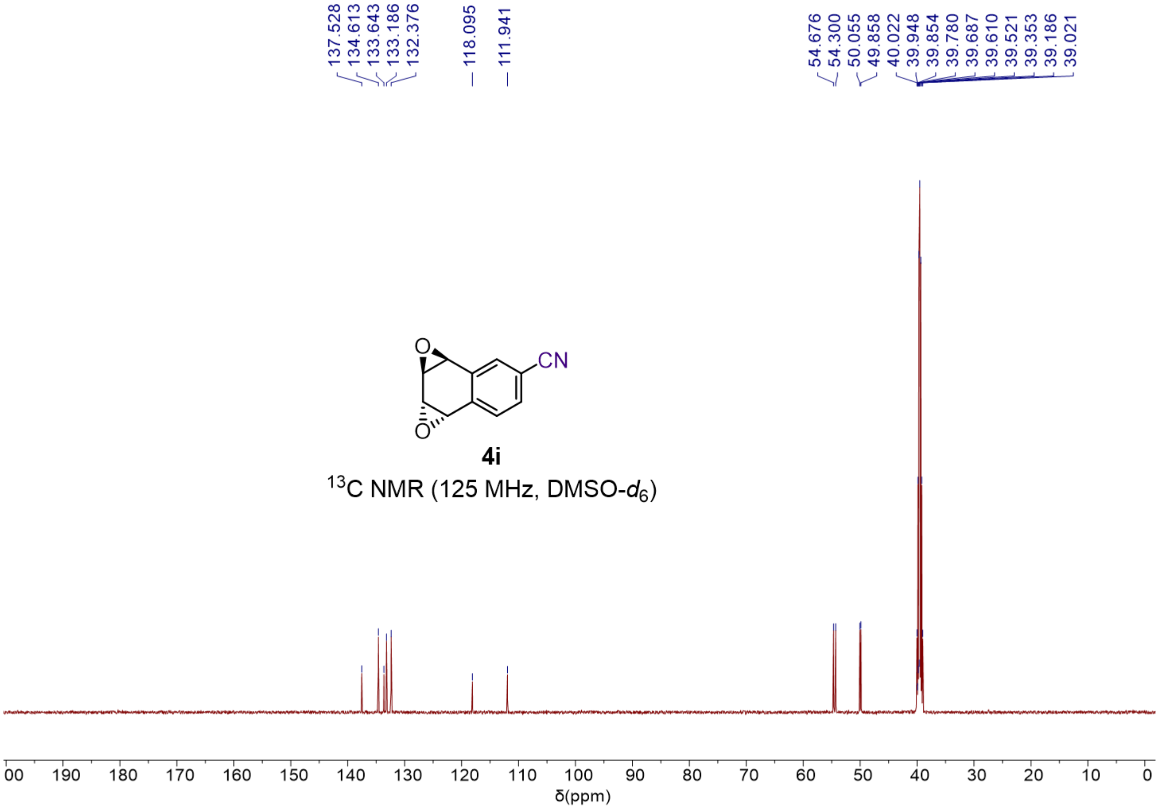

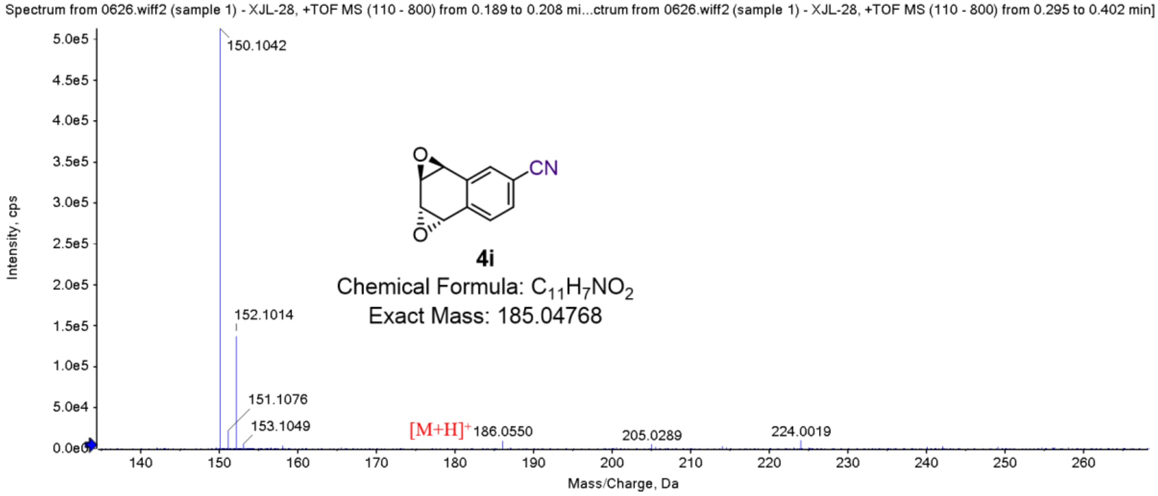

Ion Formula	Calculated *m/z*	Error (ppm)	Error (mmu)	RDB
$C_{11}H_8NO_2$	186.05495	0.1	0.02	9.0

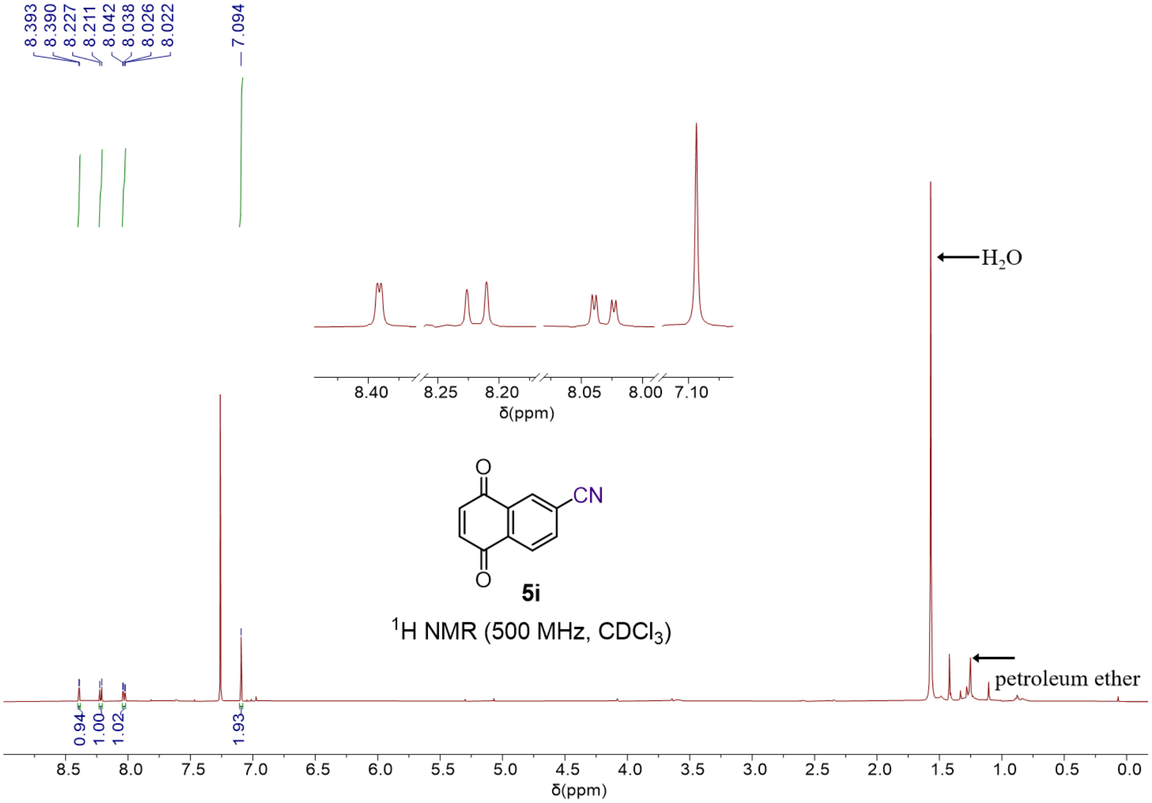
8.393
8.390
8.227
8.211
8.042
8.038
8.026
8.022
7.094
H2O
5i
1H NMR (500 MHz, CDCl3)
petroleum ether
0.94
1.00
1.02
1.93
δ(ppm)

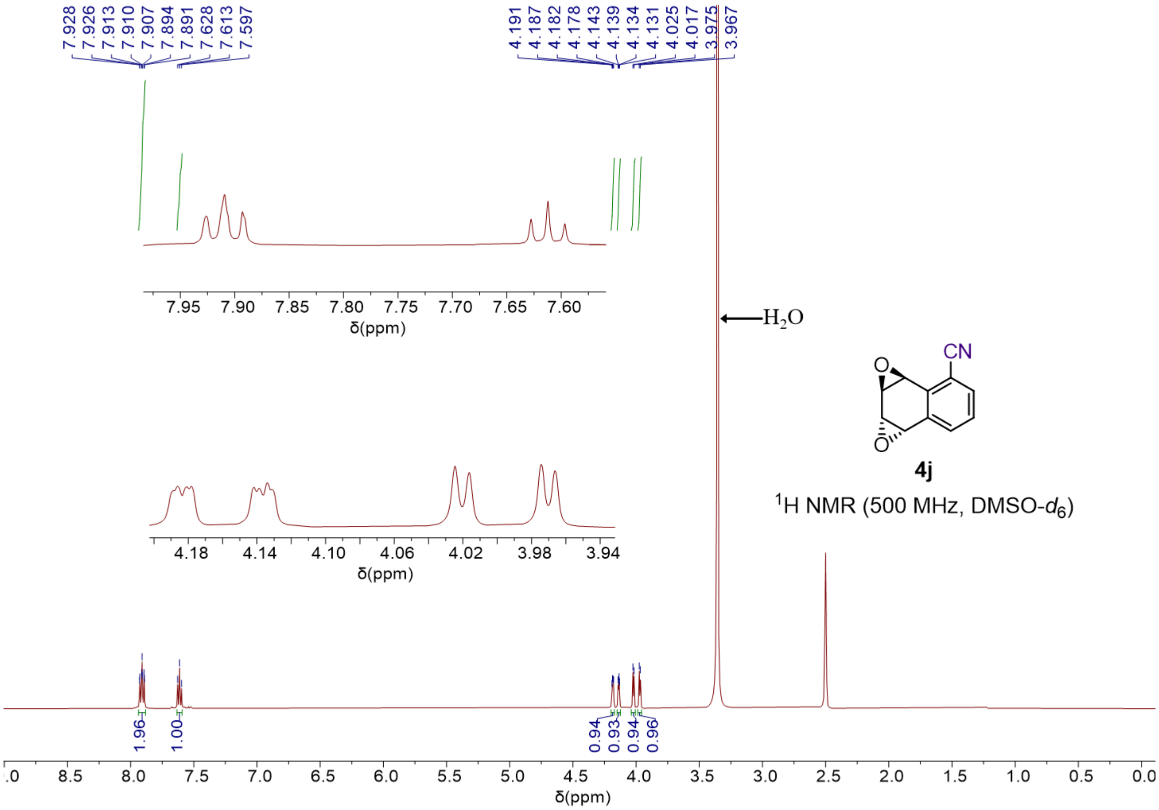
7.928
7.926
7.913
7.910
7.907
7.894
7.891
7.628
7.613
7.597
4.191
4.187
4.182
4.178
4.143
4.139
4.134
4.131
4.025
4.017
3.975
3.967
H2O
CN
4j
1H NMR (500 MHz, DMSO-d6)
1.96
1.00
0.94
0.93
0.94
0.96
δ(ppm)

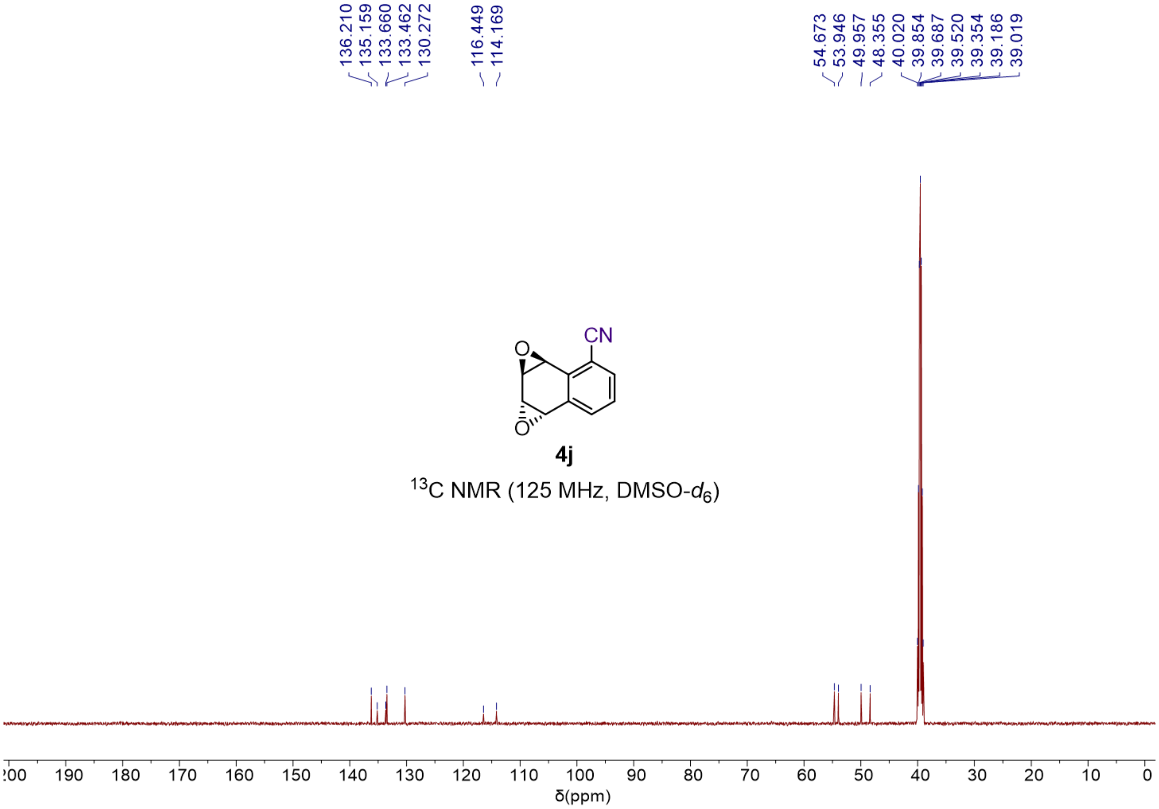

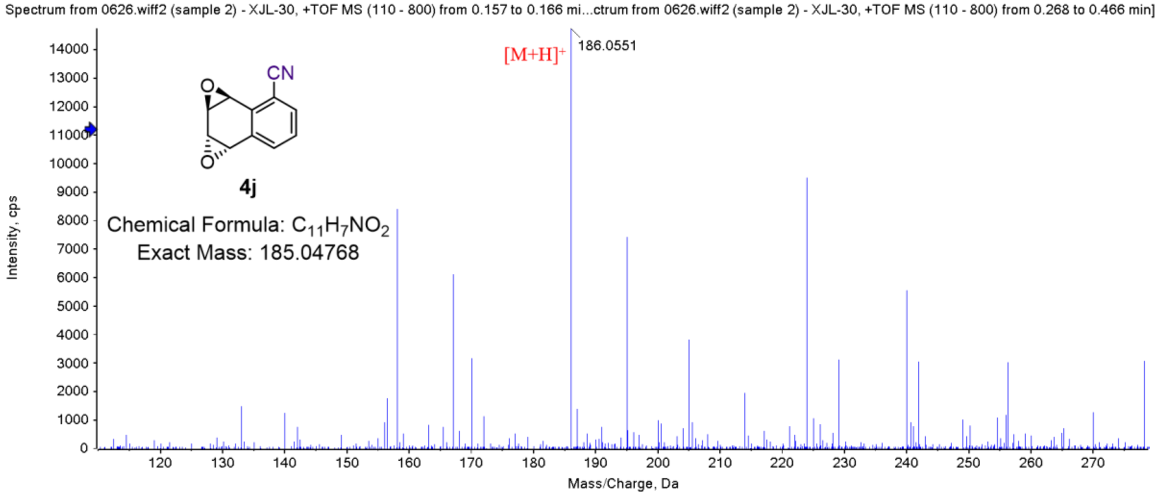

Ion Formula	Calculated *m/z*	Error (ppm)	Error (mmu)	RDB
$C_{11}H_8NO_2$	186.05495	0.7	0.13	9.0

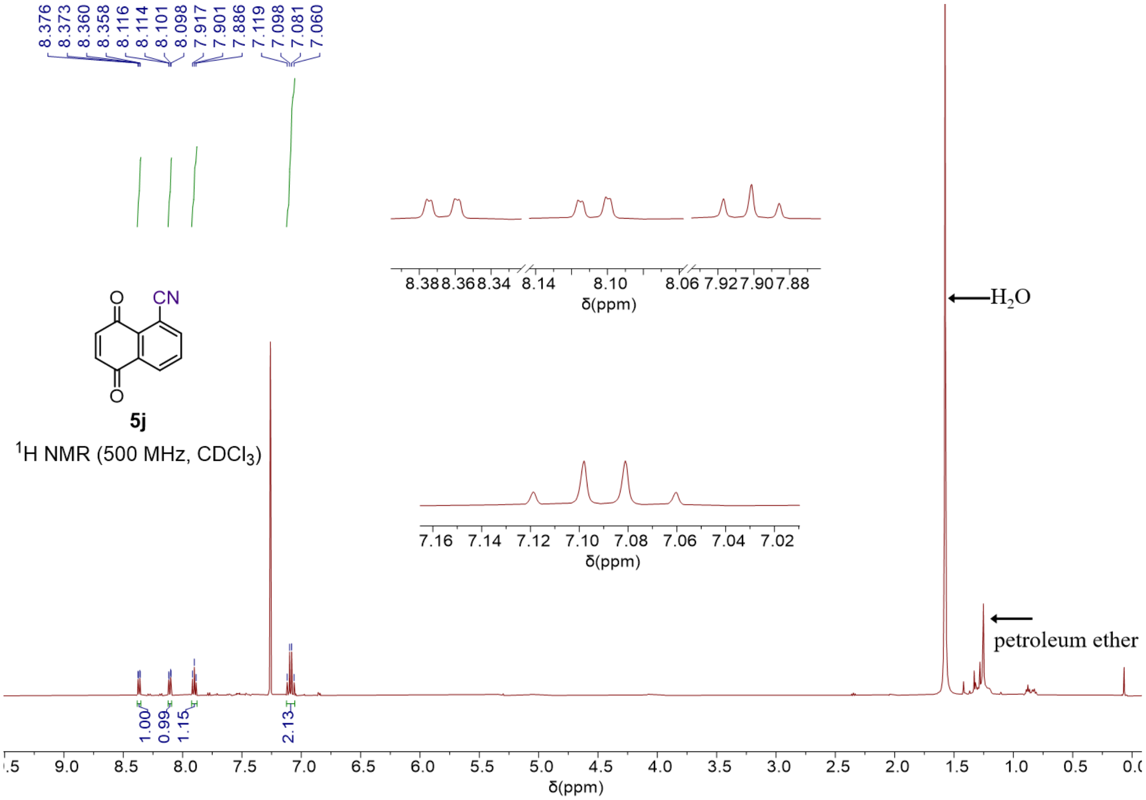
8.376
8.373
8.360
8.358
8.116
8.114
8.101
8.098
7.917
7.901
7.886
7.119
7.098
7.081
7.060
CN
O
O
5j
1H NMR (500 MHz, CDCl3)
H2O
petroleum ether
1.00
0.99
1.15
2.13
δ(ppm)

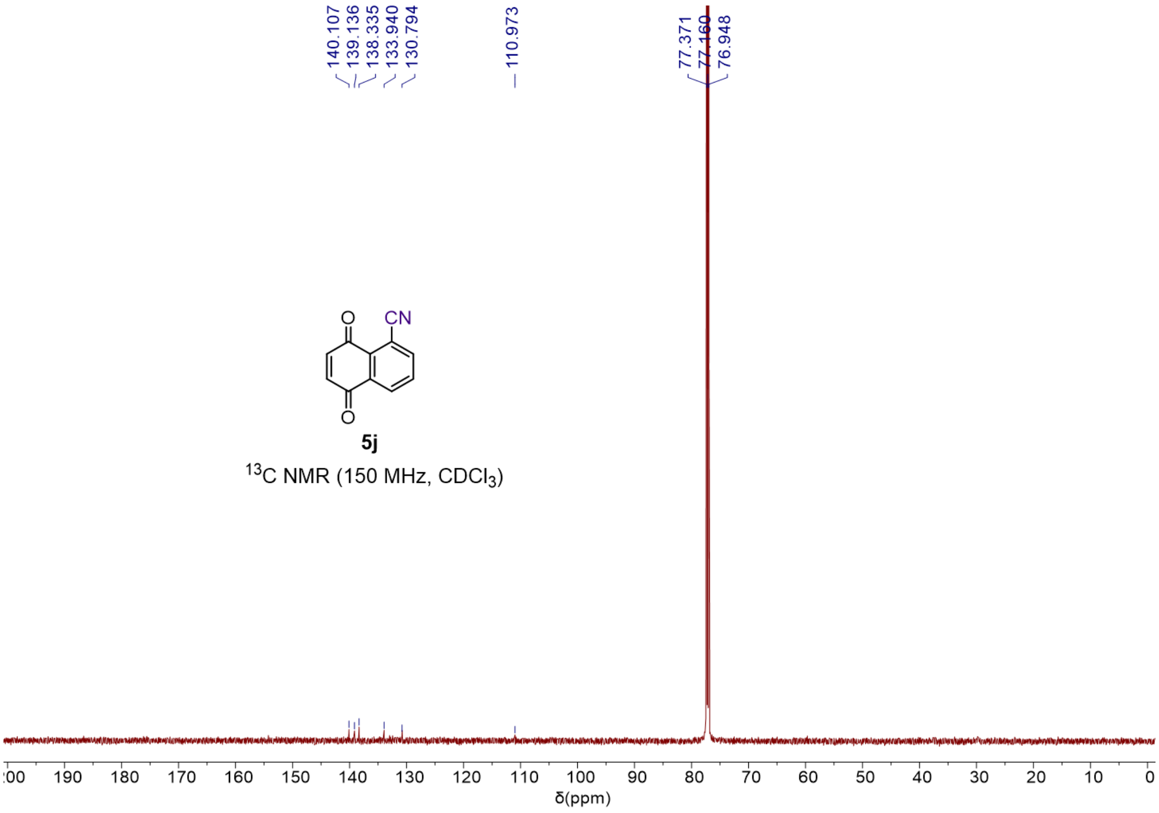
140.107
139.136
138.335
133.940
130.794
110.973
77.371
77.160
76.948
CN
O
O
5j
13C NMR (150 MHz, CDCl3)
δ(ppm)

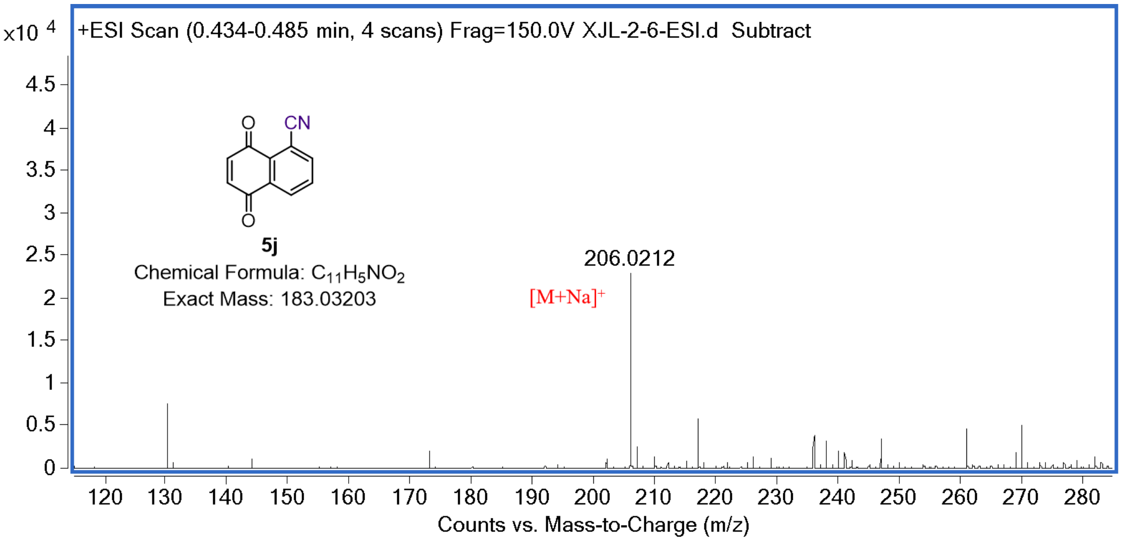

Formula (M)	Ion Formula	Calc *m/z*	Diff (ppm)	DBE
$C_{11}H_5NO_2$	$C_{11}H_5NNaO_2$	206.0212	0.82	10

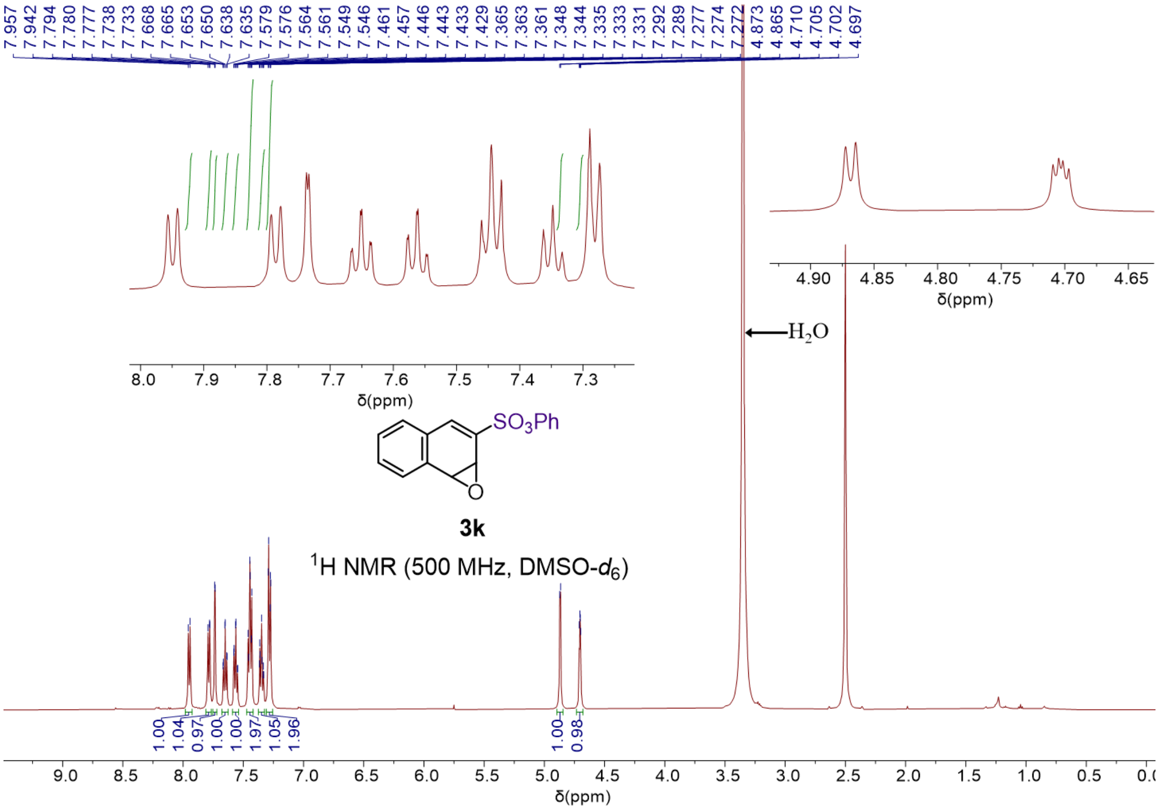

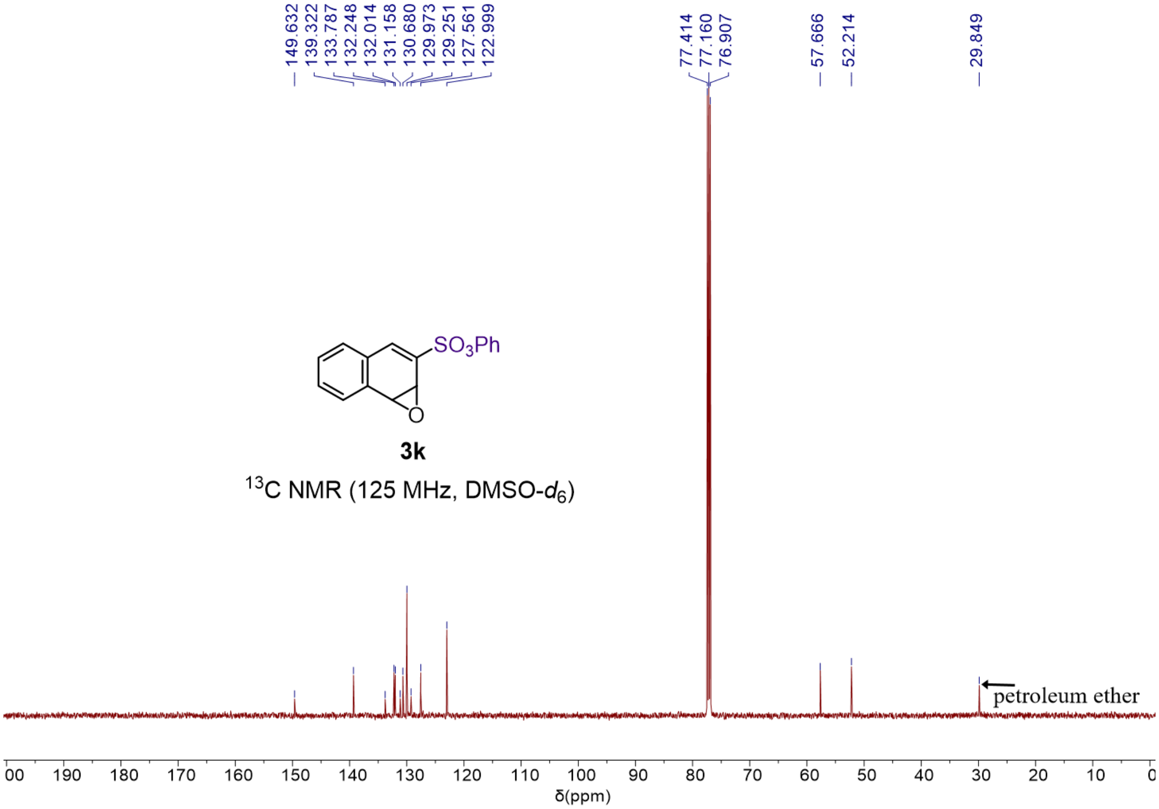

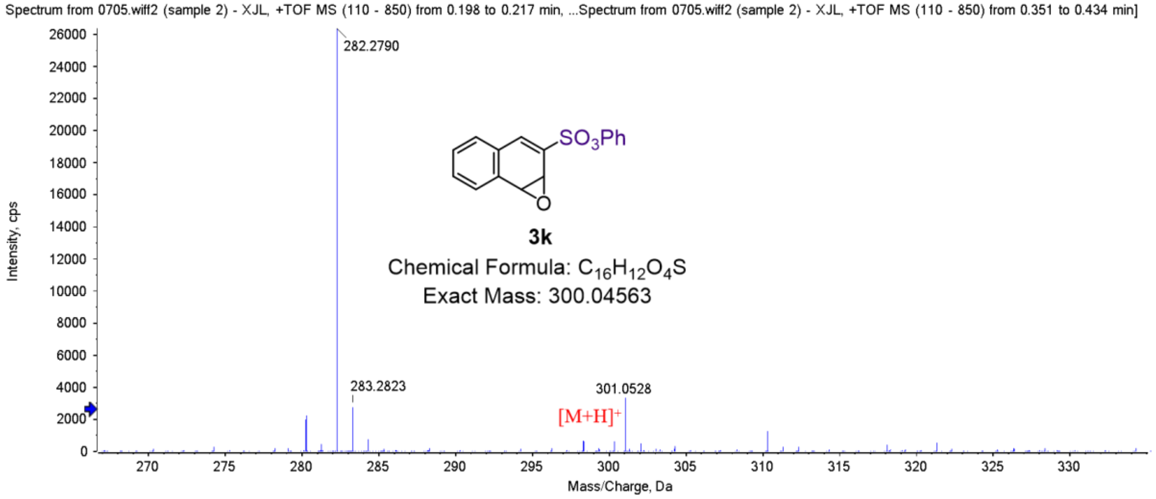

Ion Formula	Calculated *m/z*	Error (ppm)	Error (mmu)	RDB
$C_{16}H_{13}O_4S$	301.05291	-0.2	-0.07	11.0

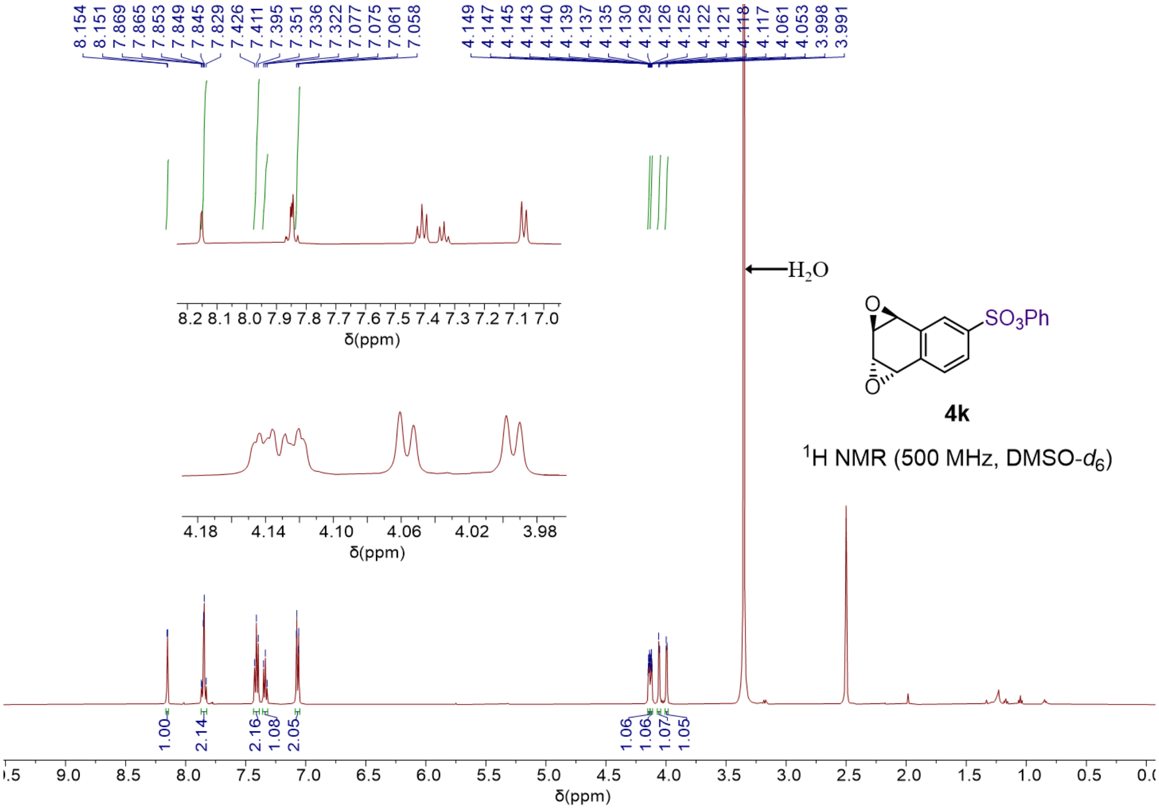

8.154
8.151
7.869
7.865
7.853
7.849
7.845
7.829
7.426
7.411
7.395
7.351
7.336
7.322
7.077
7.075
7.061
7.058
4.149
4.147
4.145
4.143
4.140
4.139
4.137
4.135
4.130
4.129
4.126
4.125
4.122
4.121
4.118
4.117
4.061
4.053
3.998
3.991
H2O
SO3Ph
4k
1H NMR (500 MHz, DMSO-d6)
δ(ppm)
1.00
2.14
2.16
1.08
2.05
1.06
1.06
1.07
1.05

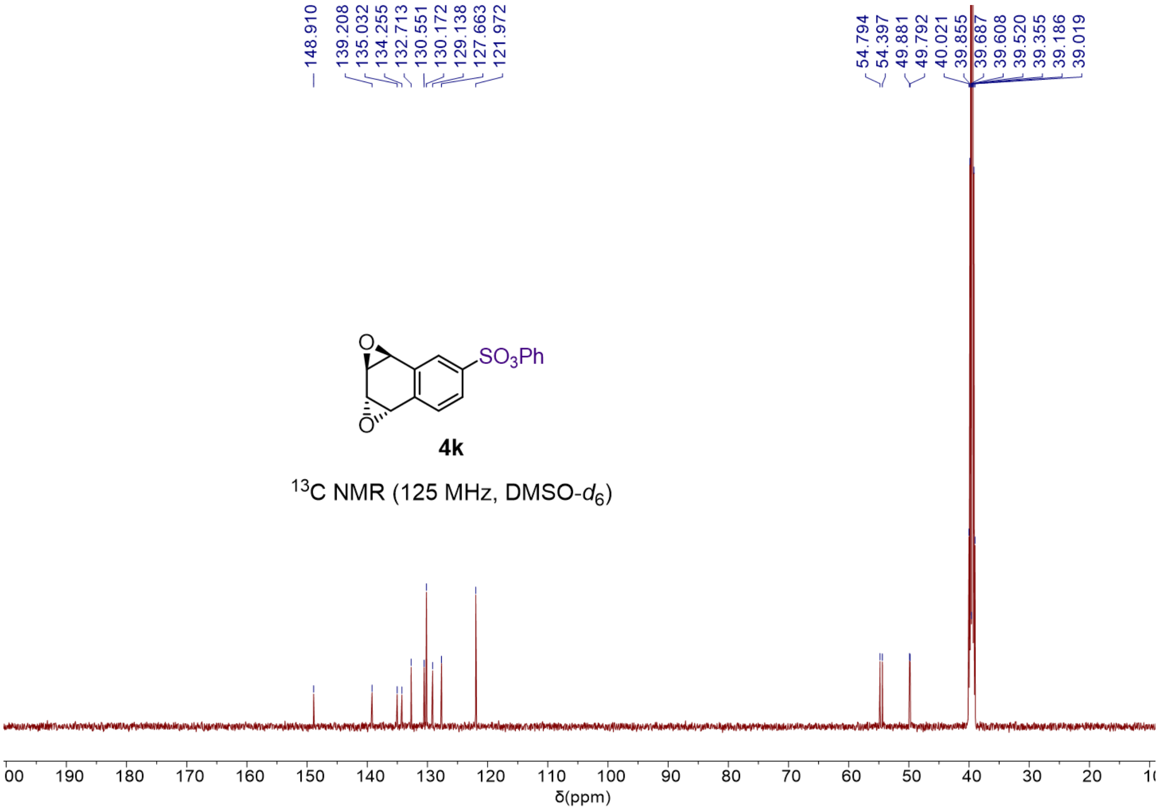

148.910
139.208
135.032
134.255
132.713
130.551
130.172
129.138
127.663
121.972
54.794
54.397
49.881
49.792
40.021
39.855
39.687
39.608
39.520
39.355
39.186
39.019
SO3Ph
4k
13C NMR (125 MHz, DMSO-d6)
δ(ppm)

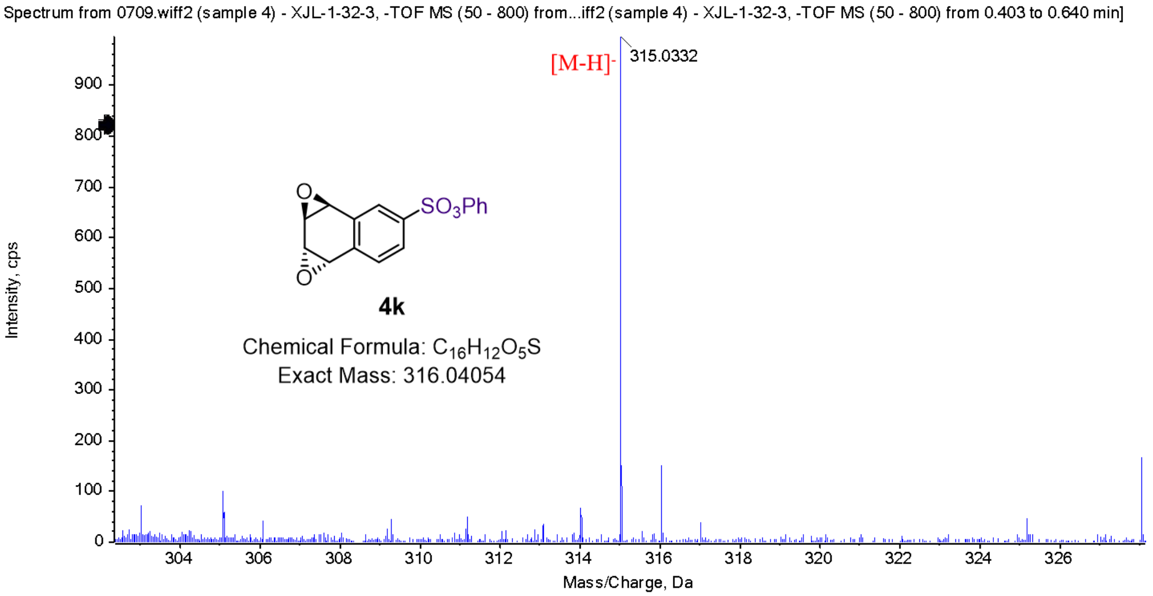

Ion Formula	Calculated *m/z*	Error (ppm)	Error (mmu)	RDB
$C_{16}H_{11}O_5S$	315.03327	-0.2	-0.07	11.0

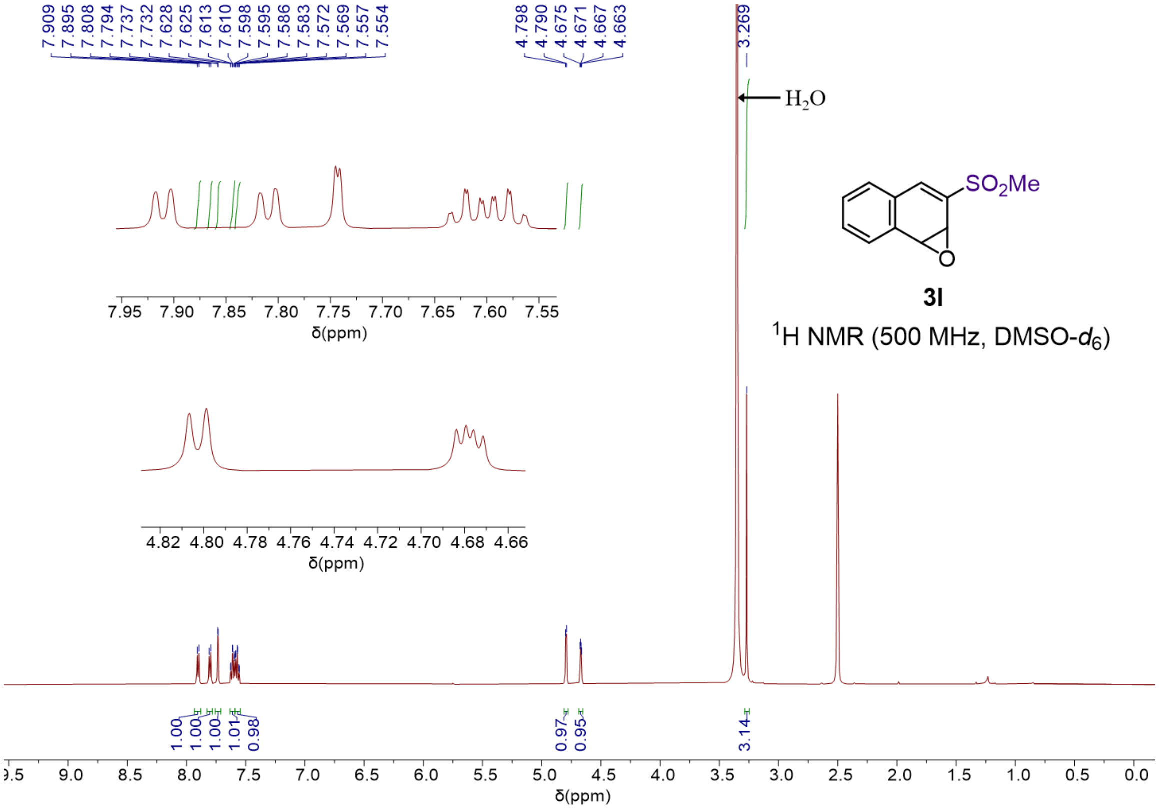

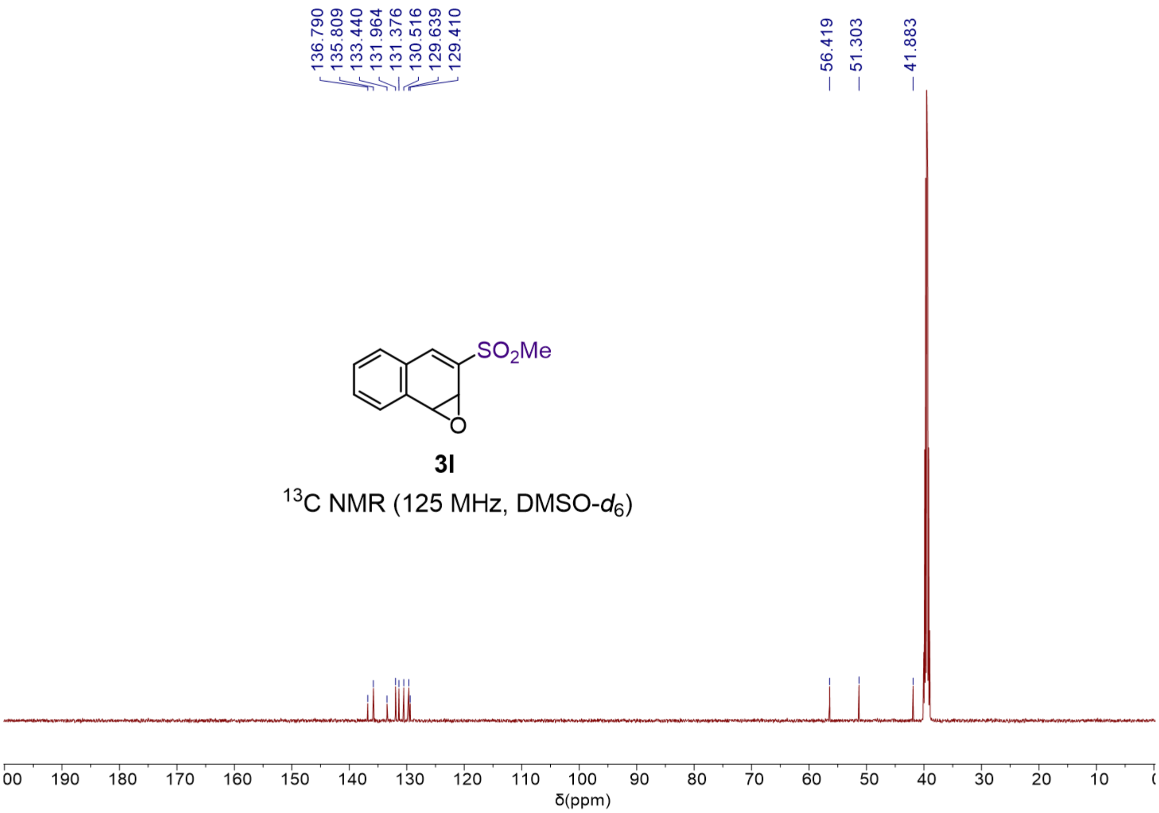

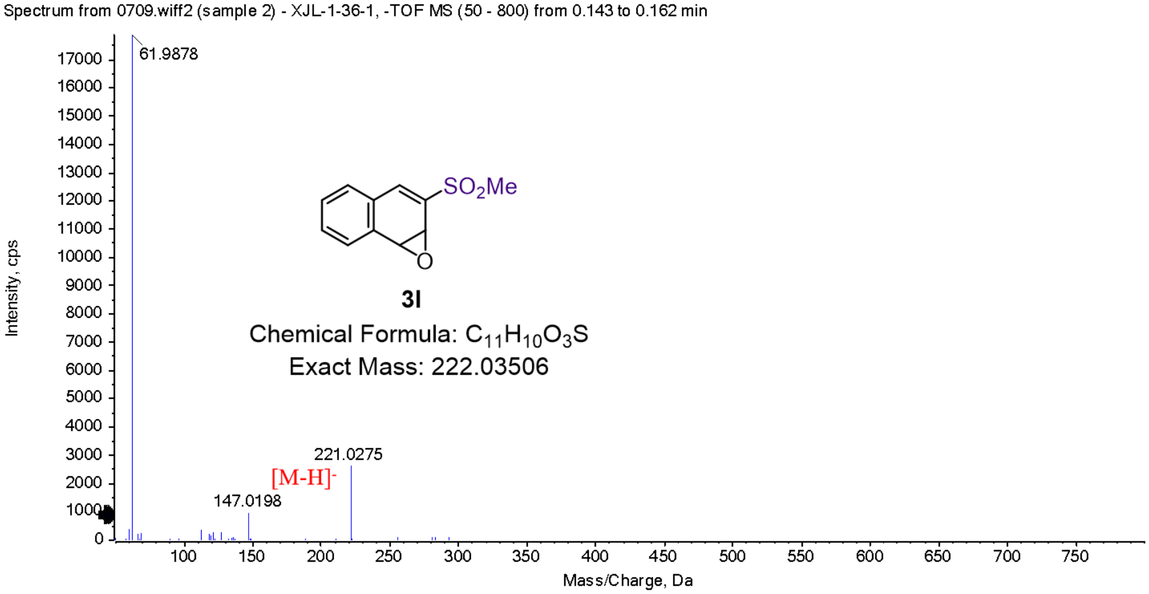

Ion Formula	Calculated *m/z*	Error (ppm)	Error (mmu)	RDB
$C_{11}H_9O_3S$	221.02779	-1.4	-0.31	7.0

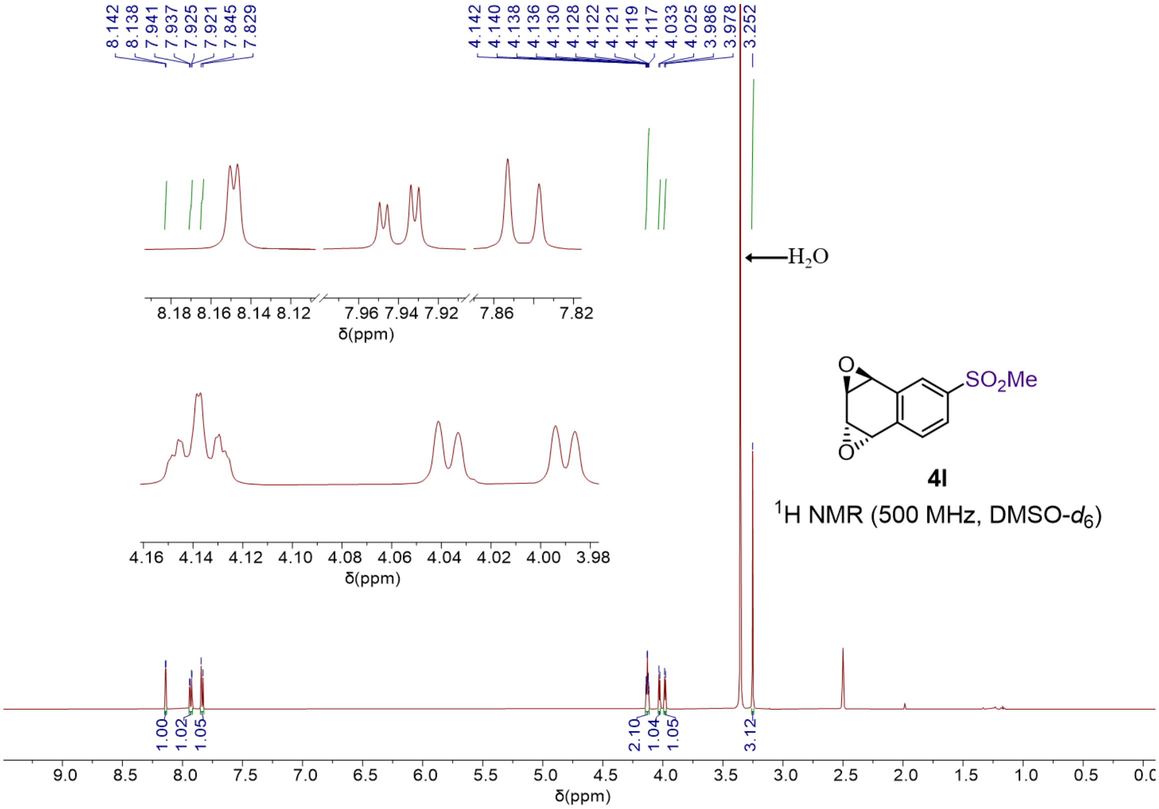
8.142
8.138
7.941
7.937
7.925
7.921
7.845
7.829
4.142
4.140
4.138
4.136
4.130
4.128
4.122
4.121
4.119
4.117
4.033
4.025
3.986
3.978
3.252
H2O
4l
1H NMR (500 MHz, DMSO-d6)
8.18 8.16 8.14 8.12
7.96 7.94 7.92
7.86
7.82
δ(ppm)
4.16 4.14 4.12 4.10 4.08 4.06 4.04 4.02 4.00 3.98
δ(ppm)
1.00
1.02
1.05
2.10
1.04
1.05
3.12
9.0 8.5 8.0 7.5 7.0 6.5 6.0 5.5 5.0 4.5 4.0 3.5 3.0 2.5 2.0 1.5 1.0 0.5 0.0
δ(ppm)

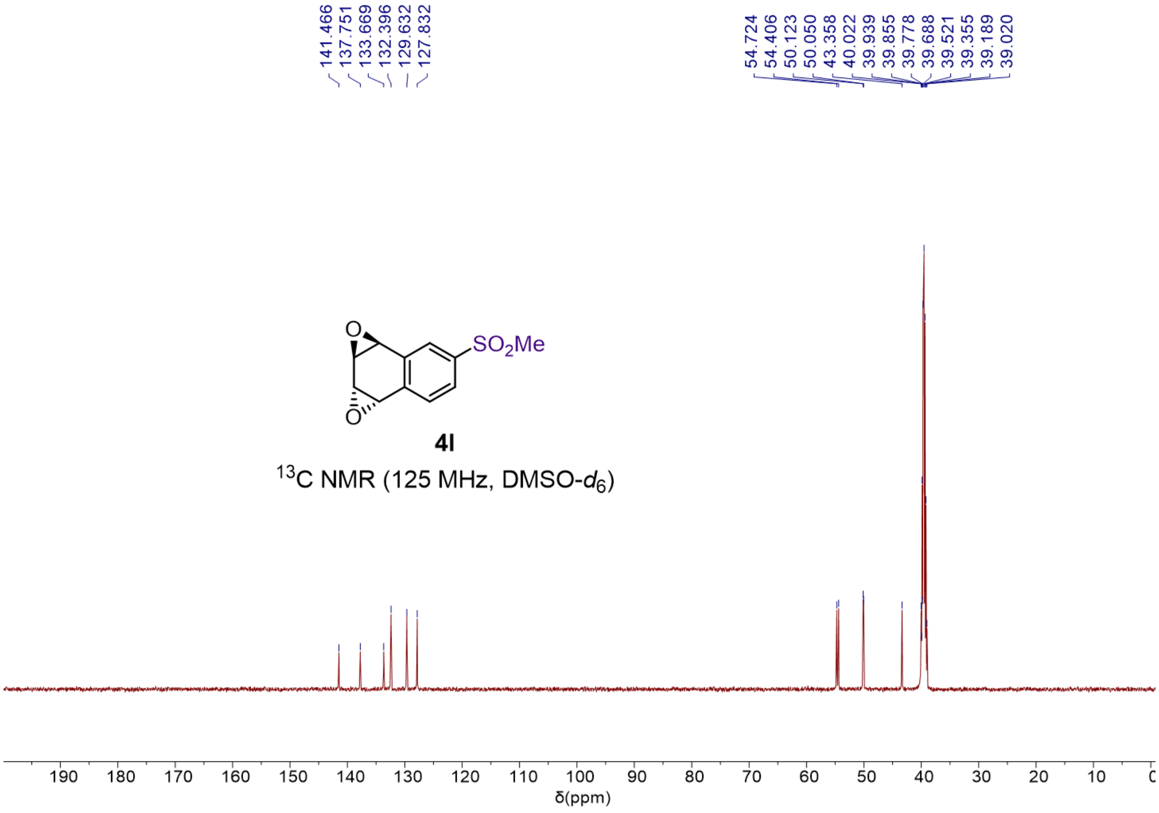
141.466
137.751
133.669
132.396
129.632
127.832
54.724
54.406
50.123
50.050
43.358
40.022
39.939
39.855
39.778
39.688
39.521
39.355
39.189
39.020
4l
13C NMR (125 MHz, DMSO-d6)
190 180 170 160 150 140 130 120 110 100 90 80 70 60 50 40 30 20 10 0
δ(ppm)

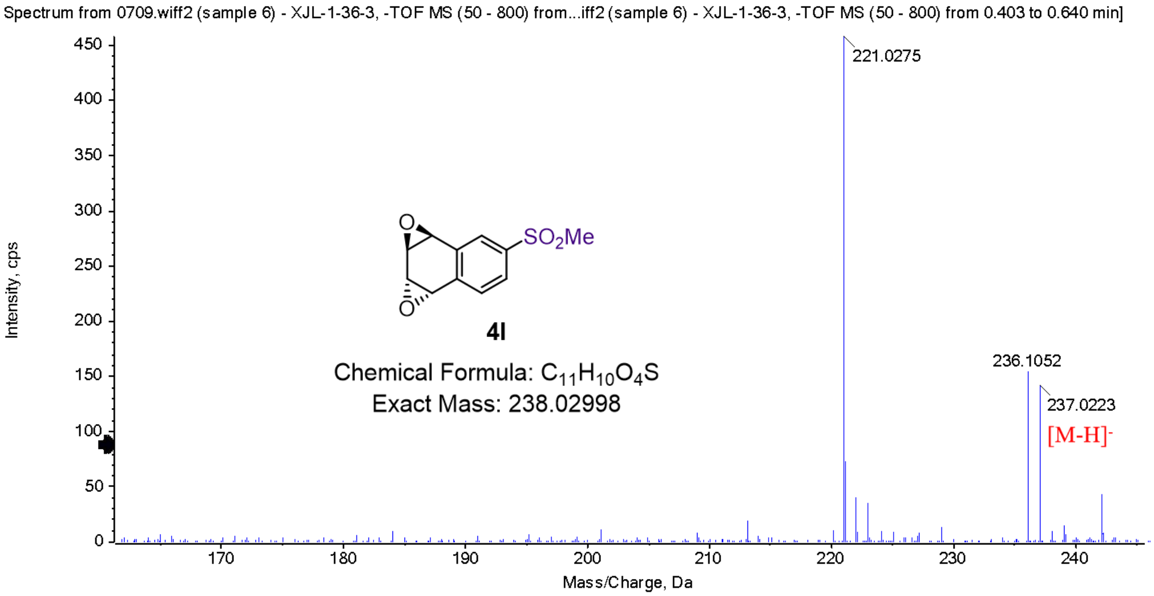

Ion Formula	Calculated *m/z*	Error (ppm)	Error (mmu)	RDB
$C_{11}H_9O_4S$	237.02270	-1.8	-0.42	7.0

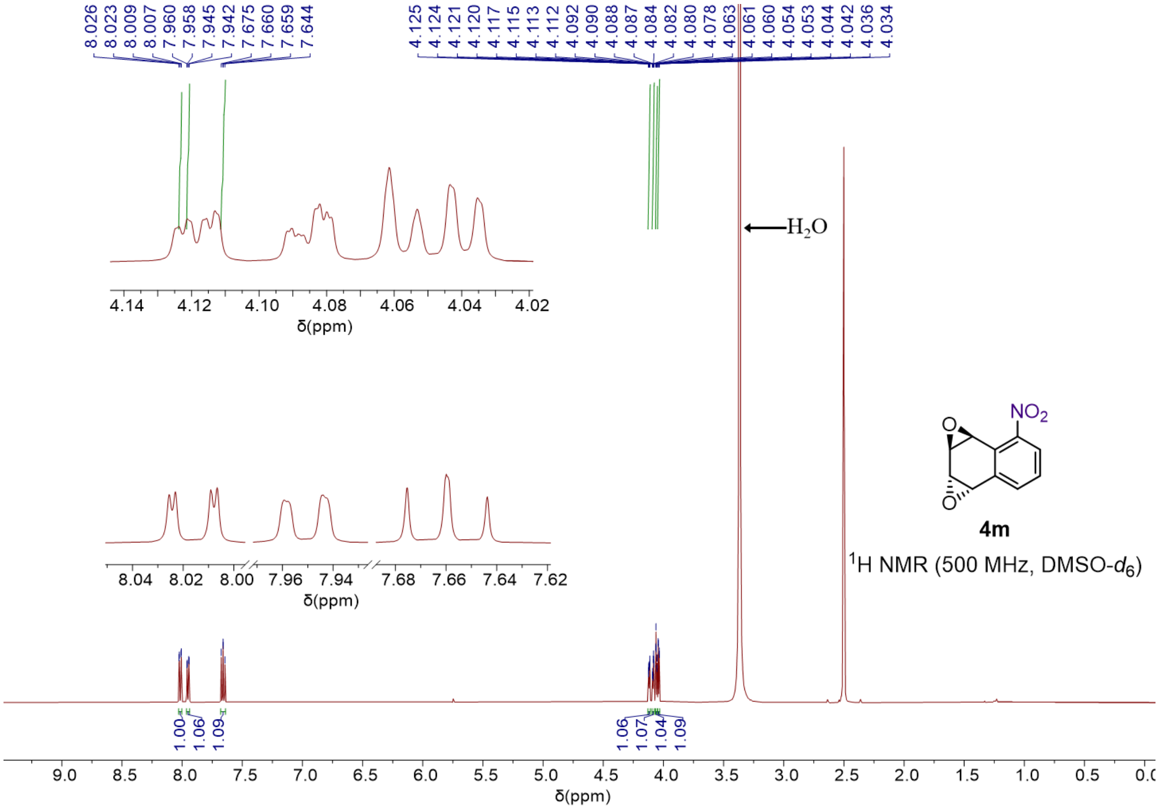

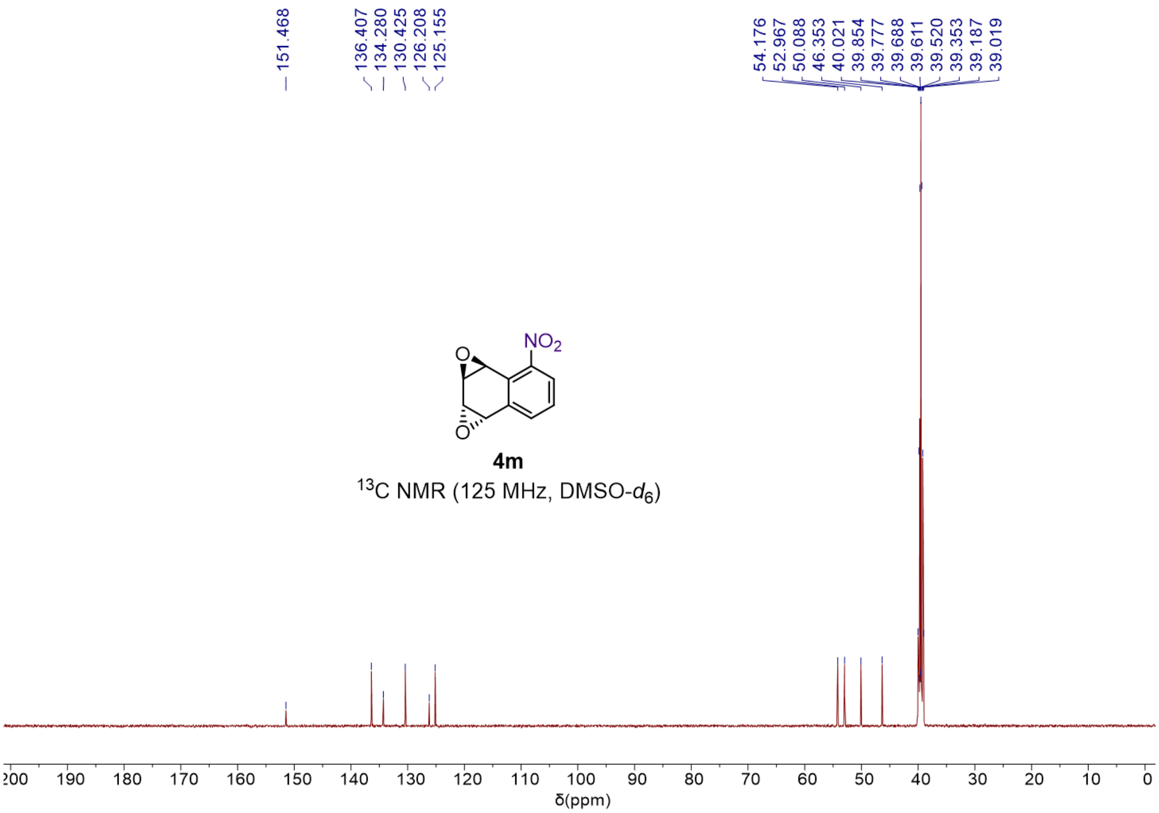

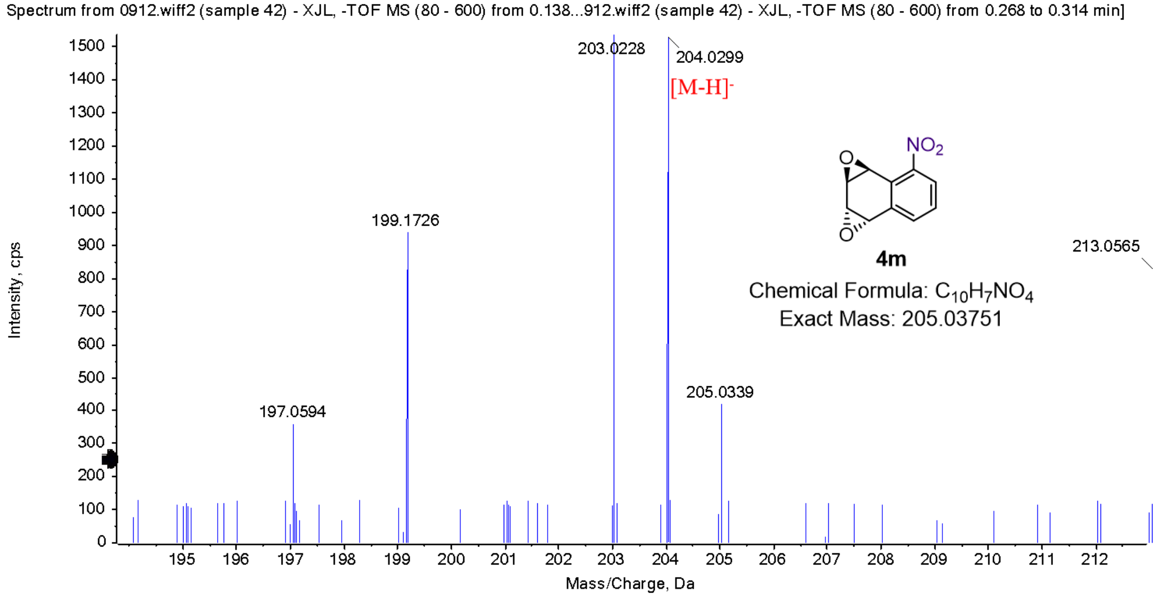

Ion Formula	Calculated *m/z*	Error (ppm)	Error (mmu)	RDB
$C_{10}H_6NO_4$	204.03023	-1.7	-0.34	8.0

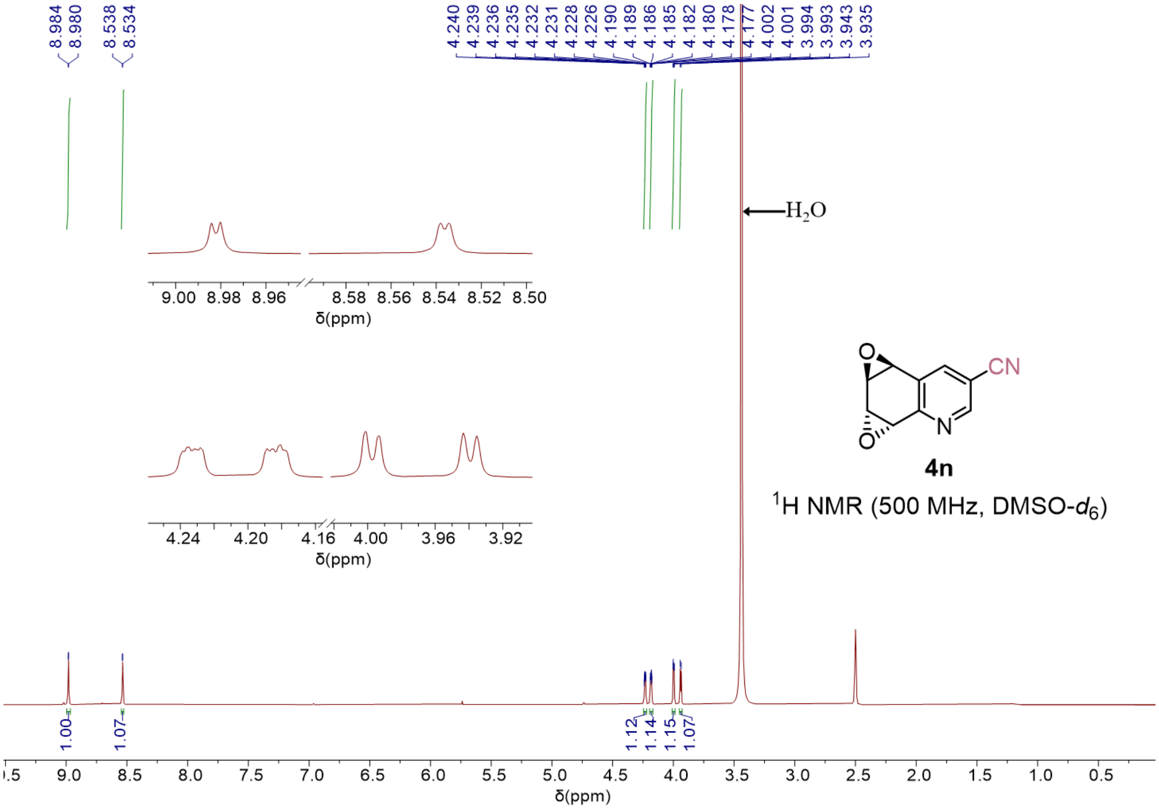
8.984
8.980
8.538
8.534
4.240
4.239
4.236
4.235
4.232
4.231
4.228
4.226
4.190
4.189
4.186
4.185
4.182
4.180
4.178
4.177
4.002
4.001
3.994
3.993
3.943
3.935
H2O
CN
N
O
O
4n
1H NMR (500 MHz, DMSO-d6)
1.00
1.07
1.12
1.14
1.15
1.07
δ(ppm)

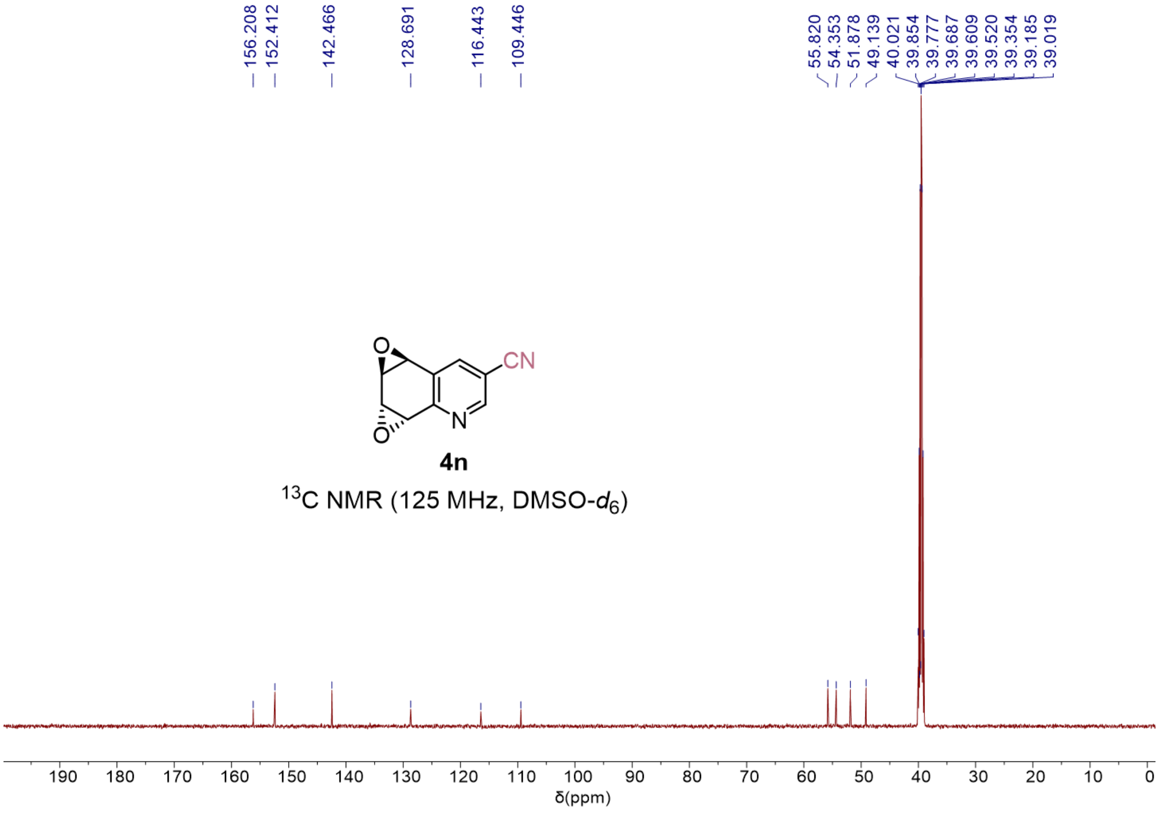
156.208
152.412
142.466
128.691
116.443
109.446
55.820
54.353
51.878
49.139
40.021
39.854
39.777
39.687
39.609
39.520
39.354
39.185
39.019
CN
N
O
O
4n
13C NMR (125 MHz, DMSO-d6)
δ(ppm)

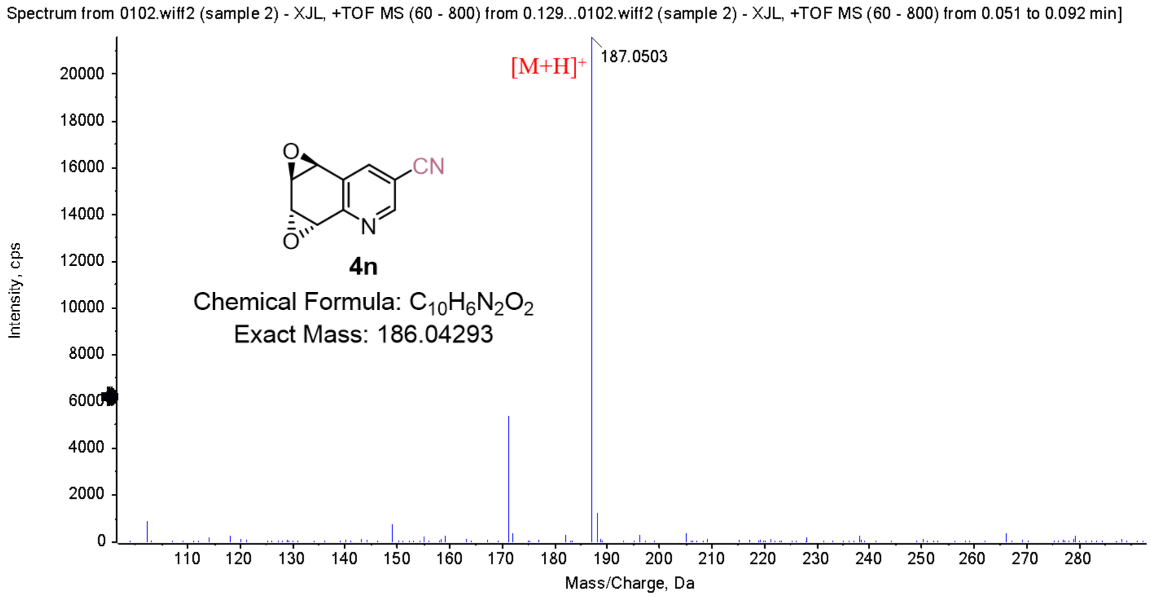

Ion Formula	Calculated *m/z*	Error (ppm)	Error (mmu)	RDB
$C_{10}H_7N_2O_2$	187.05020	0.6	0.12	9.0

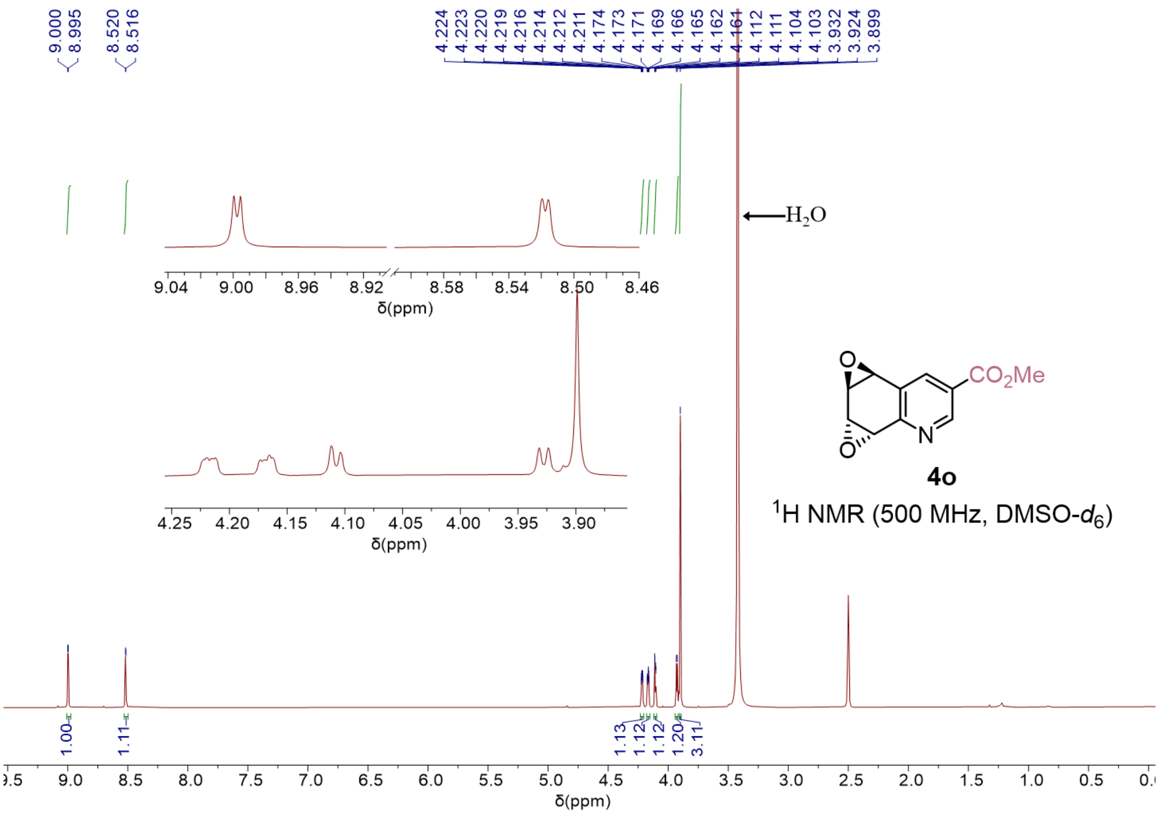

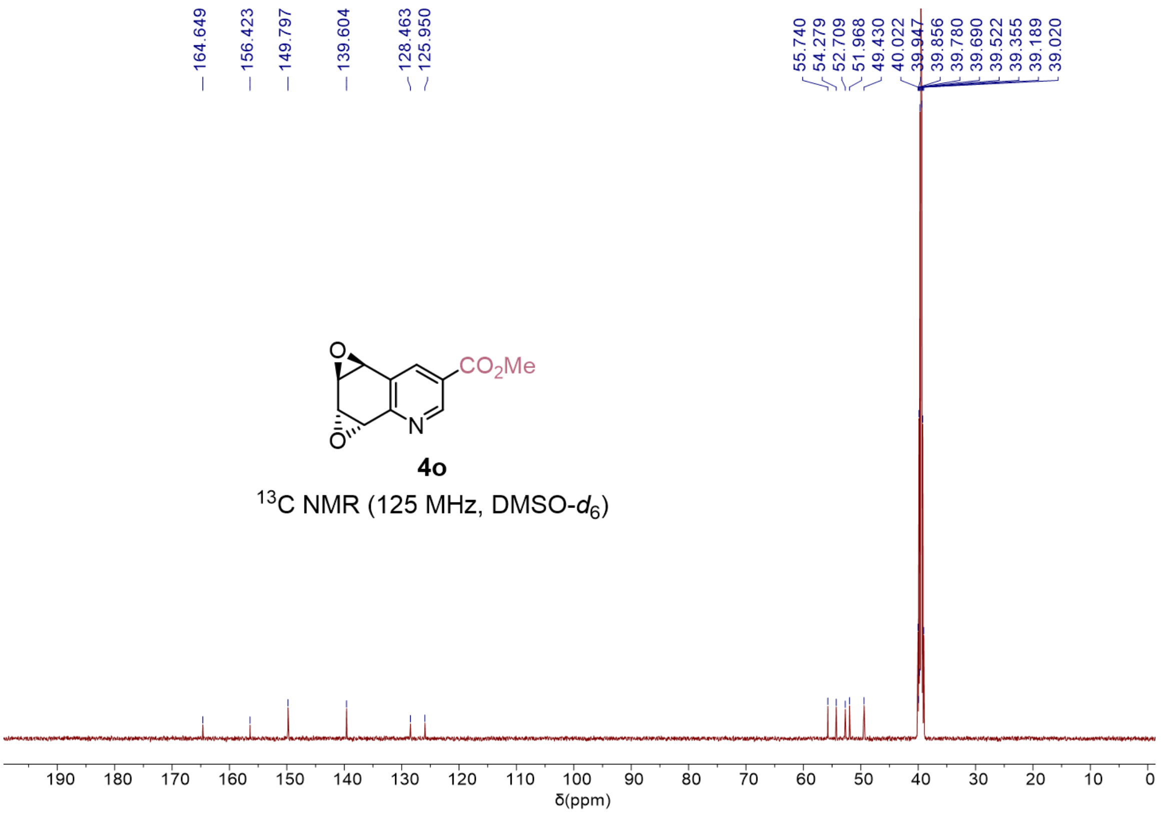

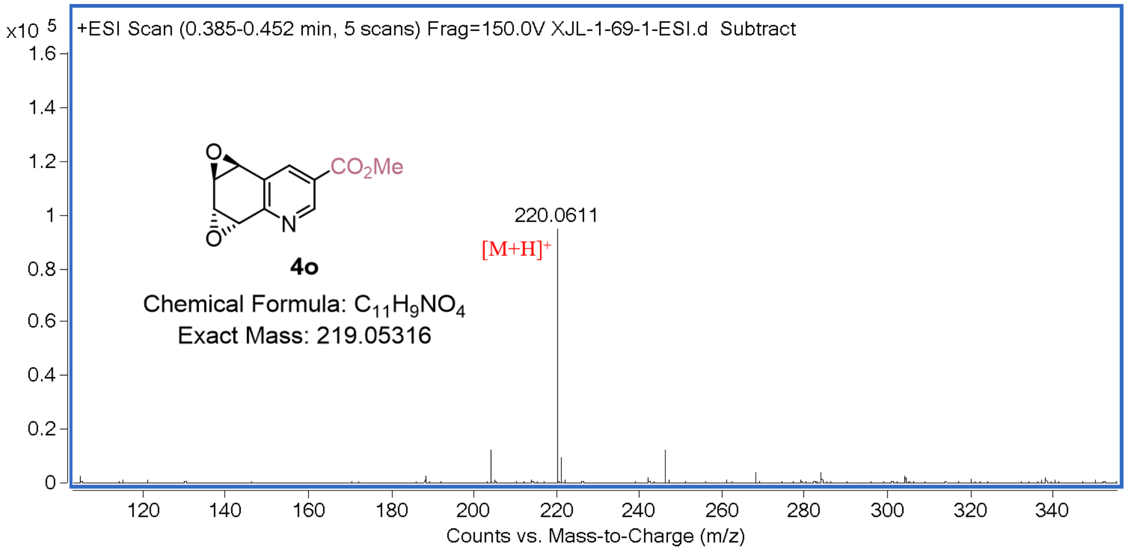

Formula (M)	Ion Formula	*m*/*z*	Calc *m*/*z*	Diff (ppm)	DBE
$C_{11}H_9NO_4$	$C_{11}H_9NO_4$	220.0611	220.0604	-3.04	8

1.9. The reaction of 3a in the absence of catalyst

To directly address the stability of epoxide intermediates, we examined the behavior of isolated **3a**, derived from **1a**, under catalytic and ambient conditions. Under catalytic conditions, **3a** gradually rearranged to the corresponding hydroxylated product. However, when stored at room temperature without catalyst for 34 days, only 14.4% conversion was observed, suggesting that the epoxide is inherently stable and that rearrangement is catalytically driven. From this conversion, the hydroxylation yield was calculated to be 66.7%.

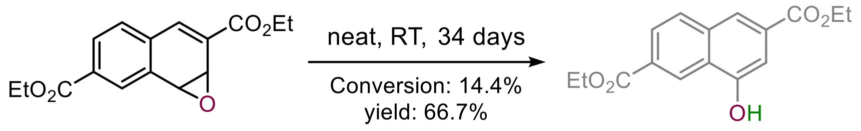

3a **5a'**

Fig. S1 The reaction of 3a in the absence of catalyst.

Synthesis procedure: Compound **3a** was synthesized according to the general procedure. The obtained **3a** (104 mg) was then exposed to air for 34 days. The crude mixture was purified by silica gel column chromatography to recycle naphthalene-2,6-dicarboxylate (89 mg) in 14.4% conversion, and provide the compound **5a'** (petroleum ether/ethylacetate = 30/1, *v*/*v*) as a red solid 10 mg in 3.5% yield, which is 66.7% on the basis of 14.4% conversion. **5a'**: ^{1}H NMR (500 MHz, $CDCl_3$) δ (ppm): 3.91 (s, 3H), 3.95 (s, 1H), 4.55 (d, J = 3.5 Hz, 1H), 4.71 (dd, J = 4.0, 2.0 Hz, 1H), 7.77 (d, J = 8.0 Hz, 1H), 7.80 (d, J = 2.0 Hz, 1H), 8.12 (dd, J = 8.0, 2.0 Hz, 1H), 8.17 (d, J = 2.0 Hz, 1H). ^{13}C NMR (125 MHz, $CDCl_3$) δ (ppm): 14.48, 14.54, 61.52, 61.72, 108.59, 122.97, 125.28, 126.19, 126.72, 129.2, 129.43, 130.22, 130.03, 153.34, 166.80, 166.85. HRMS (ESI) *m*/*z*: calcd for $C_{16}H_{15}O_5^-$ $[M–H]^-$ 287.0914, found 287.0932.

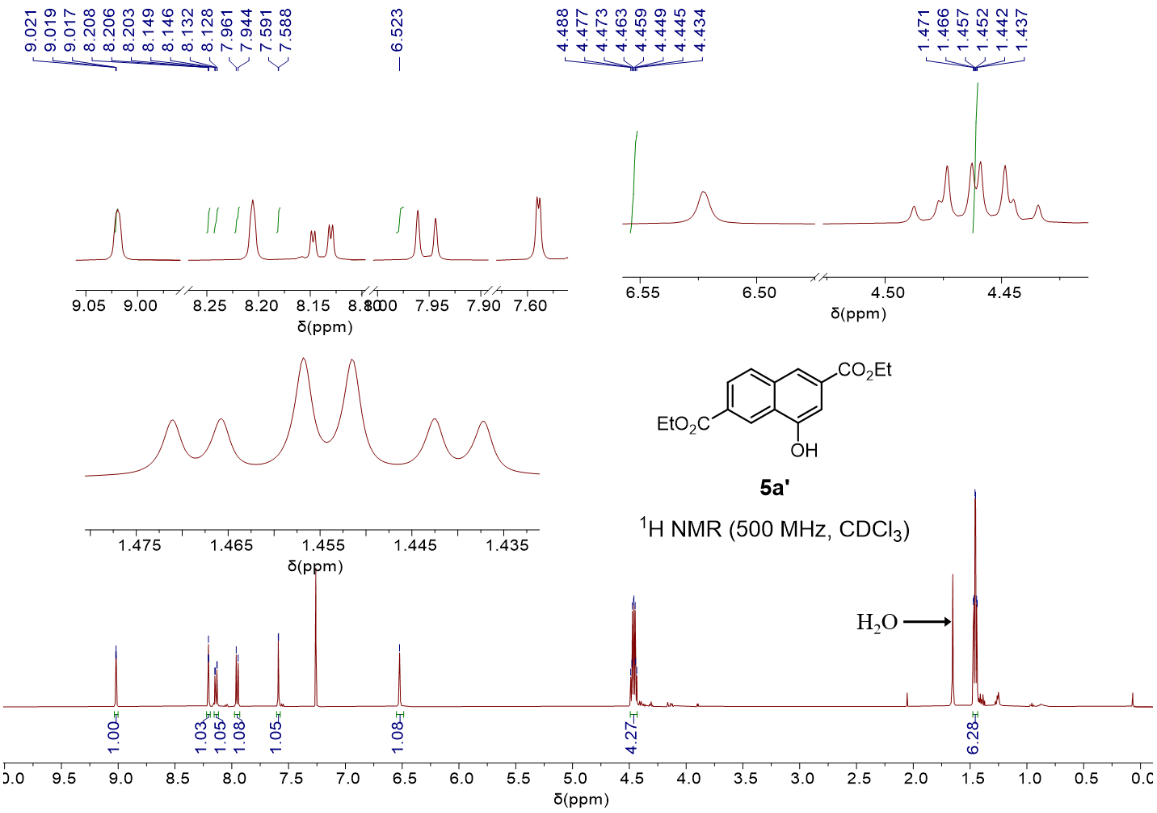
9.021 9.019 9.017 8.208 8.206 8.203 8.149 8.146 8.132 8.128 7.961 7.944 7.591 7.588
6.523
4.488 4.477 4.473 4.463 4.459 4.449 4.445 4.434
1.471 1.466 1.457 1.452 1.442 1.437
CO2Et
EtO2C
OH
5a'
1H NMR (500 MHz, CDCl3)
H2O
1.00 1.03 1.05 1.08 1.05 1.08 4.27 6.28
δ(ppm)

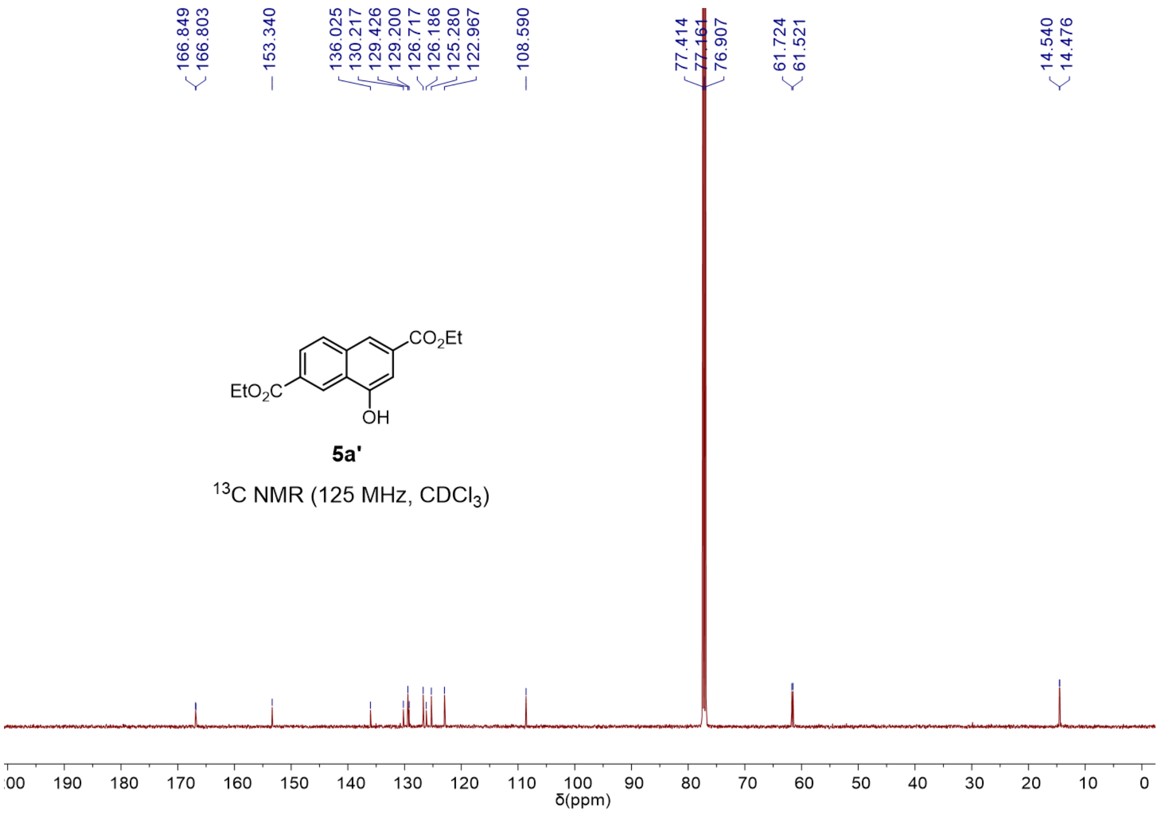
166.849 166.803
153.340
136.025 130.217 129.426 129.200 126.717 126.186 125.280 122.967
108.590
77.414 77.161 76.907
61.724 61.521
14.540 14.476
CO2Et
EtO2C
OH
5a'
13C NMR (125 MHz, CDCl3)
δ(ppm)

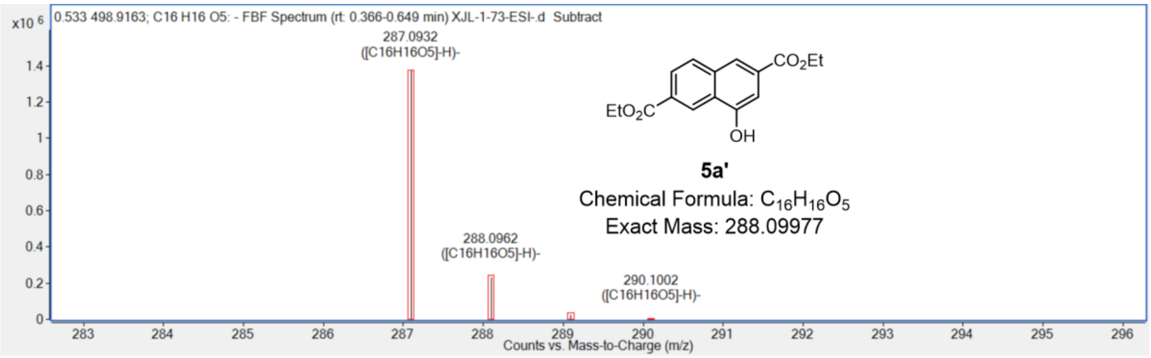

1.10. The reaction of naphthalene

The reactivity of naphthalene **1u** was examined (Fig. S2). The results showed that the reaction mainly formed the quinone **5u**.

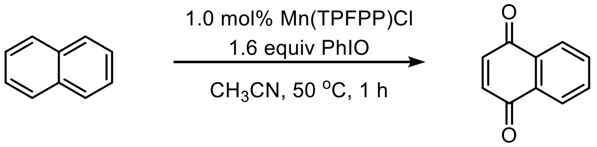

Fig. S2 The reaction of naphthalene.

Synthesis and characterization: A 38 mL oven-dried sealed tube (with a Teflon cap), equipped with a magnetic stir bar, was charged with naphthalene **1u** (256 mg, 2.0 mmol, 1.0 equiv.), Mn(TPFPP)Cl (22 mg, 0.02 mmol, 1.0 mol%) and PhIO (704 mg, 3.2 mmol, 1.6 equiv.) in CH_3CN (10 mL) at 50 °C for 1 h. The solution was cooled to room temperature, then concentrated under reduced pressure. The crude mixture was purified by silica gel column chromatography to recycle **1u** (128 mg) in 50.0% conversion, and provide the compound **5u** (petroleum ether/ethylacetate = 30/1, *v*/*v*) as a red oil 32 mg in 14.8% yield, which is 20.6% on the basis of 50.0% conversion. **5u**: ^{1}H NMR (500 MHz, $CDCl_3$) δ (ppm): 7.00 (dd, J = 14.5, 4.0 Hz, 2H), 7.27 (td, J = 8.5, 3.0 Hz, 1H), 7.40 (dd, J = 8.0, 2.0 Hz, 1H), 8.14 (d, J = 8.5, 5 Hz, 1H). The ^{1}H NMR data was in agreement with that reported in the literature[5].

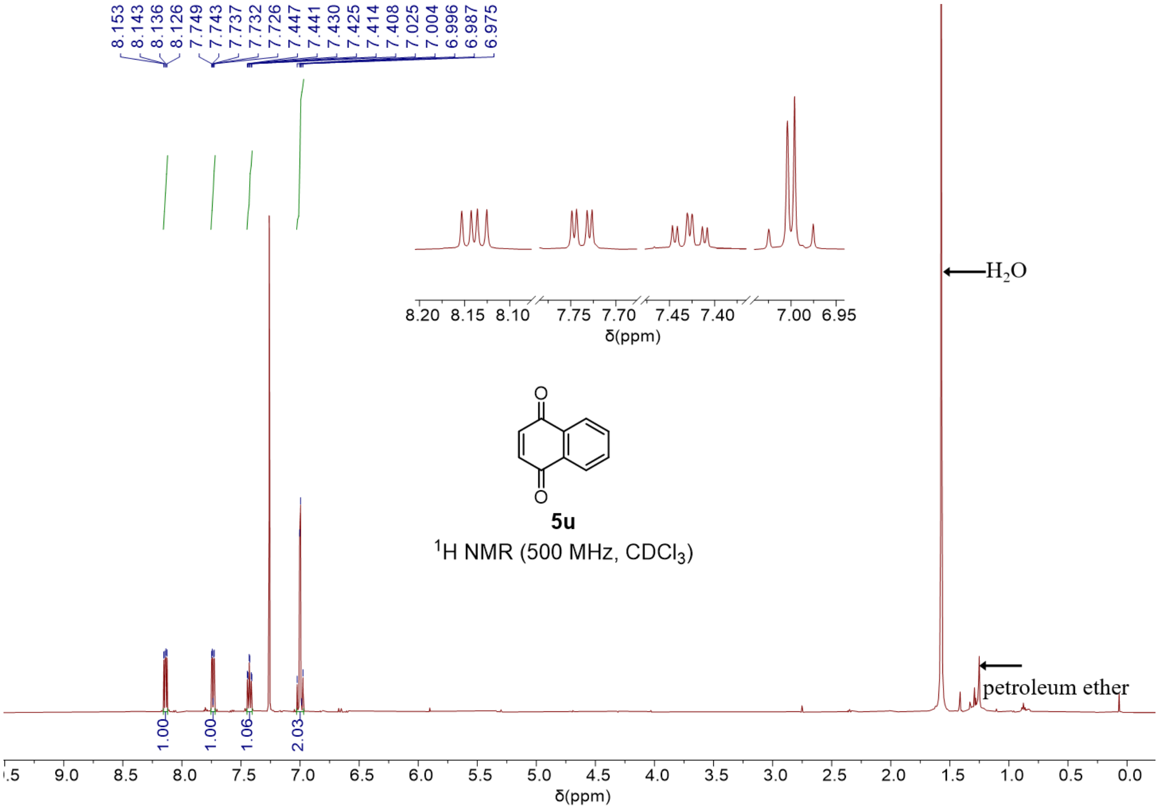

1.11. The effect of substitution pattern

To evaluate the influence of substitution pattern on chemoselectivity, we conducted parallel experiments using 2,7-dimethoxynaphthalene **1s** and 2,6-dimethoxynaphthalene **1t** under identical reaction conditions (Fig. S3a). Both substrates afforded the corresponding

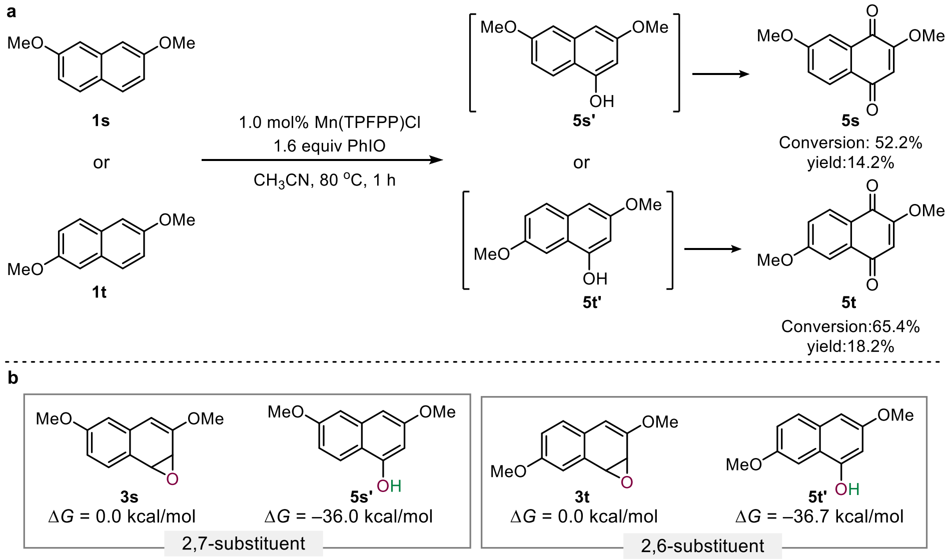

Fig. S3. The effect of substitution pattern. a Comparative experiments. **b** Thermodynamic stability.

quinone products in comparable yields. Moreover, we performed DFT calculations on the relative stability of the epoxide and hydroxylation product derived from each substrate (Fig. S3b). The results showed that both substitution patterns lead to products with comparable stabilities. Taken together, these experimental and computational results indicate that the observed chemoselectivity is not strongly affected by the substitution pattern.

Synthesis and characterization: A 38 mL oven-dried sealed tube (with a Teflon cap), equipped with a magnetic stir bar, was charged with 2,7-dimethoxynaphthalene **1s** (188 mg, 1.0 mmol, 1.0 equiv), Mn(TPFPP)Cl (11 mg, 0.01 mmol, 1.0 mol%) and PhIO (352 mg, 1.6 mmol, 1.6 equiv.) in CH_3CN (5 mL) at 80 °C for 1 h. The solution was cooled to room temperature, then concentrated under reduced pressure. The crude mixture was purified by silica gel column chromatography to recycle **1s** (90 mg) in 52.2% conversion, and provide the compound **5s** (petroleum ether/ethylacetate = 20/1, *v*/*v*) as a yellow solid 16 mg in 7.3% yield, which is 14.2% on the basis of 52.2% conversion. **5s**: ^{1}H NMR (500 MHz, $CDCl_3$) δ (ppm): 3.89 (s, 3H), 3.95 (s, 3H), 6.11 (s, 1H), 7.21 (dd, J = 8.5, 2.5 Hz, 1H), 7.57 (d, J = 2.5 Hz, 1H), 8.03 (d, J = 8.5 Hz, 1H).

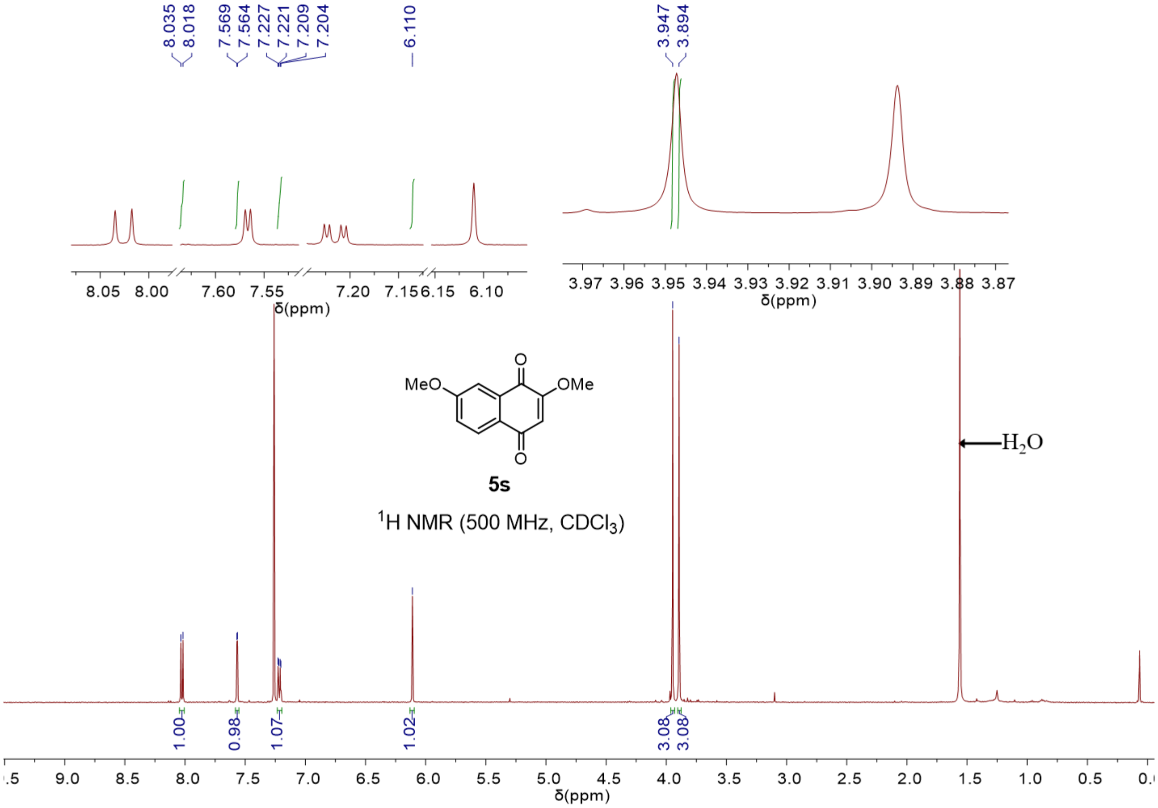

Synthesis and characterization: A 38 mL oven-dried sealed tube (with a Teflon cap), equipped with a magnetic stir bar, was charged with 2,6-dimethoxynaphthalene **1t** (188 mg, 1.0 mmol, 1.0 equiv.), Mn(TPFPP)Cl (11 mg, 0.01 mmol, 1.0 mol%) and PhIO (352 mg, 1.6

mmol, 1.6 equiv.) in CH_3CN (5 mL) at 80 °C for 1 h. The solution was cooled to room temperature, then concentrated under reduced pressure. The crude mixture was purified by silica gel column chromatography to recycle **1t** (65 mg) in 65.4% conversion, and provide the compound **5t** (petroleum ether/ethylacetate = 25/1, *v*/*v*) as a yellow solid 26 mg in 11.9% yield, which is 18.2% on the basis of 65.4% conversion. **5t**: ^{1}H NMR (500 MHz, $CDCl_3$) δ (ppm): 3.90 (s, 3H), 3.95 (s, 3H), 6.13 (s, 1H), 7.17 (dd, J = 8.5, 2.5 Hz, 1H), 7.53 (d, J = 2.5 Hz, 1H), 8.08 (d, J = 8.5 Hz, 1H). The ^{1}H NMR data was in agreement with that reported in the literature[7].

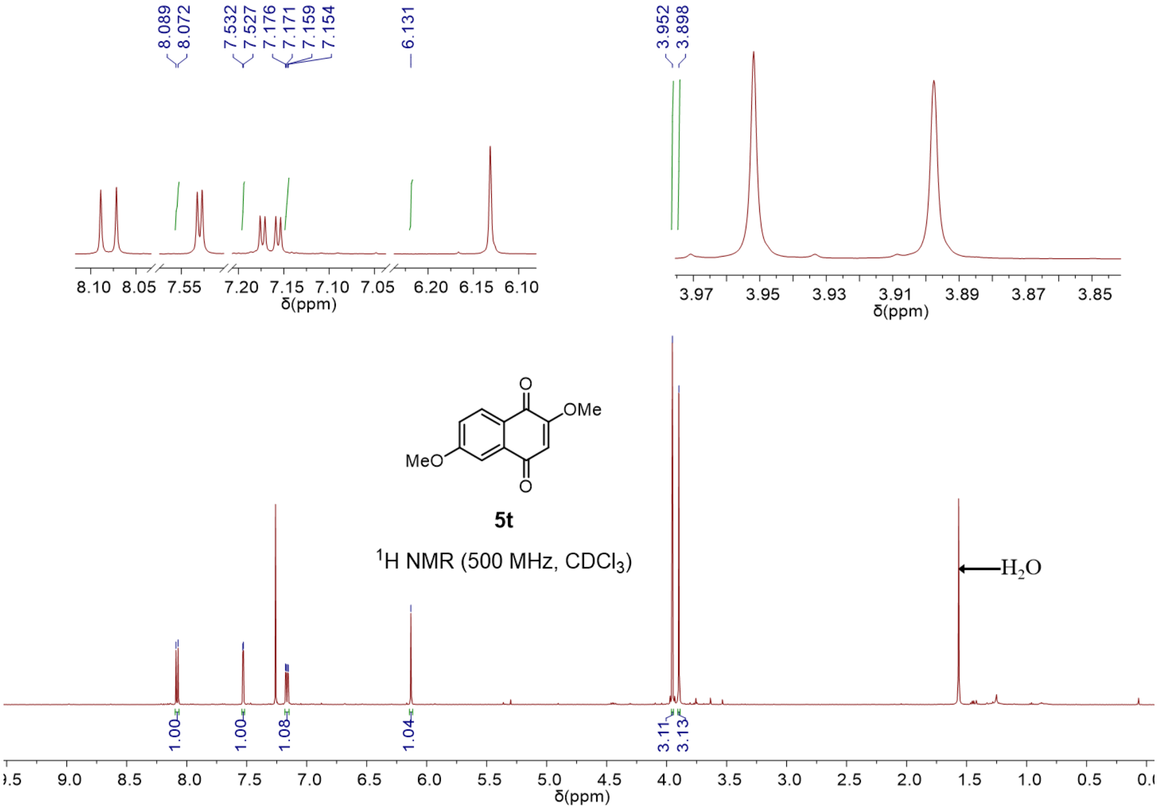

1.12. Mechanistic experiments

1.12.1. Monitoring the reaction of 1s

In order to investigate the potential involvement of an epoxide intermediate, we monitored the reaction of 2,7-dimethoxynaphthalene (**1s**) over time. Thin-layer chromatography and mass spectrometry showed that quinone product **5s** formed within 1–10 minutes, no detectable intermediate spots between **1s** and **5s** including the epoxide **3s** (Fig. S4a). After extended monitoring (10–60 minutes), no trace of **3s** was detected. While this does not definitively exclude the transient formation of an epoxide, the data suggest it is not an intermediate in this case. Additionally, we calculated the relative thermodynamic stability of

the epoxide **3** and hydroxylation product **5'**. For substrate **1a** and **1s**, the hydroxylation products **5a'** and **5s'** were more stable than the corresponding epoxides **3a** and **3s** by 37.1 and 36.0 kcal/mol, respectively. This suggests that both electron-rich and electron-poor epoxides are similarly less favorable thermodynamically. Importantly, our DFT results suggest that these substrates follow distinct reaction mechanisms: **1a** reacts via a radical pathway leading epoxidation, while **1s** predominantly undergoes hydroxylation through a zwitterionic pathway. Taken together, these findings demonstrate that the formation of the hydroxylation product may bypass an epoxide intermediate for **1s**.

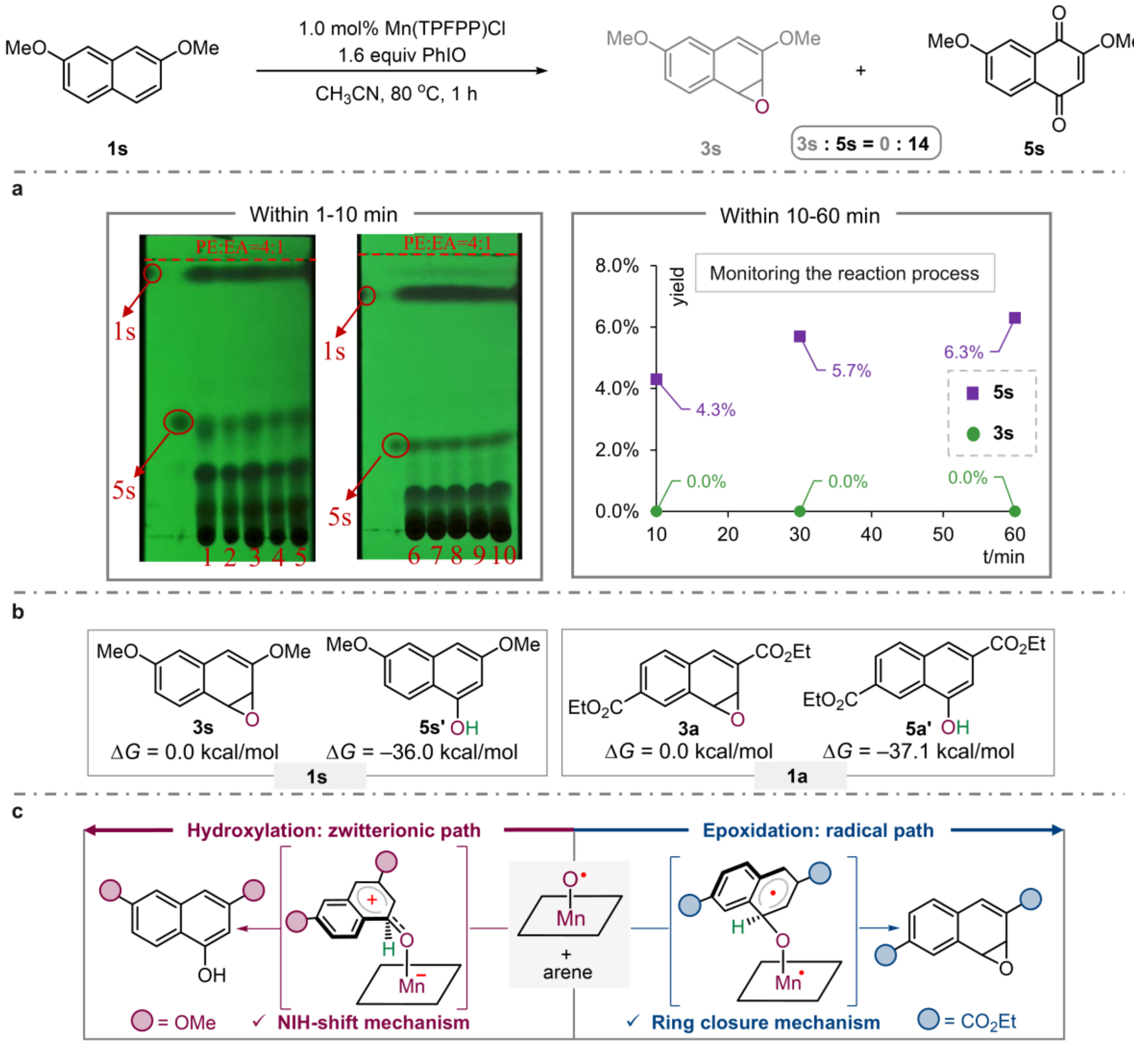

Fig. S4 Monitoring the reaction of 1s. a Monitoring the reaction process of **1s**. petroleum ether/ethylacetate = PE/EA. The yields were determined by 1H NMR analyses of the crude products using CH_2Br_2 as the internal standard. **b** the thermodynamic stability of epoxide **3** and hydroxylation product **5'**. **c** DFT calculations.

Experimental procedure: A 38 mL oven-dried sealed tube (with a Teflon cap), equipped with a magnetic stir bar, was charged with 2,7-dimethoxynaphthalene **1s** (188 mg, 1.0 mmol, 1.0 equiv.), Mn(TPFPP)Cl (11 mg, 0.01 mmol, 1.0 mol%) and PhIO (352 mg, 1.6 mmol, 1.6 equiv.) in CH_3CN (5 mL) at 80 °C for 10 min. The solution was cooled to room temperature, then concentrated under reduced pressure. After cooling the solution to room temperature, it was concentrated under reduced pressure. The resulting crude mixture was subjected to purification via silica gel column chromatography, during which all fractions eluted between the starting material (**1s**) and the quinone product (**5s**) were collected. The combined fractions were concentrated under vacuum to afford a red oil, which was subsequently analyzed by mass spectrometry and found to contain no **3s**.

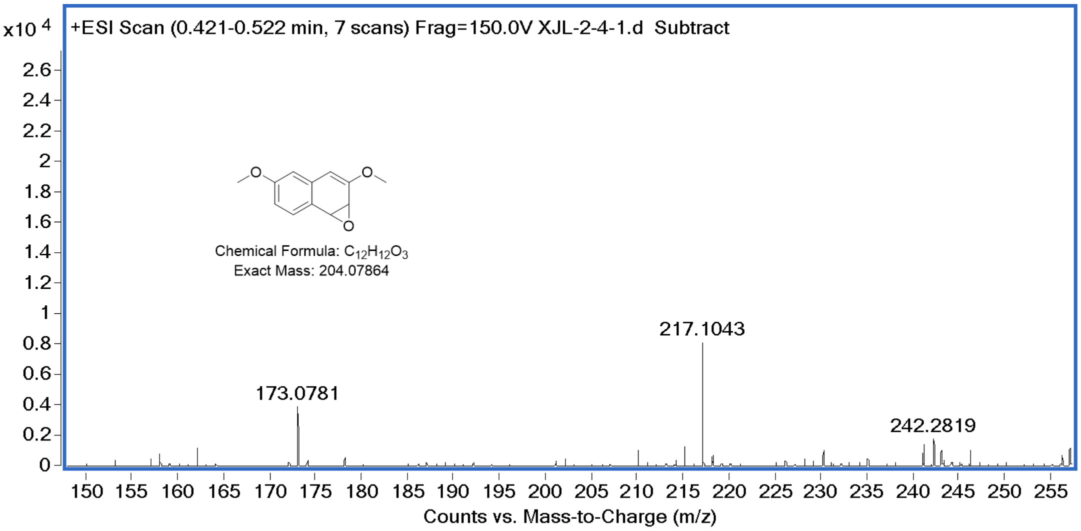

1.12.2. The kinetic isotope effect (KIE) experiments

We also conducted kinetic isotope effect (KIE) experiments on **1s** and [D]-**1s** (Fig. S5). The observed KIE value of 1.025 indicates that C−H bond cleavage is unlikely to be the rate-determining step, which is consistent with our computational results. Unfortunately, a deuterated analog of **1a** was not available, so KIE experiments were limited to **1s**.

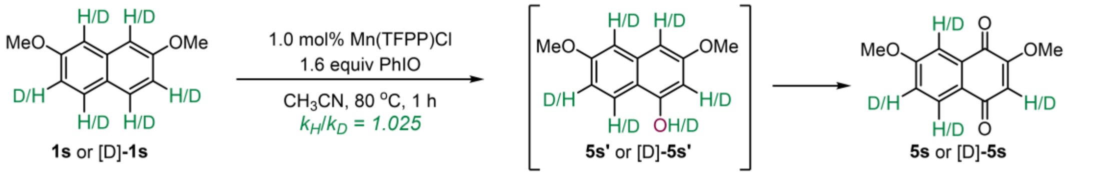

t/h	**5s**/yield	[D]-**5s**/yield
1 h	6.3%	4.6%
2 h	6.8%	5.2%
3 h	7.6%	6.0%
4 h	8.2%	6.6%
5 h	9.0%	7.5%
6 h	10.6%	8.7%

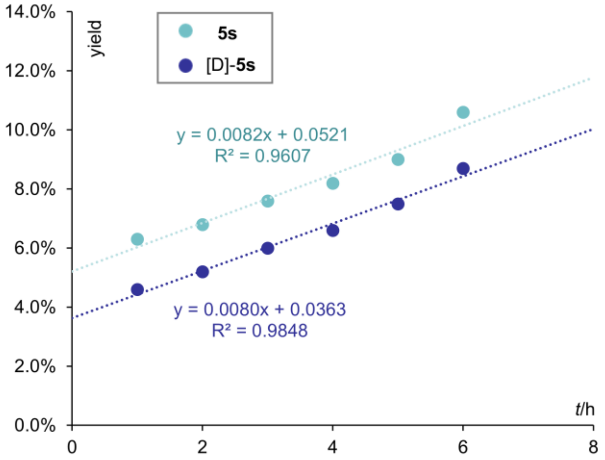

Fig. S5 The kinetic isotope effect (KIE) experiments of 1s and [D]-1s. The yields were determined by ^{1}H NMR analyses of the crude products using CH_2Br_2 as the internal standard.

Experimental procedure: The kinetic isotope effect (KIE) was examined by applying the initial rate method. A 38 mL oven-dried sealed tube (with a Teflon cap), equipped with a magnetic stir bar, was charged with 2,7-dimethoxynaphthalene (35 mg, 0.184 mmol, 1.0 equiv.), Mn(TPFPP)Cl (2 mg, 0.0184 mmol, 1.0 mol%) and PhIO (65 mg, 0.294 mmol, 1.6 equiv.) in CH_3CN (2.0 mL) at 80 °C. The yields were determined by ^{1}H NMR analyses of the crude products using CH_2Br_2 as the internal standard. Then the same procedure was applied with 2,7-dimethoxynaphthalene-1,3,4,5,6,8-d_6.

Synthesis and characterization: A 38 mL oven-dried sealed tube (with a Teflon cap), equipped with a magnetic stir bar, was charged with 2,7-dimethoxynaphthalene [D]-**1s** (194 mg, 1.0 mmol, 1.0 equiv.), Mn(TPFPP)Cl (11 mg, 0.01 mmol, 1.0 mol%) and PhIO (352 mg, 1.6 mmol, 1.6 equiv.) in CH_3CN (5 mL) at 80 °C for 1 h. The solution was cooled to room temperature, then concentrated under reduced pressure. The crude mixture was purified by silica gel column chromatography to recycle [D]-**1s** (102 mg) in 47.4% conversion, and provide the compound [D]-**5s** (petroleum ether/ethylacetate = 20/1, *v*/*v*) as a yellow solid 12 mg in 5.4% yield, which is 11.3% on the basis of 47.4% conversion. [D]-**5s**: ^{1}H NMR (500 MHz, $CDCl_3$) δ (ppm): 3.89 (s, 3H), 3.95 (s, 3H). ^{13}C NMR (125 MHz, $CDCl_3$) δ (ppm): 56.11, 56.53, 125.57, 133.03, 160.36, 163.85, 180.47, 184.38. HRMS (ESI) *m*/*z*: calcd for $C_{12}H_6D_4NaO_4^+$ $[M+Na]^+$ 245.0722, found 245.0724.

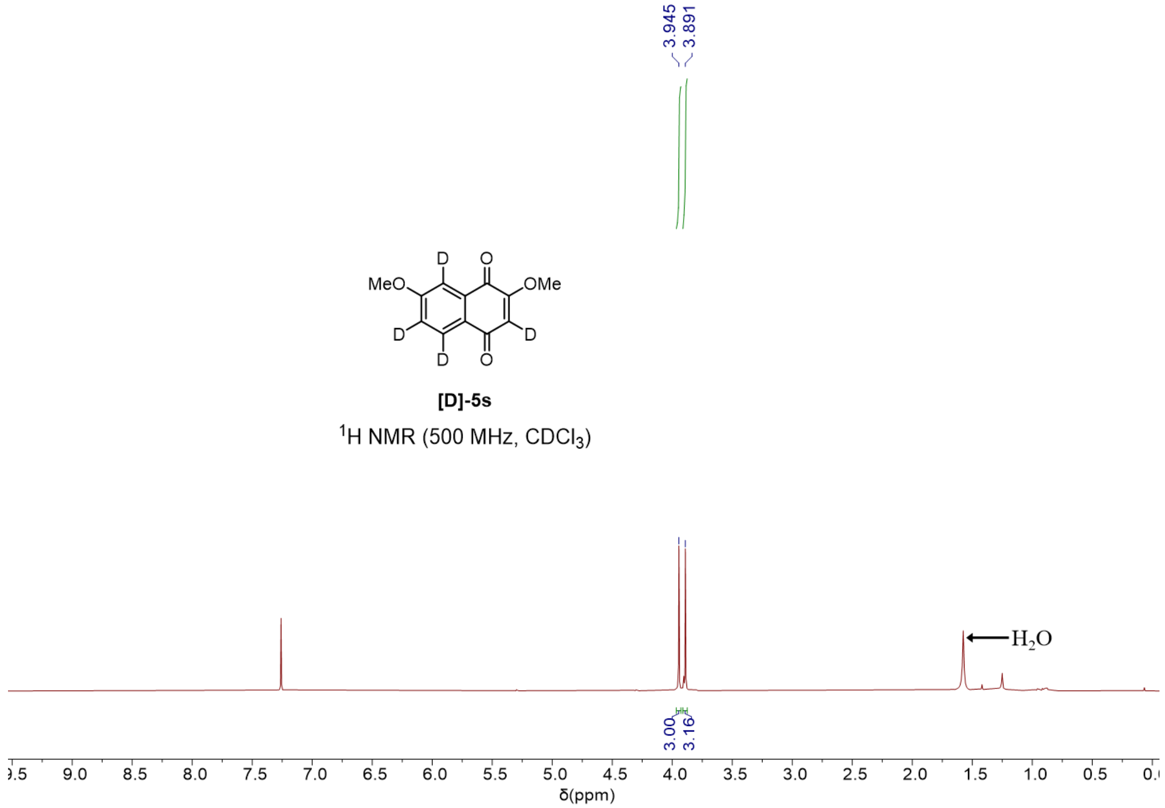
3.945
3.891
MeO
OMe
D
O
[D]-5s
^{1}H NMR (500 MHz, $CDCl_3$)
H_2O
3.00
3.16
9.5 9.0 8.5 8.0 7.5 7.0 6.5 6.0 5.5 5.0 4.5 4.0 3.5 3.0 2.5 2.0 1.5 1.0 0.5 0.0
δ(ppm)

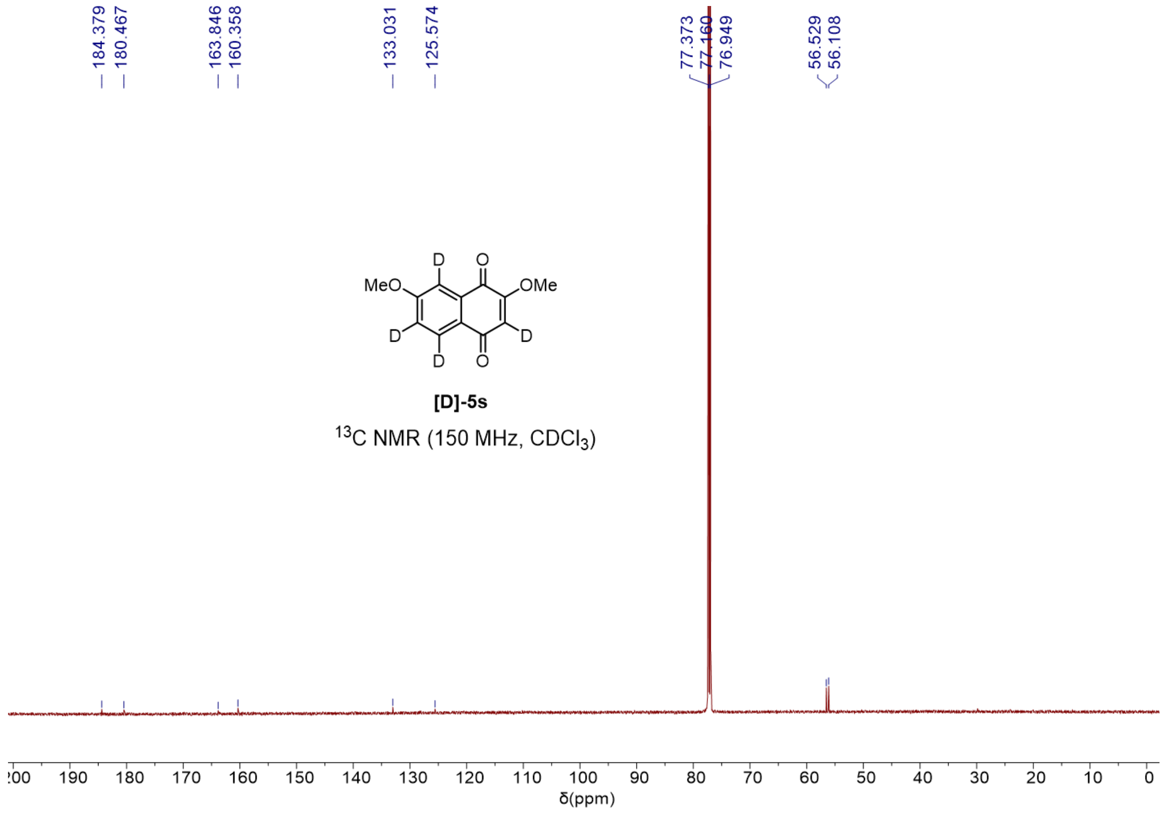
184.379
180.467
163.846
160.358
133.031
125.574
77.373
77.160
76.949
56.529
56.108
MeO
OMe
D
O
[D]-5s
^{13}C NMR (150 MHz, $CDCl_3$)
200 190 180 170 160 150 140 130 120 110 100 90 80 70 60 50 40 30 20 10 0
δ(ppm)

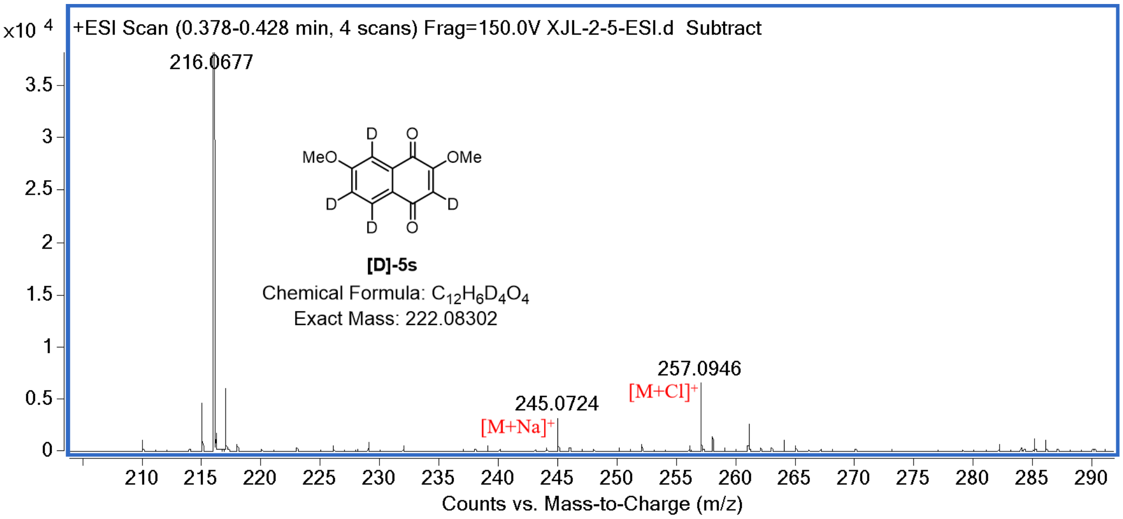

Formula (M)	Ion Formula	*m*/*z*	Calc *m*/*z*	Diff (ppm)	DBE
$C_{12}H_6D_4O_4$	$C_{12}H_6D_4NaO_4$	245.0724	245.0722	-0.73	8

2. Mechanistic Details

2.1. Possible spin states

The calculations indicate that the energy of the open-shell singlet state Mn(III) porphyrin catalyst **2** is 29.4 kcal/mol, much higher than its triplet and quintet states. Therefore, the singlet state is not considered in the subsequent pathways. Additionally, we also found that during the oxidation process, the quintet state is more favorable than the triplet state.

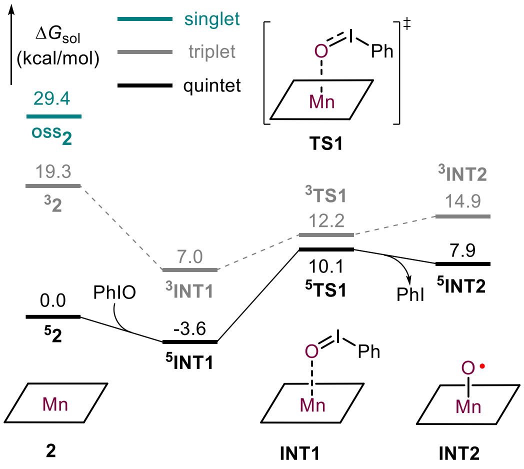

Fig. S6. The DFT-calculated possible spin states for oxidation process.

It was found that starting from the **⁵INT2**, the triplet state oxygen atom attack transition state **TS2** is more favorable than the quintet state. However, for the **INT3**, the quintet state becomes more favorable. The entire oxygen atom attack process sequentially undergoes through two minimum energy crossing points (MECP1 and MECP2). The energy of MECP1 and MECP2 are detailed in Fig. S8.

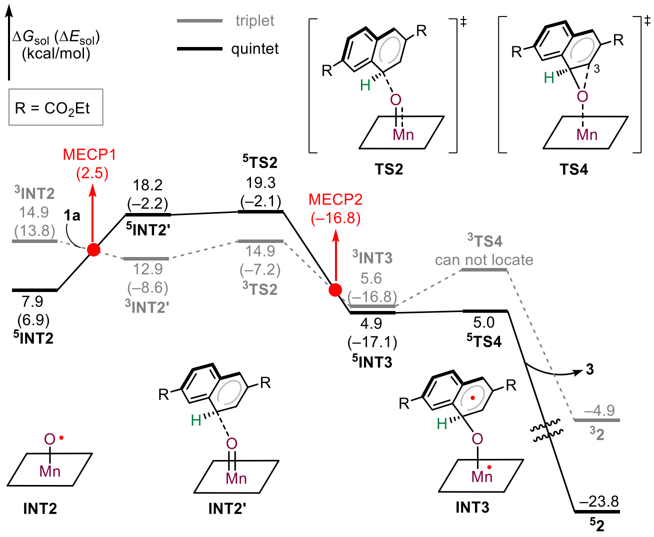

Fig. S7. The DFT-calculated possible spin states for oxygen atom attack and epoxidation process.

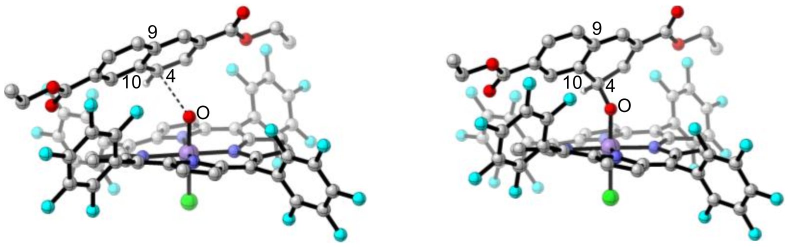

**MECP1**	**MECP2**
ΔE_{sol} = 2.5 kcal/mol	ΔE_{sol} = –16.8 kcal/mol
Mn-O = 1.78	Mn-O = 1.79
C^4-O = 2.77	C^4-O = 1.45
C^4-H = 1.08	C^4-H = 1.09
$\varphi OC^4C^{10}C^9$ =79.3°	$\varphi OC^4C^{10}C^9$ = 88.5°

Fig. S8. The calculated minimum energy crossing point (MECP1 and MECP2). The distances are in Å.

2.2. NPA spin population of ^{5}INT2

The natural population analysis (NPA) reveals spin populations of 0.819 on O and 2.235 on Mn in **^{5}INT2**, which indicates that the species is best described as a Mn(IV)-O radical.

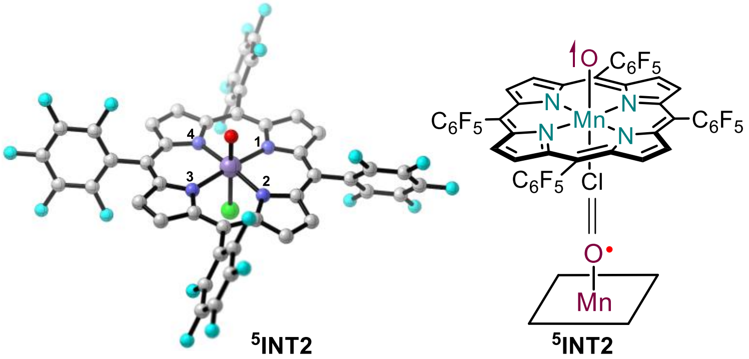

NPA Spin Population

**O**	**0.819**
**Mn**	**2.235**
**N1**	**0.122**
**N2**	**0.123**
**N3**	**0.123**
**N4**	**0.124**

Fig. S9. NPA spin population of ^{5}INT2.

2.3. The other three possibilities for radical and electrophilic pathways

For the C−O bond formation in the radical process, we calculated the three other possible oxygen atom attack transition states, **^{3}TS2-C^1** (19.7 kcal/mol), **^{3}TS2-C^2** (25.0 kcal/mol), and **^{3}TS2-C^3** (17.6 kcal/mol). All are significantly higher in energy than **^{3}TS2** (14.9 kcal/mol).

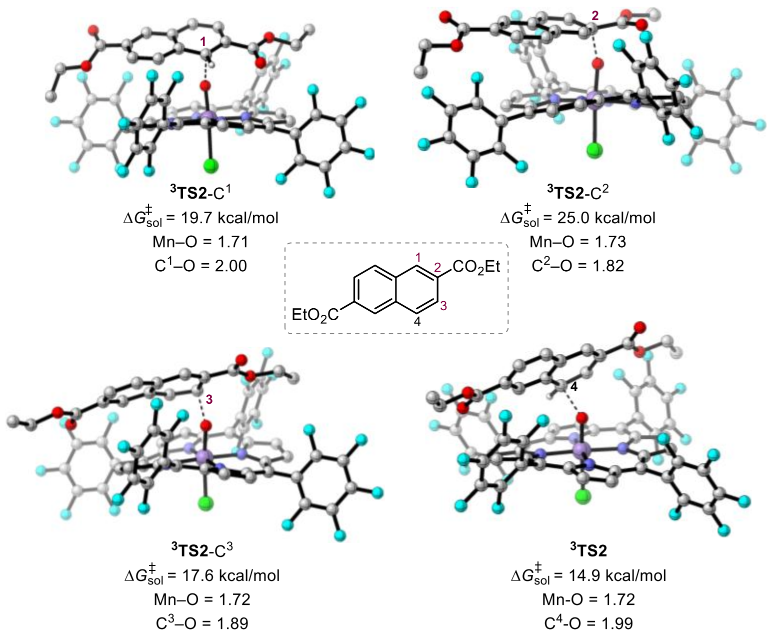

Fig. S10. The possible radical attack pathways.

For the electrophilic pathway, we also explored transition states for oxygen atom attack at C^1, C^2, and C^3. Despite extensive efforts, these transition states could not be located. Analysis showed that the intermediates formed by oxygen atom attack at the C^1, C^2, C^3, and C^4 sites of the substrate each exhibit five resonance structures. Notably, the C^1/C^4 intermediates feature two stable resonance forms, while C^2 and C^3 support only one. Moreover, steric hindrance from the ortho substituent disfavors C^1 substitution, making C^4 the optimal attack site. This suggests that the undetected transition states for the C^1, C^2, and C^3 sites likely result from their high energy barriers.

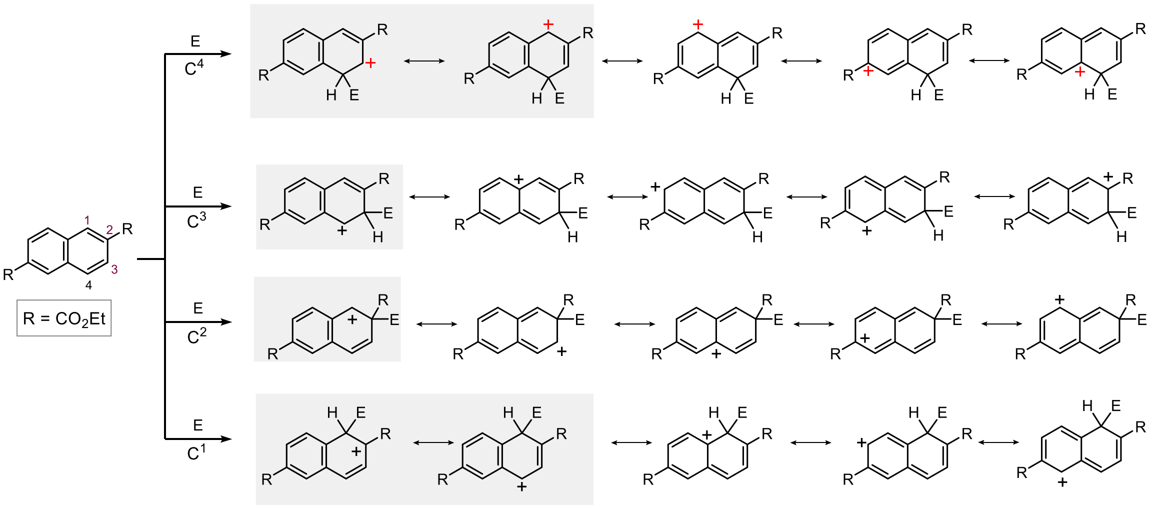

Fig. S11. Resonance structures of electrophilic oxygen atom attack at C^1, C^2, C^3, and C^4 reaction site.

2.4. Possible hydrogen atom abstraction process

The free energy profile of hydrogen atom abstraction (HAA) between the diethyl-2,6-naphthalene dicarboxylate **1** and **^{5}INT2** was given in Fig. S12. It was found that the energy of the HAA transition state **^{3}TS7** is at least 7.4 kcal/mol higher than that of the oxygen atom attack transition state **^{3}TS2** (22.3 vs. 14.9 kcal/mol), thus ruling out this pathway.

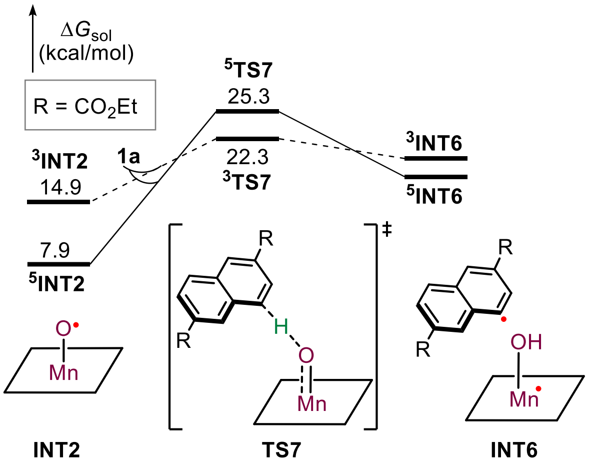

Fig. S12. The DFT-calculated free energy profile for hydrogen atom abstraction (HAA) involving aryl C(sp^2)–H bond.

2.5. Geometries and NPA charges of ^{3}TS2 and ^{3}TS3

Despite the similar geometric structures of the transition states of **^{3}TS2** and **^{3}TS3**, there are significant differences in their charge distributions. In **^{3}TS2**, the charge distributions for naphthalene and Mn-oxo porphyrin are 0.349 and −0.349, respectively, whereas in **^{3}TS3**, the charge distributions between naphthalene and Mn-oxo porphyrin are 0.650 and −0.650, respectively.

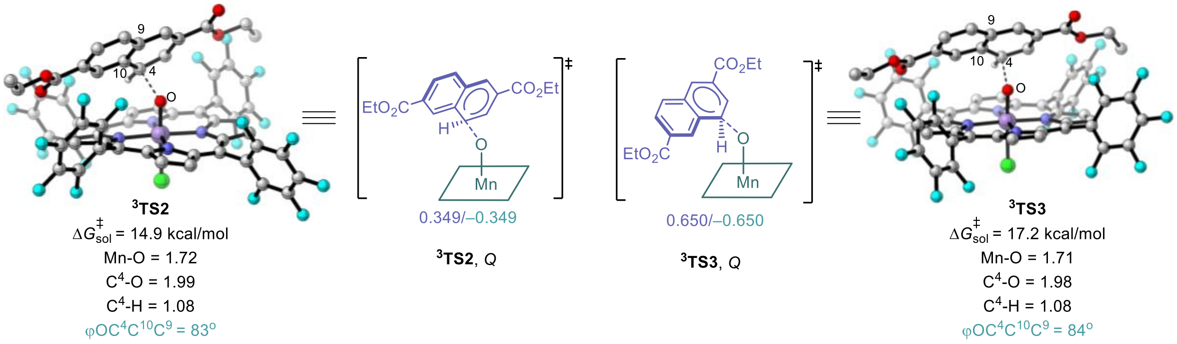

Fig. S13. Optimized geometries, NPA charges of ^{3}TS2 and ^{3}TS3. The distances are in Å.

2.6. The proton shuttle process from ^{3}INT4

Starting from **^{3}INT4**, the energy of the proton shuttle transition state **^{3}TS8** (8.5 kcal/mol) is 2.4 kcal/mol higher than that of the NIH shift transition state **^{3}TS6** (6.1 kcal/mol), indicating that the hydroxylation pathway primarily proceeds through the NIH shift process.

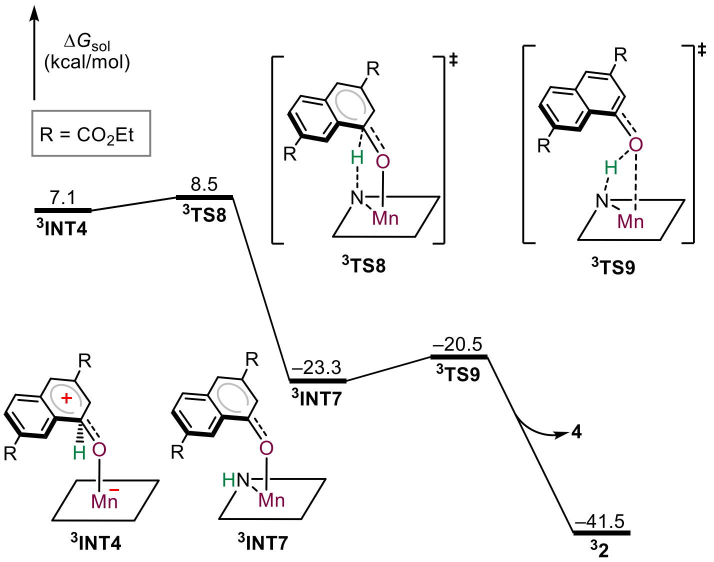

Fig. S14. The DFT-calculated free energy profile of the proton shuttle that mediated by the porphyrin.

2.7. DFT-calculated free energy profile of oxidative functionalization of arene 1s

Calculations reveal that substrates 2,7-dimethoxynaphthalene **1s** and 2,6-dimethoxynaphthalene **1t** follow the same reaction mechanism and both behave similar to the results observed with **1a**, the hydroxylation preferentially proceeds via a zwitterionic mechanism, whereas epoxidation occurs through a radical pathway. However, a reversal in reaction selectivity was observed, with hydroxylation now becoming the predominant reaction. With substrate **1s**, it was found that compared to undergoing epoxidation via the ring closure transition state **^{3}TS11** (Zwitterionic path a), **^{3}INT9** is more inclined to experience hydroxylation via the NIH shift transition state **^{3}TS12** (Zwitterionic path b) to form the **INT10**, which then undergoes the keto-enol tautomerization to give the hydroxylated product **5s'**. Epoxidation is mediated by **^{5}INT8** through the ring closure transition state **^{5}TS10** (Radical path a), rather than through the zwitterionic ring closure transition state **^{5}TS11** (Zwitterionic path a). It is noteworthy that, the energy for the **^{3}INT9** to yield hydroxylated product through **^{3}TS12** (0.5 kcal/mol) is significantly lower than that for **^{5}INT8** to afford epoxide via **^{5}TS10** (4.0 kcal/mol). The experimentally observed reversals in selectivity was well reproduced and rationalized on the basis of this mechanistic scenario.

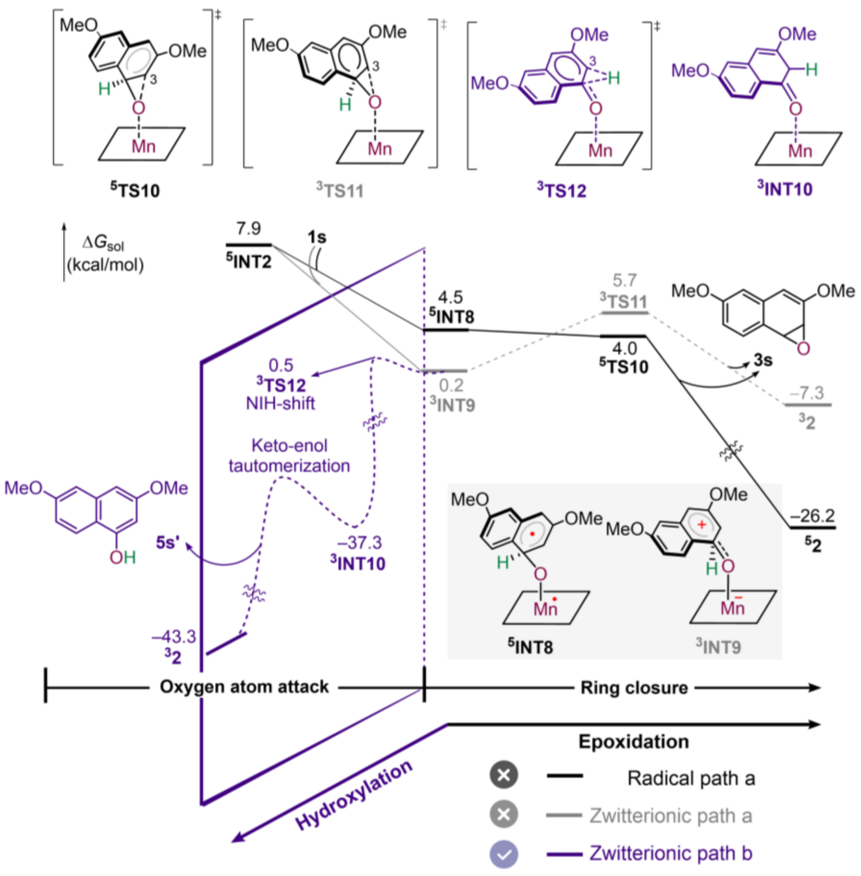

Fig. S15. DFT-calculated free energy profile of oxidative functionalization of arene 1s.

2.8. DFT-calculated free energy profile of oxidative functionalization of arene 1t

With substrate **1t**, it was found the stability of the radical and zwitterionic intermediates was reversed, resulting in the zwitterionic intermediate ^{3}**INT13**'s being 5.3 kcal/mol more stable than the radical intermediate ^{5}**INT12**, which is consistent with the trend observed for **1s**. Epoxidation is mediated by ^{5}**INT12** through the ring closure transition state ^{5}**TS15** (Radical path a), while ^{3}**INT13** is more inclined to experience hydroxylation via the NIH shift transition state ^{3}**TS17** (Zwitterionic path b) to form the **INT14**, which then undergoes the keto-enol tautomerization to give the hydroxylated product **5t'**. It is noteworthy that, the energy for the ^{3}**INT13** to yield hydroxylated product through ^{3}**TS17** (−3.5 kcal/mol) is significantly lower than that for ^{5}**INT12** to afford epoxide via ^{5}**TS15** (0.8 kcal/mol).

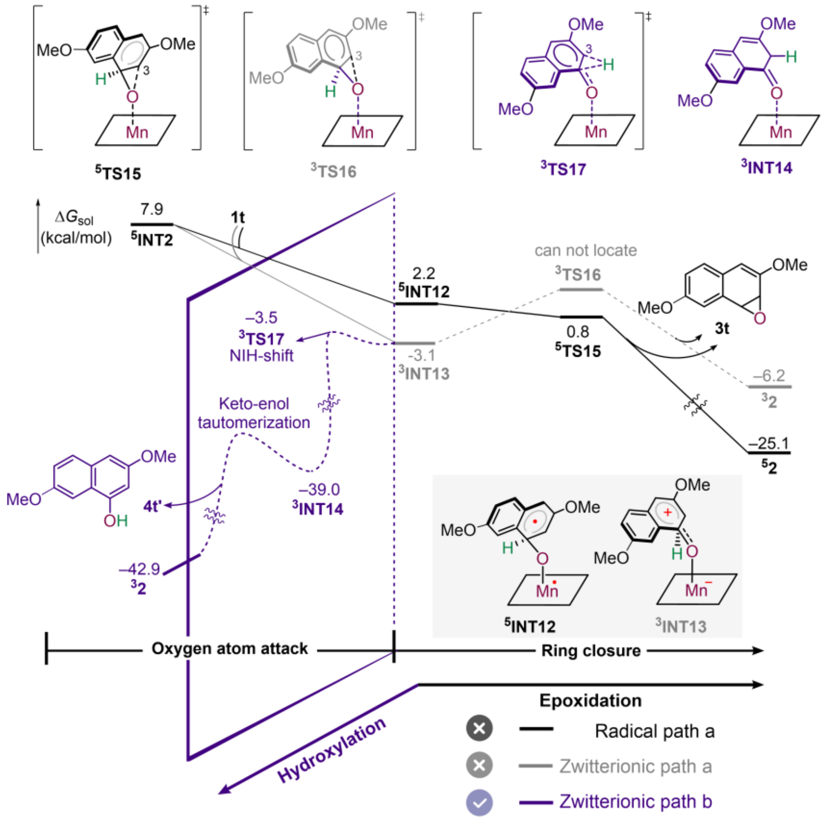

Fig. S16. DFT-calculated free energy profile of oxidative functionalization of arene 1t.

2.9. The proton shuttle process from ^{3}INT9

Starting from **^{3}INT9**, the energy of the proton shuttle transition state **^{3}TS13** (1.2 kcal/mol) is 2.4 kcal/mol higher than that of the NIH shift transition state **^{3}TS14** (0.5 kcal/mol), indicating that the hydroxylation primarily proceeds through the NIH shift process.

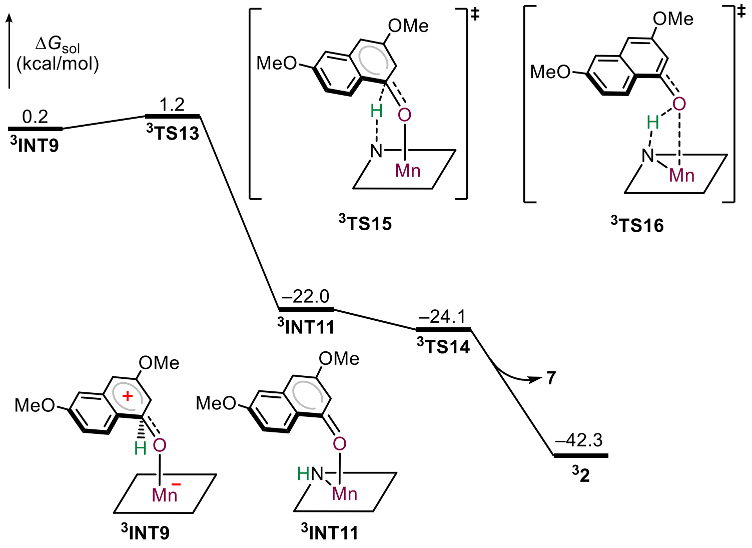

Fig. S17. The DFT-calculated free energy profile of the proton shuttle that mediated by the porphyrin ring.

2.10. The origin of substituent-controlled selectivity switch

Notably, replacing the electron-withdrawing -CO_2Et with the electron-donating -OMe reverses the stability of the radical and zwitterionic intermediates, resulting in the zwitterionic intermediate **^{3}INT9** being 4.3 kcal/mol more stable than the radical intermediate **^{5}INT8**. This stability reversal can be ascribed to the electron-donating effect, which significantly enhances the stability of **^{3}INT9** and narrows the energy gap between the ϕ orbital of **1s** ($\phi_{arene\mathbf{1s}}$) and π^*_{yz} orbital of **^{3}INT2**. The increased orbital energy of $\phi_{arene\mathbf{1s}}$, at −5.23 eV, is 0.95 eV higher than that of the $\phi_{arene\mathbf{1a}}$ (−6.18 eV), shrinking the energy gap from 3.13 to 2.18 eV. The smaller gap facilitates electron transfer from $\phi_{arene\mathbf{1s}}$ to the π^*_{yz} orbital of **^{3}INT2**, promoting formation of **^{3}INT9** and thereby reversing the observed chemoselectivity.

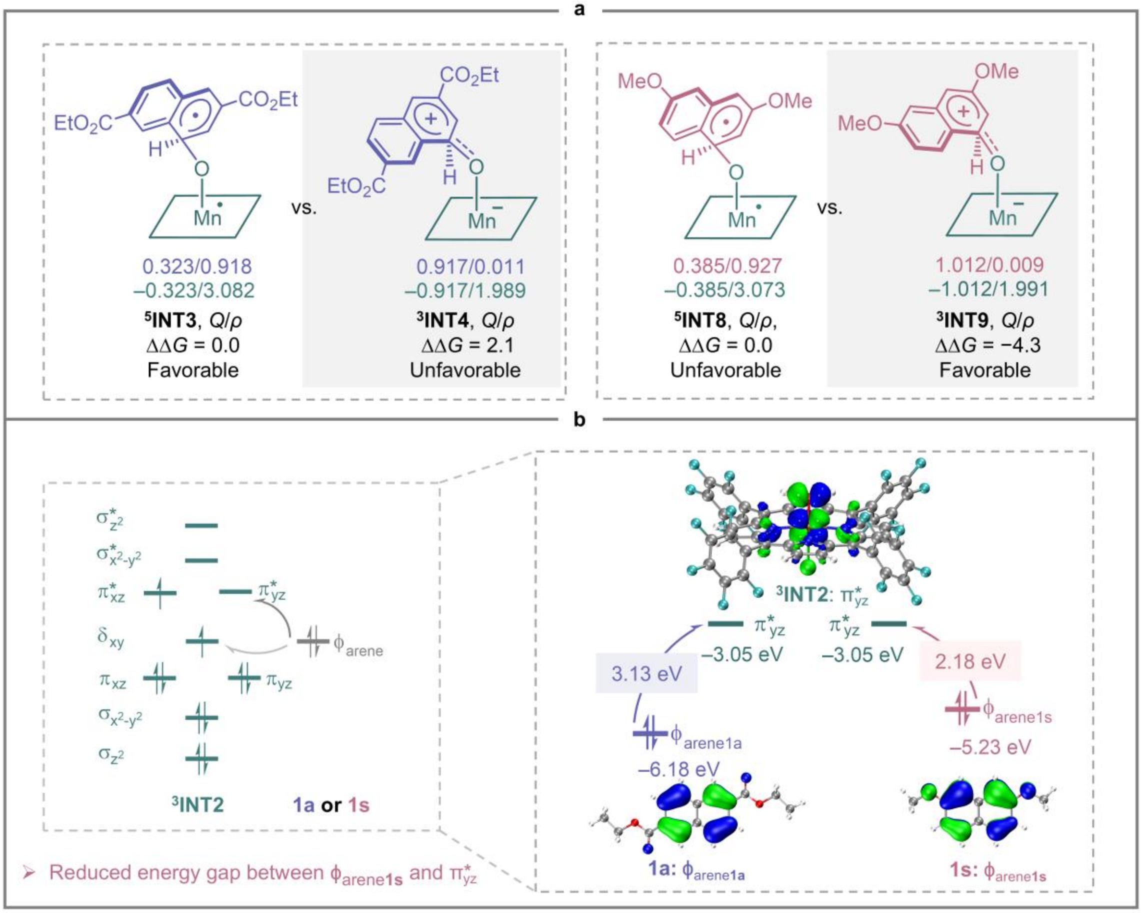

Fig. S18. The origin of substituent-controlled selectivity switch. a Key intermediates. **b** Electron transfer from ϕ orbital of arene to π^*_{yz} orbital of **INT2**.

3. References

1. Hyun, M., Jo, Y., Lee, J., Lee, H., Park, H., Hwang, I., Kim, K., Lee, S. & Kim, C. Remarkable solvent, porphyrin ligand, and aubstrate effects on participation of multiple active oxidants in manganese(III) porphyrin catalyzed oxidation reactions *Chem. Eur. J.* **19**, 1810–1818(2013).
2. Liu, W., Huang, X. Y., Placzek, M. S., Krska, S. W., McQuade, P., Hooker, J. M. & Groves, J. T. Site-selective ^{18}F fluorination of unactivated C–H bonds mediated by a manganese porphyrin. *Chem. Sci.* **9**, 1168–1172 (2018).
3. Li, G., Dilger, A. K., Cheng, P. T., Ewing, W. R. & Groves, J. T. Selective C–H halogenation with a highly fluorinated manganese porphyrin. *Angew. Chem. Int. Ed.* **57**, 1251–1255 (2018).
4. Mondal, B., Borah, D., Mazumdar, R. & Mondal, B. Nitric Oxide Dioxygenase Activity of a Nitrosyl Complex of Mn(II) Porphyrinate in the Presence of Superoxide: Formation of a Mn(IV) oxo Species through a Putative Peroxynitrite Intermediate *Inorg. Chem.* **58**, 14701−14707(2019).
5. Ge Y., Su, X., Li, G., Y, Y.-F. & S, Y. Oxidative functionalization of naphthalene derivatives catalyzed by Mn(III)-porphyrins. *Tetrahedron Lett.* **123,** 154535 (2023).
6. Furuichi, K., Tada, H., Itoh, A., Takeuchi, Y., Mitoori, M., Kato, M., Matsumoto, S. & Hashimoto, M. X-Ray structures and physical properties of tropone-annelated p-benzoquinones substituted with X-Ar groups α to tropone carbonyls and first examples of the trione substituted with an electron-withdrawing group. *J. Chem. Soc., Perkin Trans*. **2**, 1363-1378 (1994).
7. Guay, V. & Brassard, P. Synthesis of (±)-7- and 8-Hydroxydunnione. *J. Nat. Prod.* **49**, 122–125 (1986).
